# Different combinations of laccase paralogs nonredundantly control the amount and composition of lignin in specific cell types and cell wall layers in Arabidopsis

**DOI:** 10.1093/plcell/koac344

**Published:** 2022-11-30

**Authors:** Leonard Blaschek, Emiko Murozuka, Henrik Serk, Delphine Ménard, Edouard Pesquet

**Affiliations:** Arrhenius Laboratories, Department of Ecology, Environment and Plant Sciences (DEEP), Stockholm University, 106 91 Stockholm, Sweden; Arrhenius Laboratories, Department of Ecology, Environment and Plant Sciences (DEEP), Stockholm University, 106 91 Stockholm, Sweden; Umeå Plant Science Centre (UPSC), Department of Plant Physiology, Umeå University, 901 87 Umeå, Sweden; Umeå Plant Science Centre (UPSC), Department of Plant Physiology, Umeå University, 901 87 Umeå, Sweden; Arrhenius Laboratories, Department of Ecology, Environment and Plant Sciences (DEEP), Stockholm University, 106 91 Stockholm, Sweden; Umeå Plant Science Centre (UPSC), Department of Plant Physiology, Umeå University, 901 87 Umeå, Sweden; Arrhenius Laboratories, Department of Ecology, Environment and Plant Sciences (DEEP), Stockholm University, 106 91 Stockholm, Sweden; Umeå Plant Science Centre (UPSC), Department of Plant Physiology, Umeå University, 901 87 Umeå, Sweden; Bolin Centre for Climate Research, Stockholm University, 106 91 Stockholm, Sweden

## Abstract

Vascular plants reinforce the cell walls of the different xylem cell types with lignin phenolic polymers. Distinct lignin chemistries differ between each cell wall layer and each cell type to support their specific functions. Yet the mechanisms controlling the tight spatial localization of specific lignin chemistries remain unclear. Current hypotheses focus on control by monomer biosynthesis and/or export, while cell wall polymerization is viewed as random and nonlimiting. Here, we show that combinations of multiple individual laccases (LACs) are nonredundantly and specifically required to set the lignin chemistry in different cell types and their distinct cell wall layers. We dissected the roles of *Arabidopsis thaliana* LAC4, 5, 10, 12, and 17 by generating quadruple and quintuple loss-of-function mutants. Loss of these LACs in different combinations led to specific changes in lignin chemistry affecting both residue ring structures and/or aliphatic tails in specific cell types and cell wall layers. Moreover, we showed that LAC-mediated lignification has distinct functions in specific cell types, waterproofing fibers, and strengthening vessels. Altogether, we propose that the spatial control of lignin chemistry depends on different combinations of LACs with nonredundant activities immobilized in specific cell types and cell wall layers.

## Teaser

The spatial control of lignin chemistry, and thus of specific cellular functions, depends on combinations of laccases with nonredundant activities in specific cell types and cell wall layers.

**IN A NUTSHELL**

**Background:** Lignins are a diverse, complex group of aromatic polymers that accumulate in cell walls of vascular plants, reinforcing organs, and enabling long-distance water transport. The different cell wall layers of each cell type exhibit specific lignin chemistries with distinct proportions of specific aromatic substitutions and aliphatic functions. The spatial control of this lignin chemistry was supposed to depend exclusively on the chemical identity of the lignin monomers exported into the cell wall. However, monomer supply alone cannot fully explain the sharp spatial differences between each cell wall layer in the different cell types. We, therefore, investigated whether different paralogs of the lignin monomer-oxidizing LACCASE enzymes are responsible for spatially controlling lignin chemistry at the cell wall layer level for the different cell types in the vascular tissues of plants.

**Question:** How are specific lignin chemistries spatially controlled by LACCASE paralogs in each cell wall layer and cell type? What are the roles of LACCASE-dependent lignin accumulation for the mechanical reinforcement and the waterproofing of different cell types in plant vascular tissues?

**Findings:** We answered these questions by identifying the LACCASE paralogs specifically expressed in vascular cells undergoing lignin accumulation. We analyzed their functions using genetic engineering to switch off five of the six LACCASE paralog genes associated with lignin formation. Their importance in the cell wall layer and cell type lignin accumulation was determined by comparing plants sharing four of the five mutations in different LACCASE paralogs. We show that each LACCASE paralog exhibits specific substrate preference, pH optimum and localization differing between the cell wall layers of each cell type. Their lignin concentration and composition moreover depended on specific combinations of LACCASE paralogs, each enabling different aromatic substitutions and aliphatic functions to accumulate. Impairing these LACCASE-dependent lignin chemistries resulted in the loss of cell wall mechanical resistance of sap-conducting cells and the loss of cell wall waterproofing of organ-reinforcing fiber cells.

**Next steps:** We are now pursuing research to understand the molecular mechanisms controlling the supply of lignin precursors as well as the temporal regulation activating lignification during the formation/maturation of each cell wall layer in the different cell types.

## Introduction

Lignin is a complex, heterogeneous phenolic polymer that is deposited in the cell walls of specialized cell types (Meents et al., 2018). Associated with the evolutionary emergence of the plant vasculature and the transition to terrestrial habitats, lignin confers structural rigidity, and hydrophobicity to the vascular system (Eriksson et al., 1991; Ménard et al., 2022). Lignin deposition proceeds in three steps: biosynthesis of phenolic monomers, mostly phenylpropanoids, in the cytoplasm (Barros et al., 2015); their export into the apoplast (Perkins et al., 2019); and their subsequent oxidative polymerization by radical coupling catalyzed by laccases (LACs) and class III peroxidases (PRXs) in the cell wall (Blaschek and Pesquet, 2021). Among the phenoloxidases associated with lignin, specific paralogs of LACs in Arabidopsis (*Arabidopsis thaliana*) and poplar (*Populus* sp.) are the main enzymes required to accumulate lignin in vascular tissues, but their effect on lignin chemistry is not known (reviewed in Blaschek and Pesquet, 2021).

Although defined in the singular, the term lignin describes a multitude of chemically diverse polymers. This chemical diversity includes variation in both phenolic ring substitution (hydroxyphenyl, H; caffeyl C; guaicayl, G; syringyl, S) and aliphatic tail function (alcohol, X_CHOH_; aldehyde, X_CHO_) of the canonical phenylpropanoid monomers. In addition to phenylpropanoids, other monomers incorporated into lignin include benzaldehydes (Ralph et al., 2001; Chen et al., 2012), flavonoids (Lan et al., 2015; Rencoret et al., 2022), stilbenoids (del Río et al., 2017; Rencoret et al., 2019) as well as other residues containing phenyl (P) structures (Faix and Meier, 1989; Kawamoto, 2017; del Río et al., 2022). The main lignified vascular cell types in plants are tracheary elements (TEs), which act as both structural support and sap conduits for long-distance water transport. Lignin is essential for structural support at the organ level, as drastic reductions in lignin content lead to dwarf plants unable to stand upright and lodging (Bonawitz and Chapple, 2010; Muszynska et al., 2021). Lignin is equally crucial at the cellular level, as reductions in lignin levels impair the biomechanical capacity of TEs to withstand the negative pressures required to transport water (Ménard et al., 2022). In addition, most angiosperms and some gnetales also develop lignified xylem fibers located within (xylary fibers, XFs) or between (interfascicular fibers, IFs) vascular bundles, which fine-tune the mechanical properties of plant organs (Zhong and Ye, 1999).

To support their specific cellular functions, the concentration, composition, and structure of lignins are tailored to each cell type and their different cell wall layers—chemistries that are conserved between plant species (Pesquet et al., 2019). Lignin concentration is generally higher in the secondary cell walls (SCWs) of TEs than in the SCWs of fibers, as well as generally higher in primary cell walls/middle lamella (CML) and cell corners (CCs) than SCWs (Serk et al., 2015). However, large variations are also observed between different TE morphotypes, with early forming protoxylem (PX) TEs being far less lignified than later forming metaxylem (MX) TEs (Ménard et al., 2022). Lignin composition, depending in part on the proportion of monomers with different phenolic ring substitutions, varies drastically between cell types and cell wall layers, with fiber SCWs enriched in S residues, TE SCWs enriched in G residues, and CML/CCs at the interface of these two cell types enriched in H residues (Terashima and Fukushima, 1988; Fukushima and Terashima, 1990). The proportion of monomers with different aliphatic tail functions is also specifically controlled between cell types and cell wall layers, as well as between morphotypes, with high levels of X_CHO_ in MXs compared to PXs, and high levels of X_CHO_ in CMLs compared to SCWs (Peng and Westermark, 1997; Blaschek, Champagne, et al., 2020).

The lignification of each cell type progresses by incorporating distinct lignin residues at specific phases of the development and maturation of each cell type, as exemplified by S and X_CHO_ residues that are mostly incorporated in the late maturation phases of TEs compared to the early incorporation of H and G residues (Kutscha and Gray, 1972; Terashima and Fukushima, 1988; Blaschek, Champagne, et al., 2020; Ménard et al., 2022). The temporal control of cell wall lignification is specific to each cell type. TEs lignify their SCWs mostly after having committed programmed cell death (PCD) (Pesquet et al., 2010, 2013; Smith et al., 2013) whereas fibers gradually and centripetally lignify their SCWs as they are being deposited, both before and after their cell death (Fukushima and Terashima, 1991; Terashima et al., 1996; Barros et al., 2015). The mechanisms enabling such a specific spatiotemporal control of lignin chemistry in different cell types and cell wall layers are still unknown.

The current mechanisms proposed to explain this cell type/cell wall layer-specific control of lignification mainly rely on the regulation of monomer biosynthesis and/or their export to cell walls, while their polymerization is mostly viewed as random, nonlimiting, and nonspecific. Our current view of lignin formation thus posits that the oxidation of lignin monomers and their subsequent cross-coupling is mostly guided by thermodynamics and steric hindrance (Blaschek and Pesquet, 2021). However, once in the apoplast, lignin monomers are extremely mobile due to autocrine, paracrine (Pesquet et al., 2013; Smith et al., 2013, 2017), and potentially endocrine secretions (Aoki et al., 2019; Blaschek, Champagne, et al., 2020). This cell–cell transport is essential for TEs, which lignify *postmortem* (Pesquet et al., 2010, 2013; Derbyshire et al., 2015; Ménard et al., 2022) by polymerizing the monomers secreted by adjacent living cells. Neighboring fibers also use these externally supplied monomers semi-cooperatively in complement to their own export during and after the deposition of SCW polysaccharides (Barros et al., 2015; Blaschek, Champagne, et al., 2020).

The high mobility of lignin monomers in the cell wall contrasts with the spatially restricted differences of lignin chemistry in each cell wall layer of each cell type. This discrepancy suggests that additional mechanisms control lignification at the level of the cell wall itself. Such spatial control may depend on different LACs and/or PRXs with distinct catalytic activities that are targeted and immobilized in specific cell types and cell wall layers (Derbyshire et al., 2015; Blaschek and Pesquet, 2021). Lignin-associated LACs are indeed immobilized in the cell walls of lignified tissues by tight ionic bonds (Bao et al., 1993; Ranocha et al., 1999) highly limiting their mobility (Chou et al., 2018). In fact, different LACs show highly distinct binding pocket and active site topologies, suggesting differences in catalytic efficiency, pH optimum, and/or substrate specificity (Blaschek and Pesquet, 2021). LACs involved in the formation of specialized lignins in seed coats and compression wood have recently been shown to exhibit a certain degree of substrate specificity for monomers with different ring substitutions (Wang et al., 2020; Hiraide et al., 2021; Zhuo et al., 2022). The role of LAC specificity to set distinct chemistries in the main developmental lignification of xylem remains, however, unknown.

Here, we provide experimental evidence demonstrating that the lignin polymerization capacity of the different cell wall layers of each xylem cell type spatially controls its lignin chemistry. Using higher-order loss-of-function mutants of *LAC*s involved in vascular lignification, we show that the isolated function of different *LAC* paralogs is specific to each cell type and cell wall layer, and discriminately incorporates specific monomers. The resulting differences in lignin chemistry also affected its function in specific cell types, distinctly altering the cell wall mechanical resistance of TEs to negative pressure and the hydrophobicity of fiber cell walls. Altogether, we show that different immobilized combinations of LAC paralogs with specific activities control the lignin chemistry in each cell wall layer and cell type to support their different functions.

## Results

### Specific *LAC* paralogs are exclusively expressed in cells that undergo lignification

**Figure 1:**
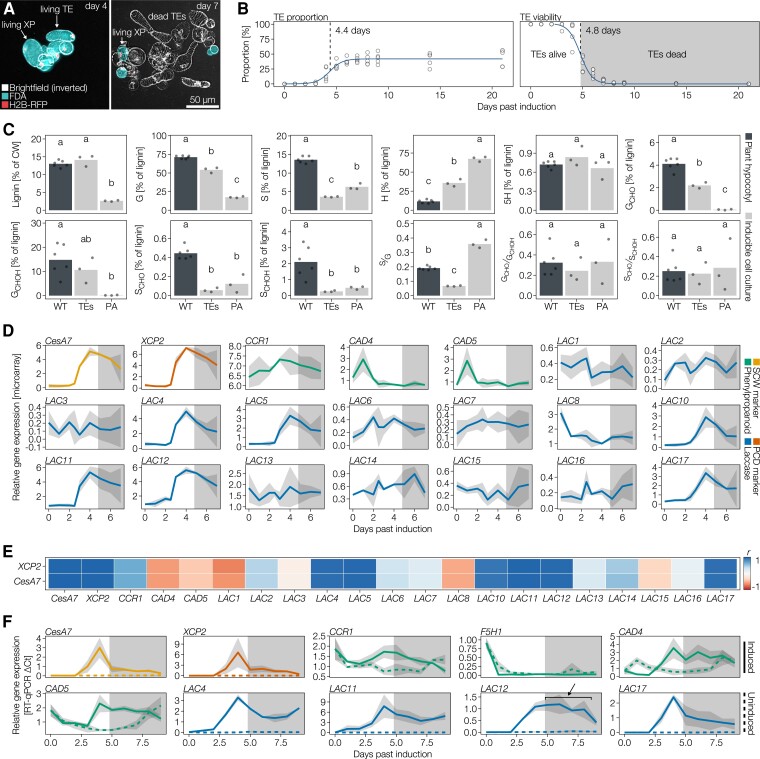
Analyses of *LAC* and phenylpropanoid genes using inducible cell cultures to relate expression to lignifying and/or nonlignifying cell walls. A, Merged images of inducible cell cultures constitutively expressing a construct encoding nucleus-localized RFP (fused to histone H2B, H2B-RFP) induced with phytohormones to differentiate into tracheary elements (TEs) and xylem parenchyma (XP) at 4 and 7 days of culture. Images correspond to merged inverted brightfield (white), FDA staining indicating living cells (turquoise) and RFP-tagged nuclei (red). Note that XP remains living once TEs have undergone PCD before 7 days of culture. B, Temporal variation of TE differentiation and TE cell death. Note that the half-way time of TE differentiation plateau to reach ∼50% of all cells was reached by 4.4 days and the half-way time of TE cell death was 4.8 days, *n* = 6 independent time courses with 200 cells counted at each time point. C, Biochemical analysis of lignin in cell walls using pyrolysis/GC–MS to determine lignin amount and chemistry between lignifying TEs, dividing parenchyma and hypocotyls of WT plants. Lignin chemistry and levels are presented as summed percentage of the total pyrogram area, with each pyrolysate identified by its *m*/*z* profiles. Note, however, that no corrections for differences in response factor were made (H residue pyrolysates have a ∼five-fold higher relative response factor than G_CHO_ and G_CHOH_, and a ∼eight-fold higher relative response factor than S_CHO_ and S_CHOH_; Van Erven et al., 2017). Different letters indicate statistically significant differences between genotypes according to a Tukey-HSD test (*α* = 0.05); *n* = 3–6 independent samples per cell type or genotype. D, Expression data of the secondary cell wall (SCW) marker gene *CESA7/IRX3*, the PCD marker gene *XCP2*, phenylpropanoid biosynthesis genes and *LAC* paralogs during TE differentiation using microarray data from Derbyshire et al. (2015). Each line represents the average of *n* = 3 independent time courses with the gray ribbon indicating SD. The shaded area of the plot indicates the time after TE PCD. CCR1 catalyzes the production of X_CHO_ phenylpropanoids (Mir Derikvand et al., 2008), CADs catalyze the production of X_CHOH_ phenylpropanoids (Sibout et al., 2005). E, Correlation analyses to define the temporal co-regulation of *LAC* paralogs and phenylpropanoid genes with markers of SCW and TE PCD. F, RT-qPCR analysis of SCW and PCD marker genes as well as *LAC* and phenylpropanoid genes during the culture time course during lignified TE formation or division of parenchyma. Line represents the average of *n* = 3 independent time courses with the gray ribbon indicating SD. The shaded area of the plot indicates the time after TE PCD. F5H1 catalyzes G_x_ to S_x_ phenylpropanoids conversion (Meyer et al., 1998). The arrow indicates extended expression of *LAC12* in induced conditions beyond TE PCD that shows expression in xylem parenchyma cells.

To discriminate which of the 17 Arabidopsis laccase paralogs are implicated in the lignification of SCWs in xylem cells, we analyzed available microarray expression data from Arabidopsis inducible pluripotent cell cultures (Derbyshire et al., 2015). This inducible system allows to trigger parenchyma cells to differentiate into TEs that undergo cell death and lignify their SCWs by adding phytohormones (Figure 1, A and B; Van de Wouwer et al., 2016; Ménard et al., 2017). Without the added phytohormones, parenchymatic cells continue to divide and remain unlignified. This on-demand triggering setup enables the user to distinguish between lignin- and non-lignin-related phenolic metabolism. Biochemical analyses of cell walls using pyrolysis coupled to gas chromatography and mass spectrometry (GC–MS) showed that unlike dividing parenchyma, 14-day-old TEs accumulate large amounts of lignin mainly composed of G residues, H, and minor amounts of S residues (Figure 1C) similarly to that previously reported for isolated TEs in other systems (Yamamura et al., 2011; Pesquet et al., 2019). We selected candidate *LAC* paralogs based on high co-expression levels with the SCW formation marker gene *CELLULOSE SYNTHASE CATALYTIC SUBUNIT A7* (*CESA7*, also named *IRREGULAR XYLEM 3* [*IRX3*]) as well as the TE autolysis marker gene *XYLEM CYSTEIN PROTEASE 2* (*XCP2*) (Ménard et al., 2015). This approach identified Arabidopsis *LAC*s *4*, *5*, *10*, *11*, *12*, and *17* as being co-upregulated during TE SCW formation and potentially implicated in vascular lignification (Figure 1, D and E). Comparison of *LAC* temporal expression with the phenylpropanoid biosynthetic genes *CINNAMOYL-COA REDUCTASE 1* (*CCR1*), *CINNAMYL ALCOHOL DEHYDROGENASE 4* (*CAD4*), and *CAD5* also revealed that selected *LAC* paralogs are more strongly co-regulated with SCW formation and PCD autolysis genes than with phenylpropanoid biosynthesis (Figure 1, D and E).

To confirm these results, we performed reverse transcription quantitative PCR (RT-qPCR) to compare *LAC* and phenylpropanoid gene expression between cell division of unlignified parenchyma and cell differentiation of lignifying TEs. We determined that all four tested *LAC* genes (*LAC4*, *LAC11*, *LAC12*, and *LAC17*) are exclusively expressed in the lignifying condition (Figure 1F), in contrast to phenylpropanoid biosynthesis genes that are expressed to similar levels in both lignifying and nonlignifying conditions as well as before and after TE cell death (Figure 1F). Interestingly, *FERULIC ACID-5-HYDROXYLASE 1* (*F5H1*) was expressed at extremely low levels in both conditions (Figure 1F), corroborating the low S residue content observed in TEs (Figure 1C). *LAC4*, *LAC11*, and *LAC17* were highly temporally coordinated with SCW formation and PCD autolysis gene expression (Figure 1F), whereas *LAC12* showed an extended expression beyond SCW and PCD marker gene expressions (Figure 1F). These differences in temporal expression between *LAC* paralogs suggest partially overlapping cell-specific expression for *LAC4*, *LAC11*, and *LAC17* exclusively expressed in TEs and *LAC12* expressed both in TEs and their specialized xylem parenchyma, even after the TEs have died (Figure 1B; Pesquet et al., 2013). In contrast to the general expression of phenylpropanoid genes, specific *LAC* paralogs are exclusively expressed in cells that will lignify.

### LAC paralogs nonredundantly and synergistically affect stem growth

The large number of implicated *LAC*s likely explains why previous studies using single loss-of-function mutants did not detect any obvious phenotypic changes (Cai et al., 2006), except for a slight *irregular xylem* (*irx*) phenotype in *lac4* (also named *irx12*) (Brown et al., 2005; Berthet et al., 2011). Eleven-day-old seedlings harboring single T-DNA insertions in individual *LAC*s exhibited no alterations in growth but did show specifically reduced or abolished production of functional transcripts from their corresponding *LAC* paralog without affecting the expression of other *LAC*s (Supplemental Figure 1, Supplemental Table 1). Our gene expression analysis detected no positive compensatory regulations between *LAC* paralogs due to the individual mutations. Indeed, we only observed significant decreases in stem height in plants that had lost the function of at least three *LAC* paralogs (Supplemental Figure 2A).

**Figure 2:**
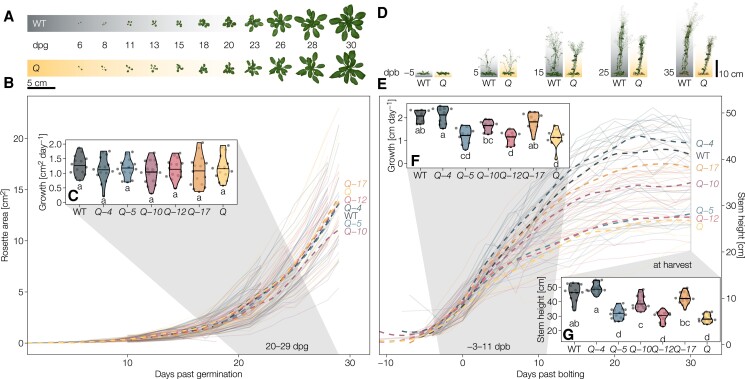
Phenotypical characterization of higher-order *lac* mutants. A, Representative rosettes of the wild-type (WT) and the *Q* (*lac4-2 lac5-1 lac10-1 lac12-2 lac17-1*) mutant between 6 days past germination (dpg) and bolting at around 30 dpg. B, Projected area of the rosettes from germination to bolting. C, Growth rates of the projected rosette areas between 20 and 29 dpg. D, Representative inflorescence stems of the WT and the *Q* mutant from 5 days before bolting to 35 days past bolting (dpb; bolting defined as reaching a height of >5 cm). E, Projected stem height (derived from a straight line from rosette to top of the plant in images) from bolting to senescence. F, Growth rates of the projected stem height from −3 to 11 dpb. G, Stem height (measured manually from the stretched out primary inflorescence stem) at harvest. Solid lines represent individual plants, dashed lines are moving regressions (LOESS) showing the genotype averages. Different letters indicate statistically significant differences between genotypes according to a Tukey-HSD test (*α* = 0.05); *n* = 10–15 individual plants from 2 to 3 independent growth instances per genotype).

To expose the functions of each individual *LAC* candidate, we generated the homozygous quintuple (*Q*) loss-of-function mutant *lac4 lac5 lac10 lac12 lac17* (Supplemental Table 2). We did not include *LAC11* in higher-order mutants due to the sterile phenotype of the *lac4 lac11 lac17* triple mutant (Zhao et al., 2013). Additionally, we failed to obtain homozygous *lac10 lac11* or *lac11 lac12* double mutants. To define the specific functions of each individual LAC, we analyzed a set of partially overlapping higher-order mutants with the quadruple homozygous loss-of-function mutants *lac5 lac10 lac12 lac17* (designated *Q*-*4*), *lac4 lac10 lac12 lac17* (*Q*-*5*), *lac4 lac5 lac12 lac17* (*Q*-*10*), *lac4 lac5 lac10 lac17* (*Q*-*12*), and *lac4 lac5 lac10 lac12* (*Q*-*17*) in comparison to the *Q* mutant and wild-type (WT) plants (Supplemental Table 2). Primary root growth and overall morphology in seedlings were unaffected by these higher-order *lac* mutants (Supplemental Figure 2). For soil-grown plants, we performed kinetic analyses using image-based phenotyping of the growth of rosette leaves and inflorescence stems (Figure 2). The growth rate of rosette leaves, the timing of bolting, number of stems as well as final stem base width were all normal in the higher-order *lac* mutants (Figure 2, A–C, Supplemental Figure 2). By contrast, stem growth rate, final height, and silique length of *Q* decreased by roughly 50%, 40%, and 10% relative to the WT, respectively (Figure 2, D–G, Supplemental Figure 2). The reduced growth rate and final height returned fully to WT levels in *Q*-*4* and *Q*-*17*, and partially in *Q*-*10* but remained comparable to *Q* levels in *Q*-*5* and *Q*-*12* (Figure 2, E–G). The reduction in stem height was accompanied in *Q*-*5*, *Q*-*12*, and *Q* by a relative increase in stem width/height, which was significantly higher than in all the other genotypes (Supplemental Figure 2). Notably, we did not observe a higher stem width/height ratio in *Q*-*10*, which caused significant stem lodging compared to other genotypes (Supplemental Figure 2). Silique length fully reached WT levels in *Q*-*4*, *Q*-*10*, and *Q*-*17*, was intermediate in *Q*-*5* but was as short as *Q* in *Q*-*12* (Supplemental Figure 2). Our results indicate a major and nonredundant contribution of LAC4, LAC10, and LAC17 in both stem and silique development, in contrast to LAC5 and LAC12. Overall, higher-order *lac* mutations mainly altered the vertical growth of inflorescence stems that suggested roles in structural support and long-distance water transport.

### Cell wall-immobilized LAC activity is cell-type- and substrate-specific

**Figure 3:**
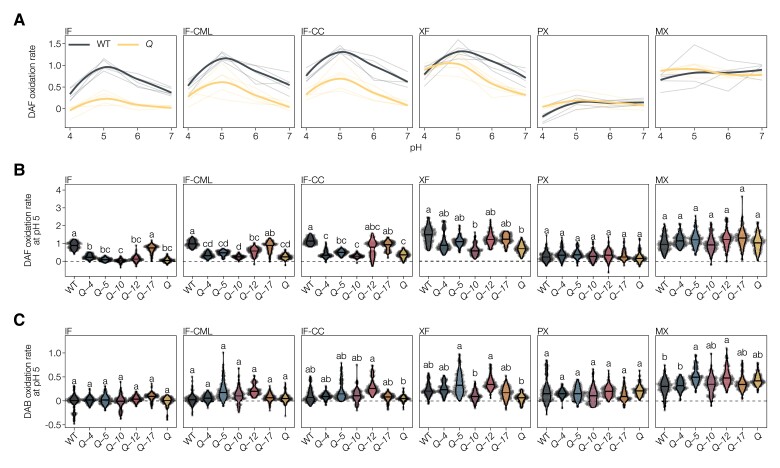
Laccase activity in the cell walls of differentiated cell types in higher-order *lac* mutants. A, Activity of WT and *Q* using DAF as substrate at pH 4–7, expressed as relative substrate oxidation rates. Thin lines represent five individual replicates, thick lines are the overall average by local regression (LOESS). Activity in autoclaved sections was subtracted to show only LAC-mediated oxidation. B, Activity of WT and higher-order *lac* mutants using DAF as substrate at pH 5, expressed as relative substrate oxidation rates. C, Activity of WT and higher-order *lac* mutants using DAF as substrate at pH 5, expressed as relative substrate oxidation rates. Different letters indicate statistically significant differences between genotypes according to a Tukey-HSD test (per panel; *α* = 0.05); 20 measurements were taken for each cell type in each of *n* = 5 individual plants per genotype.

To understand how the genetic modulation of *LAC*s resulted in the different mutant phenotypes observed above, we measured LAC activity in higher-order *lac* mutants. LAC activity was previously performed in situ but without any temporal resolution or quantitative read-out (Hiraide et al., 2021). We thus undertook a real-time imaging approach to measure oxidation rates, defined as the linear changes in intensity during time due to enzymatic substrate oxidation (Sterjiades et al., 1992), directly in the cell walls of different cell types in stem cross-sections (Supplemental Figure 3). To restrict our measurements to only cell wall-localized LAC activities, we cleared all sections with ethanol and then rehydrated them, thus avoiding artifacts from oxidation mediators (Blaschek and Pesquet, 2021), intracellular phenoloxidases (such as PRXs; Blaschek and Pesquet, 2021), and co-substrates for cell wall PRXs (H_2_O_2_ produced by wounding or hypoxia; Blokhina et al., 2001) or their removal by catalase (causing local increase in O_2_). Moreover, direct measurements in cross-sections ensured that synthetic substrates were similarly accessible to all cell types and cell wall layers. This real-time in situ activity assay also allowed us to evaluate substrate specificity, using a set of synthetic compounds with either phenylamine or phenolic groups shown to be oxidized by LACs (Supplemental Table 4; Bao et al., 1993; Richardson et al., 2000; Hiraide et al., 2021), as well as define optimal pH. Autoclaved sections served as negative controls and showed very low nonspecific binding, except for the increased auto-oxidation of the phenolic pyrogallol (PYGL) at higher neutral pH (Figure 3A and Supplemental Figure 3). With the phenylamine 2,7-diaminofluorene (DAF), LAC activity peaked at pH 5 in most cell types in WT, except for MX TEs that exhibited a stable LAC activity from pH 4 to 7, whereas PX showed almost no LAC activity (Figure 3A). The magnitude of activity at the different pH decreased in the *Q* mutant for all cell wall layers of IFs, modestly for XFs, but was unaltered for MX TEs (Figure 3A). The observed pH optimum, however, did depend on substrate, as using PYGL showed two activity optima at pH 5 and 7, which differed between cell wall layers and cell types between *Q* and WT plants (Supplemental Figure 3). These results showed that the cell walls of each cell type differ in their LAC substrate oxidation capacity and optimal pH.

Activity analyses using DAF—which was more reliable in detecting differences between genotypes than PYGL—at the optimal pH of 5 for higher-order mutants revealed that each LAC paralog specifically affects the activity profiles of the different vascular cell types (Figure 3B). In the different cell wall layers of IFs, LAC activity using DAF as substrate was almost abolished in the *Q* and most quadruple *lac* mutants and only consistently reached WT levels in *Q*-*17* (Figure 3B). Additionally, *Q*-*12* showed increased levels of LAC activity specifically in CCs and to a lesser extent in the CML compared to *Q*, but much lower levels than WT plants (Figure 3B). LAC activity with DAF as substrate remained unchanged in all mutants in both PX and MX TEs (Figure 3B). By contrast, XFs exhibited an intermediate LAC activity for DAF between IFs and TEs, with a significant reduction in the *Q* mutant, nearly reaching WT levels in the different quadruple mutants with the exception of *Q*-*10* (Figure 3B). We performed additional analyses to evaluate differences in substrate specificity of the different LACs by using a second phenylamine substrate, 3,3-diaminobenzidine (DAB). Using DAB as substrate at optimal pH of 5 in the cell wall layers of IFs showed little differences between mutants and WT except for specifically increased CC activity in *Q*-*12* (Figure 3C). MX TEs had increased activity in *Q*-*5* and *Q*-*12* (Figure 3C), indicating that LAC5 and LAC12 can use DAB as substrate in contrast to the other LAC paralogs. Lastly, XF also presented enhanced activity in *Q*-*5* and *Q*-*12* similarly to MX TEs but also a reduced activity in *Q*-*10* and *Q* compared to WT plants (Figure 3C). Our results thus confirm the effectiveness of each insertional mutation in specifically reducing LAC activity. Together, the different higher-order *lac* mutants revealed the specific localization in distinct cell wall layer and cell types of LAC paralogs. Despite their functional overlap, LAC paralogs appear only partially redundant in terms of their oxidation rate, substrate range, optimal pH condition, and spatial localization in cell walls and cell types.

### In situ quantitative chemical imaging enables to detect changes in lignin at the cellular level

**Figure 4:**
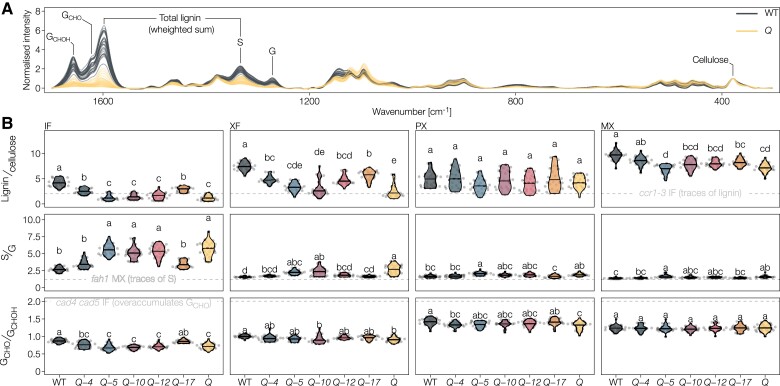
Lignin composition in higher-order *lac* mutants measured by Raman microspectroscopy. A, Raman microspectra from the IF of the WT and *Q* mutant with bands used for quantification (see Supplemental Table 5). Spectra were scaled to the cellulose band at 378 cm^−1^. B, Ratiometric characterization of lignin concentration and composition. Due to unknown relative Raman scattering coefficients of the different lignin substituents, the ratios are relative, not absolute (Agarwal et al., 2019). Well-characterized phenylpropanoid loss-of-function mutants are included as dashed gray lines to ease interpretation: *fah1* is highly reduced in S residues and increased in G_CHOH_, *ccr1-3* is highly reduced in lignin content and *cad4 cad5* is highly reduced in G_CHOH_ and overaccumulates G_CHO_. Different letters indicate statistically significant differences between genotypes according to a Tukey-HSD test (per panel; *α* = 0.05); spectra of five individual cells were measured for each cell type in each of *n* = 5 individual plants per genotype from two independent growth instances.

To quantify the specific effects of each LAC on both lignin amount and composition in the different cell types, we used Raman microspectroscopy and histochemical tests on extractive-free cross-sections of 9-week-old higher-order *lac* mutant and WT plants. Several Raman bands can be used for the relative quantification of different cell wall polymers such as total lignin and cellulose amounts, lignin S/G ratio, and total G_CHOH_ to terminal G_CHO_ ratio (Figure 4A and Supplemental Data Set 1) (Blaschek, Nuoendagula, et al., 2020; Yamamoto et al., 2020; Ménard et al., 2022). As our Raman set-up did not have the spatial resolution to distinguish between cell wall layers, WT plants exhibited the highest concentration of lignin in MX TEs, followed by XFs and then IFs and PXs (Supplemental Figure 4A). In WT plants, lignin S/G ratio was highest in IFs, followed by XFs and PXs, and then MXs (Supplemental Figure 4A), whereas the lignin G_CHO_/G_CHOH_ ratio was highest in PXs, then MXs, followed by XFs and then IFs (Supplemental Figure 4A). Such in situ quantitative chemical imaging methods are useful to prevent averaging errors when analyzing tissues with mixed cell types by reaching cellular resolutions. To validate this aspect of Raman microspectroscopy, we performed pyrolysis/GC-MS to compare the lignin composition of isolated TEs and XPs from inducible pluripotent cell cultures and that of WT hypocotyls with a mix of XPs, TEs, and fibers. Similarly to Raman measurements, isolated TEs had a low S/G ratio, whereas the TE/fiber mix in hypocotyls had a high S/G ratio, and both showed significantly more lignin than nonlignified parenchyma (Figure 1C). Raman microspectroscopy thus enables the measuring of lignin concentration and composition to the same extent as standard biochemical methods but with cellular to subcellular resolutions.

### Distinct LAC paralog nonredundantly alters lignin concentration in different cell types

The lignin-to-cellulose amount was lower for all lignified cell types in all genotypes compared to WT, except for PX where it remained unaltered (Figure 4B). The broad changes in lignin in the *Q* mutant also affected the structure of other cell wall polymers, reducing cellulose crystallinity in XFs (Supplemental Figure 4B). Among the quadruple mutants, *Q*-*4* and *Q*-*17* had the highest lignin concentrations in all tested cell types. Slight differences between these two mutants suggested a more prominent role in MX lignification for LAC4 and in fiber lignification for LAC17. The *Q*-*5*, *Q*-*10*, and *Q*-*12* mutants revealed smaller, but specific roles for the respective LACs depending on cell type. IF SCWs of *Q*-*5*, *Q*-*10*, *Q*-*12*, and *Q* had lignin-to-cellulose ratios lower than in the phenylpropanoid mutant *ccr1-3* (Mir Derikvand et al., 2008), indicating that IF lignin concentration is highly dependent on LAC4 and LAC17 (Figure 4B). In XFs, *Q*-*5* and *Q*-*10* accumulated less lignin than *Q*-*12*, which reached *Q*-*4* and *Q*-*17* levels (Figure 4B), indicating the important redundant contribution of LAC12, LAC4, and LAC17 compared to the minor contribution of LAC5 and LAC10 to XF lignification. In MXs, we observed decreases between all *lac* mutants and WT except for *Q*-*4*, with no differences between *Q*-*5* and *Q* and intermediate levels for *Q*-*10*, *Q*-*12*, and *Q-17* (Figure 4B).

To validate the observed quantitative changes obtained by Raman with a standard biochemical method used for lignin, we compared lignin levels in plant stems using pyrolysis/GC–MS. We compared *Q*-*5* to WT and the other reference lignin mutants *ccr1-3*, *fah1*, and *cad4 cad5*. Stem concentrations of lignin displayed a 53% reduction in *Q-5* compared to WT plants, similar to the 52% reduction seen in *ccr1-3*, whereas WT and *fah1* were similar and *cad4 cad5* stems accumulated 33% less lignin than WT (Supplemental Figure 5). Similar changes have previously been reported using other biochemical methods for our reference samples (Sibout et al., 2005; Van acker et al., 2013; Ménard et al., 2022), which confirmed that Raman efficiently determined quantitative changes in lignin levels. Together, our results clearly show that different LAC combinations, varying in the complement of paralogs and their individual relative contribution, are required for the levels of lignin accumulation in the cell walls of the different cell types of plant vascular tissues.

### Each LAC paralog specifically changes lignin aromatic substitution in different cell types

**Figure 5:**
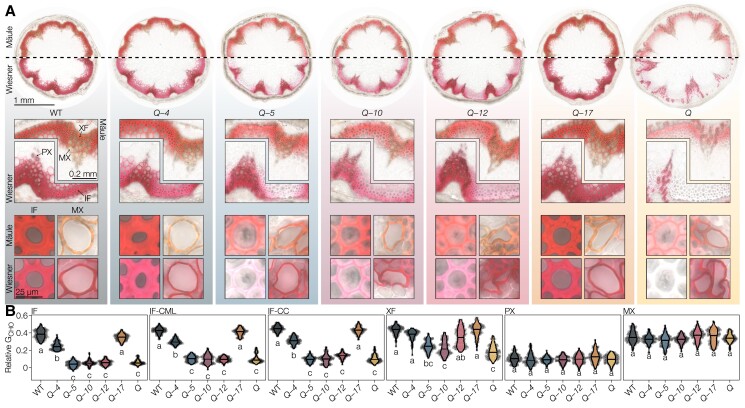
Histology of higher-order *lac* mutants. A, Mäule- and Wiesner-stained transverse stem sections at increasing levels of detail: full sections, vascular bundles with adjacent interfascicular fibers (IFs), and single cells of IF and MX. Enlarged images are provided in Supplemental Figure 6. B, Relative coniferaldehyde concentrations in IFs, compound middle lamella of IFs (IF-CML), xylary fibers (XFs), protoxylem (PX), and MX. Different letters indicate statistically significant differences between genotypes according to a Tukey-HSD test (per panel; *α* = 0.05); 20 measurements were taken for each cell type in each of *n* = 5 individual plants per genotype from two independent growth instances.

The lignin S/G ratio increased in all cell types of the *Q* mutant compared to WT plants, and the different higher-order *lac* mutants exhibited intermediate profiles varying for each LAC in magnitude and cell type (Figure 4B). SCWs of all cell types had S/G ratios greater than the phenylpropanoid mutant *fah1-2*, which only has traces of S residues (Meyer et al., 1998). The S/G changes were likely due to a large reduction in G residues rather than an increase in S residues (Supplemental Figure 4B). We also used the Mäule test that detects changes in the proportions of different ring structures by staining S residues in red and other lignin constituents in brown (Yamashita et al., 2016). Mäule-stained WT plants showed the expected intense red color in the S-enriched fiber SCWs and brown in the essentially S-depleted TEs (Figure 5A and Supplemental Figures 6 and 7). Higher-order *lac* mutants showed ∼three-fold less intense red staining in all cell wall layers of IFs in the *Q*-*5*, *Q*-*10*, *Q-12*, and *Q* mutants compared to *Q*-*4* and *Q*-*17* (Figure 5A and Supplemental Figures 6 and 7), indicating that LAC4 and LAC17 are the predominant enzymes associated with S residue accumulation in IF SCWs. Staining intensity for TEs, however, remained unaltered in all higher-order mutants (Supplemental Figure 7). Compared to the WT, slight red-shifts of 5–10 hue degrees were apparent for all mutants except *Q*-*17* in IFs and for *Q*-*5*, *Q*-*10*, *Q*-*12*, and *Q* in TEs, indicating increases in S/G ratio (Supplemental Figure 7). Regression analyses between Mäule hue and S/G ratio, determined according to Raman microspectroscopy (Agarwal et al., 2019; Blaschek, Nuoendagula, et al., 2020), showed a direct relationship between the decrease in Mäule hue (corresponding to a color shifting from brown to red) and S/G increases (Supplemental Figure 7). However, these analyses did not show any strong correlation between the Mäule test intensity and the lignin-to-cellulose content (Supplemental Figure 7). We validated our Raman S/G analyses using pyrolysis/GC–MS on ground stems: S/G ratios showed significant increases of 17% for *Q*-*5* compared to WT, due to a larger decrease of G than S residues unlike the reference samples, in agreement with the results from Raman microspectroscopy and Mäule staining (Supplemental Figures 5 and 6). In fibers, IF S/G only reached WT levels in *Q*-*4* and *Q*-*17*, whereas XF S/G attained WT levels in *Q*-*4*, *Q*-*17*, and *Q*-*12* (Figure 4B). In sap-conducting TEs, the S/G in PXs and MXs rose to WT levels in all quadruple *lac* mutants except *Q*-*5*, revealing here a similar contribution by most LACs in controlling lignin ring structure composition. As the S/G ratio differs greatly between TEs and fibers, our results indicate that different LAC paralog combinations are required for the ring structure proportions in these two different categories of cell types.

### Specific LAC paralog affects the aliphatic function of lignin residues in each cell types

The ratio of terminal G_CHO_ to total G_CHOH_ groups dropped in all cell types of the *Q* mutant compared to WT, except for MX (Figure 4B) but did not reach the G_CHO_ over-accumulation levels of the phenylpropanoid mutant *cad4 cad5* (Blaschek, Champagne, et al., 2020; Yamamoto et al., 2020). We observed intermediate profiles differing for each quadruple *lac* mutant in magnitude and cell type. In fibers, IF G_CHO_/G_CHOH_ reached WT levels fully in *Q*-*17* and was intermediate in *Q*-*4*, but remained undistinguishable from *Q* in the other quadruple mutants. XF G_CHO_/G_CHOH_ reached intermediate levels in all quadruple *lac* mutants almost to WT except for *Q*-*10* that resembled *Q* (Figure 4B). In sap-conducting TEs, PX G_CHO_/G_CHOH_ rose to WT levels in *Q*-*17*, was intermediate in *Q*-*5*, *Q*-*10*, and *Q*-*12* and similar to *Q* in *Q*-*4*, whereas MXs were not affected (Figure 4B). We also used the Wiesner test to specifically quantify total G_CHO_ concentration in situ (Blaschek, Champagne, et al., 2020). All cell wall layers of IFs showed almost no quantifiable stain in *Q*-*5*, *Q*-*10*, *Q*-*12*, and *Q*, as well as a reduction in *Q-4* when compared to WT and *Q*-*17* (Figure 5, A and B and Supplemental Figure 6). This result revealed the predominant role of LAC17, with a minor contribution by LAC4, for the incorporation of G_CHO_ in IFs. By contrast, MX TEs appeared as stained as WT plants for all genotypes (Figure 5B), revealing that LACs are implicated in the accumulation of certain residues differently between cell types. XFs were affected similarly but not as severely as IFs in higher-order *lac* mutants, showing largely reduced but not abolished accumulations of G_CHO_ in *Q*-*5*, *Q*-*10*, and *Q* compared to *Q*-*4*, *Q-12*, and *Q*-*17* (Figure 5B). This observation indicated that LAC4, LAC17, and LAC12 could contribute to reaching the G_CHO_ accumulation levels seen in XFs of WT. Although PX TEs had much lower G_CHO_ levels than MX TEs in WTs, they remained similarly unaffected in all mutants (Figure 5B). The fact that the ratio of terminal G_CHO_ to total G_CHOH_ did not reach WT levels in IFs or PXs of *Q*-*4* corroborates the reduced propensity of LAC4 to incorporate G_CHO_ compared to G_CHOH_ (Figures 4B and 5B). In ground samples, pyrolysis/GC–MS analyses also showed large decreases of total G_CHO_ in *Q*-*5*, validating our two in situ quantitative chemical imaging methods (Supplemental Figure 5). Moreover, pyrolysis/GC–MS confirmed the absence of G_CHO_/G_CHOH_ changes observed using Raman between *Q*-*5* and our reference samples, highlighting that IF decreases in *Q*-*5* are averaged out by the other unaffected cell types to appear like WT (Supplemental Figure 5). Together, these results show that different combinations of LACs are required for the accumulation of residues with specific aliphatic functions in different cell types.

### Each LAC paralog changes the levels of lignin noncanonical residues in distinct cell types

Higher-order *lac* mutants also affected the incorporation of noncanonical residues, such as benzaldehydes and residues with phenyl (P) rings, to a different extent between cell types. The proportion of P residues increased in *Q* mutants for both fiber types but not in TE morphotypes (Supplemental Figure 4B). These increases were differently compensated by different LAC paralogs, with P residues dropping back to WT levels in IFs of *Q*-*4* and *Q*-*17* and in XFs of all quadruple *lac* mutants except *Q*-*10* (Supplemental Figure 4B). Benzaldehyde residues also increased in *Q* but only in IFs, which reached the low levels seen in WT in all quadruple mutants except *Q*-*5* (Supplemental Figure 4B). Pyrolysis/GC–MS analyses also detected changes in other less abundant residues, not currently quantifiable using chemical imaging methods, with significant increases in H and 5H (5-hydroxyguaiacyl) residues in *Q*-*5* compared to WT (Supplemental Figure 5). By contrast, the proportion of S residues with either alcohol (X_CHOH_) or aldehyde (X_CHO_) did change between *Q*-*5* and WT plants (Supplemental Figure 5). These results indicated that specific LAC paralogs also altered the incorporation of noncanonical residues in specific cell type. In an effort to integrate all our lignin compositional data and demonstrate the nonoverlapping role of the different LAC paralog combinations, we performed a principal component analysis (PCA) to determine the conserved and divergent effects between cell types. In TEs, higher-order *lac* mutants only slightly affected lignin levels for MXs but caused major changes in lignin concentration and chemistry for PXs (Supplemental Figure 8). This observation contrasted with the more drastic change in fibers, where each fiber type was affected differently by the loss of specific LACs and showed a greater spread of changes in lignin levels and chemistries than PXs (Supplemental Figure 8). Altogether, our results clearly show that different LACs are active in distinct cell types and cell wall layers, where they act in combination to define specific lignin chemistries.

### Distinct LAC paralogs are responsible for cell wall layer-specific lignification

**Figure 6:**
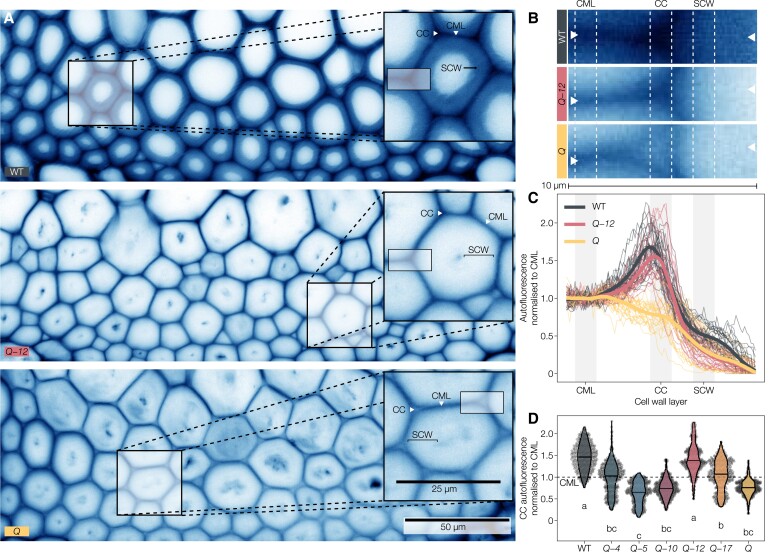
Lignin autofluorescence in IF cell corners between fibers depends on *LAC12*. A, Lignin autofluorescence of IFs in WT, *Q*-*12* (*Q* with functional *LAC12*) and *Q*. Contrast of the images was adjusted for visibility. Square insets are magnification of IF cells showing the compound middle lamella (CML), cell corners (CC), and SCW. B, Magnifications of the rectangular areas indicated in the insets of (A); arrowheads indicate endpoints of the measured line profiles shown in (C). Contrast of the images was adjusted for visibility. C, Line profiles of autofluorescence intensity normalized to the compound middle lamella. Thin lines are individual profiles from five plants from two independent growth instances, the thick line is an average by local regression (LOESS). D, Normalized fluorescence intensity measured in the shaded stretches indicated in (B, C). Different letters indicate statistically significant differences between genotypes according to a Tukey-HSD test (per panel; *α* = 0.05); each point represents one pixel from a total of 25 line profiles in *n* = 5 individual plants per genotype from two independent growth instances.

To further investigate the cell wall layer-specific roles of different LAC paralogs indicated by the activity assays and histochemical tests (Figures 3 and 5), we characterized spatial changes of lignin accumulation in higher-order *lac* mutants between cell wall layers using the UV-excited autofluorescence of lignin combined with confocal microscopy (Décou et al., 2017). This technique has a greater spatial resolution than our Raman analysis setup, as it allows for the clear distinction between cell wall layers, as with other light microscopy methods for other lignin- or LAC-related aspects (Supplemental Figure 9; Donaldson and Knox, 2012; Pesquet et al., 2013; Serk et al., 2015; Hoffmann et al., 2020; Morel et al., 2022). We observed that lignin autofluorescence in WT IFs is highest in CCs, intermediate in the compound middle lamella, and lowest in the S1/S2 and S3 layers of SCWs (Figure 6, A–C and Supplemental Figure 10). Mirroring the results of histochemical and Raman spectroscopy analyses (Figures 4 and 5), SCW autofluorescence decreased drastically in all higher-order *lac* mutants except *Q*-*4* and *Q*-*17*, which exhibited similar fluorescence levels as WT plants (Supplemental Figure 10). Spatial analyses of SCWs to distinguish S1/S2 and S3 layers revealed that *Q*-*4* has WT-like lignin fluorescence intensity in the S3, but not the S1/S2 layers, unlike *Q*-*17* with WT-like lignin fluorescences across all layers of the SCWs (Supplemental Figure 10). In the primary cell wall layers, autofluorescence from the compound middle lamella was drastically reduced in *Q*-*5*, *Q*-*10*, *Q*-*12*, and *Q* but only slightly reduced in *Q*-*4* and similar to the WT in *Q*-*17* (Supplemental Figure 10). By contrast, the autofluorescence of CCs was extremely reduced in *Q* and *Q*-*5*, *Q*-*10*, while *Q*-*12* had intermediate levels and *Q*-*4* and *Q*-*17* were reduced to levels similar to the WT CML (Supplemental Figure 10). We then measured line profiles through the different cell wall layers across the tri-cellular junction to compare changes of lignin autofluorescence across the cell wall layers of single neighboring IFs (Figure 6, B–D and Supplemental Figure 10). To control for biological and technical variation in absolute fluorescence between plants and cells, we scaled each line profile to the average of its compound middle lamella fluorescence. This approach confirmed that LAC12 activity is restricted to CCs and responsible for the specific increase in cell corner lignin autofluorescence levels relative to the compound middle lamella (Figure 6, C and D). In *Q*-*5*, *Q*-*10*, and *Q*, lignin autofluorescence in CCs was even lower than in compound middle lamellae (Figure 6D), suggesting that the LACs and/or PRXs responsible for the residual lignification of these cell wall layers are active in compound middle lamellae but not cell corners. These results showed that the lignification of IF cell wall layers depend on LAC4 and LAC17 for the S3 layer of their SCWs, LAC17 together with a minor contribution of LAC4 for the S1/S2 and compound middle lamella, and LAC4, LAC12, and LAC17 for cell corners. LAC12 was by itself capable of fully reaching a WT-like ratio for the cell corner-to-compound middle lamella lignin autofluorescence (Figure 6D and Supplemental Figure 10). Together, our results indicate that specific combinations of LAC paralogs nonredundantly control the spatially stratified lignification between the different concentric cell wall layers in vascular cells.

### Specific LAC paralogs control lignification to ensure the mechanical resistance of sap-conducting cells

**Figure 7:**
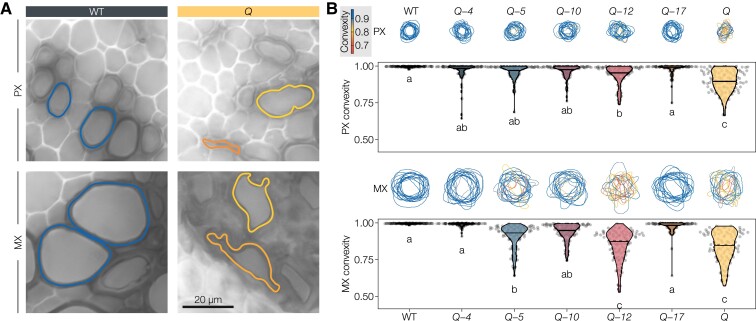
TE perimeters and their degree of inwards collapse (expressed as convexity) in the different higher-order *lac* mutants. A, Representative PX and MX perimeters in WT and *Q* sections, colored by their convexity. B, Drawn outlines of fifteen randomly sampled TEs per cell type colored by their convexity, as well as the convexities of twenty TEs of each morphotype in *n* = 5 individual plants per genotype from two independent growth instances. Different letters indicate statistically significant differences between genotypes according to a Tukey-HSD test (per panel; *α* = 0.05).

Lignin is essential for the proper vascular function of sap-conducting cells by conferring the necessary mechanical reinforcement required to sustain the negative pressure associated with sap transport (Ménard et al., 2022). As specific lignin chemistries enable each TE morphotype to conduct sap, we investigated whether the mechanical resistance of TEs was controlled by specific LACs by measuring the extent of their inward collapse in higher-order *lac* mutants. Accordingly, we measured convexity—a shape descriptor specifically characterizing inward collapse and not just general deformation (Ménard et al., 2022)—for the different TE morphotypes in stem cross-sections. TE collapse varied between genotypes as well as between TE morphotypes, revealing again that different LAC paralogs have nonredundant contribution in controlling lignin-dependent mechanical resistance of specific TEs (Figure 7). The early forming PX TEs showed some occasional collapse in all quadruple mutants but were only significantly collapsed in the absence of the activity of all five tested LACs (Figure 7). This result indicated that all investigated LAC paralogs synergistically contribute to the mechanical reinforcement of PXs. Considering the lignin composition results (Figure 4), the different LACs likely cause this effect by decreasing G_CHO_/G_CHOH_ and slightly increasing S/G (Figure 4B). The later forming MX TEs collapsed severely in *Q*, *Q*-*5*, and *Q*-*12*, to a lesser extent in *Q*-*10* and *Q*-*17*, and almost not at all in *Q*-*4* compared to the WT (Figure 7). This inward collapse appeared linked to decreased lignin levels and slight increases in S/G in MX TEs (Figure 4B). This result showed that lignin-dependent mechanical reinforcement of the MX heavily depend on LAC4, with intermediate contributions by LAC17 and LAC10, a minor role for LAC5 but no contribution of LAC12. Lastly, in the secondary xylem TEs (SX), we observed no statistically significant TE inward collapse in any of our *lac* mutants (Supplemental Figure 11). This result confirmed previous results showing that the SX is generally less prone to inward collapse (Ménard et al., 2022), and suggested that the mechanical strengthening of SX cell walls is less dependent on the tested LACs than in PX or MX. Overall, these results show that LAC paralogs nonredundantly contribute to TE lignin-dependent mechanical reinforcement, differing in both their level of contribution and TE morphotypes, with LAC4 playing the most predominant role.

### Specific LAC paralogs control lignin-dependent fiber swelling capacity

**Figure 8:**
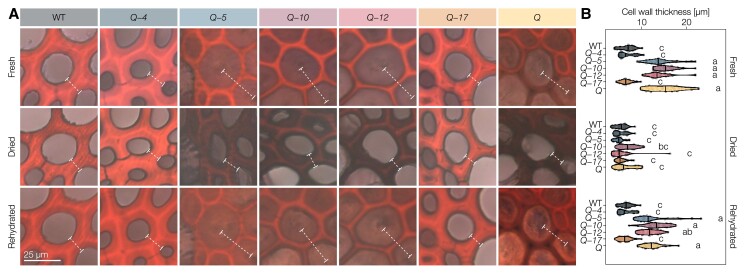
Lignin-depleted IF secondary cell walls in higher-order *lac* mutants swell in water. A, Representative Astra blue–safranin stained IF cell walls imaged fresh (never dried), dried (after air drying at room temperature overnight), and rehydrated (after incubating the dried sections in water). The thickness of the cell wall (lumen to lumen) is indicated by dashed lines. B, Thickness of 10 IF cell walls (lumen to lumen) from *n* = 3 individual plants per genotype in the three states. Different letters indicate statistically significant differences between genotypes and states according to a Tukey-HSD test (per panel; *α* = 0.05).

The stiffness and flexibility of angiosperm plant stems are controlled by lignified TEs as well as lignified fibers. Although the stiffness of TE cell walls was significantly affected by the *LAC* mutations, neither TE perimeter nor their cell wall thickness, independently of their hydration level, were altered by any of the mutations (Supplemental Figure 12). By contrast, IF SCWs in *Q*, *Q*-*5*, *Q*-*10*, and *Q-12*, all with reduced lignin amounts, were ∼two- to three-fold thicker than the more lignified IF SCWs in WT, *Q*-*4* and *Q*-*17* plants (Figure 8). To test whether this increase in cell wall thickness was due to the deposition of additional cell wall polysaccharides or to a lignin-dependent change in cell wall properties, we stained sections with safranin-O/Astra blue and imaged them fresh, after drying, and after rehydration (Figure 8). This approach allowed us to assess the proportion of IF cell wall thickness dependent on hydration. The reduced image contrast in the dried state unfortunately prevented a similar assessment of the small, densely packed XF cells. The SCWs in dried samples of all mutants presented the same thickness as WT IFs (Figure 8). This result indicated that lignin controls the hydration level of SCWs, which regulated their thicknesses (Figure 8). Rehydrating the sections confirmed this observation, as SCWs of IFs in *Q*, *Q*-*5*, *Q*-*10*, and *Q*-*12* swelled up to almost the same thickness as before drying (Figure 8). However, WT, *Q*-*4*, and *Q*-*17* had unchanged IF cell wall thicknesses in all three conditions. These results show that specific lignin chemistries in fibers of vascular tissues act as a waterproofing “varnish” to stabilize the thickness of cell walls by controlling their hydration levels, with predominant and redundant roles for LAC4 and LAC17.

## Discussion

The precise spatiotemporal control of lignification is pivotal for normal development (Zhao et al., 2013), drought resistance (Lima et al., 2018; Ménard et al., 2022), and defense against herbivores and pathogens (Whitehill et al., 2016; Joo et al., 2021). The importance of lignin monomer biosynthesis in each cell type (Dixon and Barros, 2019; Blaschek, Champagne, et al., 2020) and their transport from the cytosol into the apoplast (Perkins et al., 2019; Väisänen et al., 2020) are essential to control lignin but insufficient to regulate the strict spatial distribution of lignin in between cell types and cell wall layers. In addition to their extreme mobility in cell walls, alcohol and aldehyde phenylpropanoids have been shown to diffuse freely across biological membranes (Vermaas et al., 2019) making lignin oxidative polymerization by LACs a potential main driving force controlling the metabolic gradient-dependent transport of lignin (Perkins et al., 2022). Specific LAC paralogs had previously been shown to be important for lignin accumulation in xylem cells when knocked out or knocked down in Arabidopsis, purple false brome (*Brachypodium dystachion*), rice (*Oryza sativa*), maize (*Zea mays*), and poplar but without clearly distinguishing between cell types or morphotypes for their lignin chemistry (reviewed by Barros et al., 2015 and Blaschek and Pesquet, 2021). As specific LAC paralogs are exclusive to lignifying conditions (Figure 1) and have been suggested to drive the transport of phenylpropanoids (Perkins et al., 2022), LACs potentially represent the central regulating components capable of channeling extracellular phenolics toward lignin.

Here, we provide clear evidence that specific LAC combinations are required for depositing distinct lignin chemistries in the different cell wall layers for the specific function of each cell type. In contrast to the random oxidation model of lignin only considering thermodynamics, steric hindrance, and substrate availability as limiting factors, our results show that the identity of the oxidizing LAC establishes an important parameter for the spatial control of lignin chemistry. Our study showed that LAC4 and LAC17, previously shown to be involved in vascular lignification together with LAC11 (Berthet et al., 2011; Zhao et al., 2013), exert nonredundant functions in the lignification of specific vascular cell types. Far from being the only players, we also revealed novel and specific functions for LAC5, LAC10, and LAC12 in vascular lignification. In higher-order *lac* mutants, the loss of different LAC combinations resulted in specific and nonredundant changes of lignin chemistry affecting specific cell type and/or cell wall layer (Figures 4–6). Table 1 summarizes the different morphological and biochemical aspects observed in our higher *lac* mutants, which confirmed the substrate-specific, layer-specific, and cell type-specific activities of each of these different LACs. The drastic reduction in lignin contents in higher-order *lac* mutants, especially in fiber cell walls, demonstrated the implication of the five tested *LAC*s for vascular lignification (Figure 4B). By contrast, the stable LAC activity in MX TEs in our higher-order *lac* mutant series (Figure 3) suggests that the remaining *LAC11*, shown to be required for vascular lignification in the absence of *LAC4* and *17* (Zhao et al., 2013), suffices to ensure some lignification, although both composition and function of TEs are impaired due to their inward collapse (Figure 7). This observation confirms recent results showing that TE biomechanical properties do not only depend on lignin concentrations but also on its chemistry (Ménard et al., 2022). The resulting changes in lignin chemistry thus depend on the combined effects of reduced/abolished substrate specific-oxidation together with modifications due to nonspecific oxidation by the remaining LACs and PRXs (Blaschek and Pesquet, 2021), reduction in diffusion of some lignin precursors to cell walls due to altered metabolic gradients created by oxidation (Perkins et al., 2022), and feedback modifications of intracellular phenolic metabolism (Vanholme et al., 2012). Our results also suggest that LACs discriminate both different ring structures and aliphatic tails, as LAC4 favored the incorporation of G_CHOH_, whereas LAC17 accumulated more G_CHO_ (Figures 4 and 5).

**Table 1:** Effects of single active LACs in the *Q* background (e.g. the effect under *LAC4* describes the difference between *Q* and *Q-4*)

Structure	Parameter	*LAC4*	*LAC5*	*LAC10*	*LAC12*	*LAC17*
Plant	Stem height	WT	—	↑	—	WT
IF	Activity	—	—	—	—	↑
	Lignin	↑	—	—	—	↑
	**G**_CHO_/**G**_CHOH_	—	—	—	—	↑
	Swelling	WT	—	—	—	WT
CC	Activity	↑	—	—	↑	↑
	Lignin	↑	—	—	↑	↑
CML	Activity	↑	—	—	↑	↑
	Lignin	↑	—	—	—	↑
XF	Activity	↑	↑	—	↑	↑
	Lignin	↑	↑	—	↑	↑
	**G**_CHO_/**G**_CHOH_	↑	↑	—	↑	↑
PX	Activity	—	—	—	—	—
	Lignin	—	—	—	—	—
	**G**_CHO_/**G**_CHOH_	—	↑	↑	↑	WT
	Convexity	↑	↑	↑	↑	WT
MX	Activity	—	—	—	—	—
	Lignin	↑	—	↑	↑	↑
	**G**_CHO_/**G**_CHOH_	—	—	—	—	—
	Convexity	WT	↑	↑	—	WT

—, no effect; ↑, partly compensated; WT, reaching wild-type level.

Preferential substrate for distinct LAC paralog was shown recently by the gain-of-function of spider flower (*Cleome hassleriana*) LAC8 in Arabidopsis that enabled increased accumulation of the noncanonical C residue, with two hydroxyl groups in its aromatic ring (Wang et al., 2020; Zhuo et al., 2022). This nonredundant activity of LAC paralogs between cell types and cell wall layers (Figure 3) is moreover supported by recent protein modeling results showing that LACs have distinct protein structures affecting the position of key catalyzing amino-acids, active site binding pocket volume, shape, and accessibility (Blaschek and Pesquet, 2021). Moreover, our results clearly localized the activity of different LAC paralogs in distinct cell wall layers and cell types, such as LAC12 in the cell corners of IFs (Figures 3 and 6). The localization of specific LAC paralogs using our activity measurements showed many overlaps with previous studies using transcriptional, translational reporter, and immunolocalization (Berthet et al., 2011; Turlapati et al., 2011; Schuetz et al., 2014; Derbyshire et al., 2015; Hoffmann et al., 2020). This differential cell wall layer localization between LAC paralogs was reflected by the expression of *LAC12* in both TEs with lignified SCWs as well as in xylem parenchyma (XP) with nonlignified PCW once TEs had died (Figure 1).

Additionally, differences in localization could help support the formation of specific lignin in the “sequential intervention model” proposed by Barros et al. (2015), where phenolics are sequentially oxidized by specific LACs from their site of secretion at the plasma membrane to their site of accumulation, passing across the different layers of the cell wall. In addition to the spatial restriction of lignification, specific cell types in vascular tissues maintain distinct lignin composition and amounts across species. For example, TE enrichment in G residues is conserved among all vascular plant species (Pesquet et al., 2019). This cell type-specific function of lignin was recently demonstrated for the different TE morphotypes that require a tight regulation of G_CHO_ to G_CHOH_ ratio to maintain the balance between SCW stiffness and flexibility (Ménard et al., 2022). Here, we expand on the importance of distinct lignin chemistries for each cell types by showing that specific LACs are required for the accumulation of specific lignins to reinforce structurally TEs and limit cell wall swelling in fibers (Figures 7 and 8). Altogether, our data provide the missing link between lignin monomer biosynthetic control and their polymerization into distinct lignin polymers in specific cell types for plants to adapt to the numerous environmental and developmental stresses faced by their cell walls.

## Materials and methods

### Inducible pluripotent cell suspension cultures

Inducible pluripotent cell cultures from *A. thaliana* were used as previously described (Pesquet et al., 2010; Ménard et al., 2017) to induce on demand the chronological differentiation into TEs with phytohormones or active cell division without phytohormones. Phytohormone treatment consisted of 6 µg/ml *α*-naphthaleneacetic acid (Sigma-Aldrich, N0640), 1 µg/ml 6-benzyl-aminopurine (Sigma-Aldrich, B3408), and 4 µM 24-epibrassinolide (Sigma-Aldrich, E1641) in fresh full-strength Murashige and Skoog (MS) medium (Duchefa, M0222.0025) at pH 6.0 with 1 mM of morpholino-ethanesulfonate (Sigma-Aldrich, M8250) and 3% (w/v) sucrose supplied to 30 mg ml^−1^ of 10-day-old cell suspension (fresh weight) with synchronized cell cycle at G_0_/G_1_ due to sucrose depletion. Monitoring of cell viability was measured using inducible pluripotent cell lines stably transformed with a constitutively expressed HISTONE2B-2-RFP (AT5G22880) fusion combined with live staining with fluorescein diacetate (FDA) as described by Ménard et al. (2017). Imaging were performed using Axiovert 200M microscope (Zeiss) equipped with Colibri5 LED illumination system, an Axiocam 506 color camera and 25 × long working distance objective (NA 0.4) with either a FITC or TRITC filters for green FDA or red RFP fluorescence, respectively. Cell samples were harvested during the culture time course by vacuum filtration (Sterifil; Millipore) onto 100-µm nylon meshes and frozen in liquid nitrogen.

### Plant material

Arabidopsis (*A. thaliana*) plants from the wild-type accession Columbia-0 (Col-0) and mutants were grown in three independent rounds, each consisting of five plants per genotype, between January 2020 and July 2021 from stratified (2 days at 4°C) seeds in 1:3 vermiculite:soil in E-41L2 growth cabinets (Percival, USA). Growth conditions were cycled in a 1:6:1:16 h dusk:night:dawn:day program. Relative humidity was kept at 50%, light intensity was set to 50, 0, 50, 100 µmol m^−2^ s^−1^, and temperature at 20, 18, 20, and 22°C for dusk, night, dawn, and day, respectively. The following insertional single mutants in the Col-0 background were crossed to obtain higher-order mutants: *lac4-2* (GK-720G02-025278; Berthet et al., 2011), *lac5-1* (SALK_063466; Cai et al., 2006), *lac10-1* (SALK_017722; Cai et al., 2006), *lac11-1* (SALK_063746; Zhao et al., 2013), *lac12-2* (SALK_125379), *lac17-1* (SALK_016748; Berthet et al., 2011). Schematic representation of T-DNA insertion in each *LAC* loci is provided in Supplemental Figure 1A. Plants were confirmed to be homozygous mutants by genomic DNA extraction from two green rosette leaves using a EZNA Plant DNA kit (Omega Bio-Tek, D3485) followed by PCR genotyping using standard *Taq* DNA polymerase (18038-042, Invitrogen) to amplify sequences unique to wild-type and mutant alleles using specific primer combinations (Supplemental Table 1). PCRs used 10 ng of genomic DNA per 10-µl reaction and were cycled through 98°C (3 min), (98°C [45 s], 57°C [45 s], 72°C [90 s]) × 35 cycles, 72°C (5 min). Amplicons were separated using agarose gel electrophoresis with mini Gel II (VWR), stained with Midori Green (MG 04, Nippon Genetics), and imaged using a UV-transillumator (Biorad). Descendants of plant crosses were planted and genotyped until the desired homozygous mutant combinations were obtained. Three independent crosses were unsuccessful in obtaining the double homozygous mutants *lac10-1 lac11-1* and *lac12-2 lac11-1.* The higher-order mutants were named relative to the quintuple homozygous stacked mutant *lac4-2 lac5-1 lac10-1 lac12-2 lac17-1* or *Q* and its related quadruple homozygous mutants: *Q*-*4* is the *Q* without the *lac4-2* (rather than *Q + 4*, which defines *Q* genetically complemented with the *LAC4* wild-type gene). Supplemental Table 2 summarizes the correspondence between names and genotypes of the different quadruple mutants. Homozygous phenylpropanoid mutants used as references were *fah1* (EMS mutant; Meyer et al., 1998), *ccr1-3* (SALK_123-689; Mir Derikvand et al., 2008), and *cad4 cad5* (SAIL_1265_A06; SAIL_776_B06; Blaschek, Champagne, et al., 2020). After 9 weeks of growth, plants were harvested. The basal 2 cm of the primary inflorescence stem was stored in 70% (v/v) ethanol at −20°C until sectioning. We used 70% (v/v) ethanol to combine tissue fixation with extraction of metabolites and protoplasts instead of formaldehyde or glutaraldehyde fixation, which would not have removed non-cell wall components and would have increased autofluorescence. The stem bases were vacuum-infiltrated with ultrapure water, embedded in 10% (w/v) agarose (A9539, Sigma-Aldrich), and sectioned to 50-µm thickness using a VT1000s vibratome (Leica). Sections were then stored in 70% (v/v) ethanol at −20°C and rehydrated when needed with ultrapure water. Seeds for the root growth assay were surface-sterilized using 70% (v/v) ethanol followed by 5% (v/v) bleach (Klorox), plated on 0.8% (w/v) agar (20767.298, VWR) containing half-strength MS medium (pH 5.7) including vitamins (M0222, Duchefa Biochem), and stratified for 2 days in darkness at 4°C. Seedlings were grown for 11 days in 60% relative humidity and 150 µmol m^−2^ s^−1^ in 16-h/8-h long day conditions at 18°C (night) and 24°C (day).

### RNA extraction, RT-PCR and RT-qPCR

Total RNAs were extracted with TRI reagent (Sigma-Aldrich, 93289) according to Pesquet et al. (2005). In brief, TRI reagent and one-fifth volume chloroform were added directly to the material ground in liquid nitrogen (∼200 mg), samples were mixed by vortexing and centrifuged (16,300*g*; 15 min at room temperature). The supernatant was transferred to a new tube, one-half volume of isopropanol was added, mixed by vortexing and centrifuged at 16,300*g* for 10 min at room temperature. The supernatant was discarded, the pellet was washed twice in 70% (v/v) ethanol and air-dried for 15 min. The pellet was resuspended in MilliQ water and genomic DNA was removed with DNAse I (Promega, 610A) digestion for 30 min at 37°C. RNA was re-extracted with 1 volume TRI reagent, 1 volume water, and 1 volume chloroform as described before and the absence of DNA was confirmed by PCR with *XCP1* primer pairs for 50 cycles. RNA quantity and quality were assessed on a Nanodrop 2000 spectrophotometer (Thermo Scientific, USA) and 1.5% (w/v) agarose gel electrophoresis, respectively. First-strand cDNAs were prepared with Superscript RTase (Invitrogen, 18064-014), NVdT20 primers and 1 µg of extracted RNA as template. qPCR was performed using 1:100 diluted cDNA with iQ SYBR Green Supermix (Bio-Rad, 170-8885), and 5 µM of primers on a Bio-Rad CFX 96 real-time PCR detection system. Primer sequences are listed in Supplemental Table 3. The relative expression for each gene was determined according to the Bio-Rad CFX 96 standard procedure that determined Cq values from standard curves for each primer pairs and their efficiency. Gene expression levels are represented as relative expression corresponding to the ratio of the starting quantity (SQ-value) of the gene of interest to the geometric mean of the SQ-values of three reference genes—here *18S* rRNA, *UBQ* and *EF1α* to cover all ranges of expression levels—as previously described (Vandesompele et al., 2002). The effect of the T-DNA insertion on gene expression was evaluated for each *LAC* paralog in 11-day-old seedlings for all single homozygous mutants. RT-qPCR analyses were performed using a Lightcycler 480 (Roche, Sweden) with iQ SYBR Green Supermix (Bio-Rad, 170-8885), 5 µM of primers and expressed as dCT, ddCT, and fold changes (2^−ddCT^) relatively to *EF1α* and *UBQ* reference genes and the WT. End-point RT-PCR was performed on cDNA (the same used for RT-qPCR) with 10 µM of primers flanking the T-DNA insertion site of the *lac5-1*, *lac10-1*, and *lac12-2* mutants and on wild-type, using PhusionTaq (Thermo Scientific) using the following cycles 98°C (1 min) (98°C [30 s]/58°C [30 s]/72°C [45 s]) × 35 cycles, 72°C (3 min) on a XT96 gradient thermocycler (VWR). The amplicons were analyzed on a 1% (w/v) agarose gel (A9639, Sigma) using a minigel II electrophoresis system (VWR) and imaged using a UV-transillumator (Biorad).

### Growth phenotyping

Growth kinetics of Arabidopsis were analyzed by image-based phenotyping. Images were acquired every 2–3 days under even illumination in front of a black background with a Nikon D750 camera equipped with a Sigma 50-mm F1.4 DG HSM lens. An X-Rite ColorChecker Classic Mini color card was included in each image as a color and size reference. Images were segmented and analyzed using a custom script within the PlantCV (v. 3.8) framework (Gehan et al., 2017). The scripts are available at https://github.com/leonardblaschek/plantcv. To account for slight differences in the timing of inflorescence stem bolting between growth instances, the stem growth kinetics were aligned to the time of bolting (approximated as the day the plant surpasses a height of 5 cm). During the growth kinetic, the projected stem height was derived by measuring the distance between the rosette and the highest point of the plant in the picture, to remove effects from plant stem twisting and bending. The angle of the stem base was measured in ImageJ from images at 45 or 46 days past germination, the latest point before plant stems were wrapped around sticks. The base angle was expressed as Δ90°, i.e. the absolute deviation from a perfectly vertical (90°) growth habit. Additional manual phenotyping was conducted at the time of harvest after 9 weeks of growth, measuring stem number, primary stem height, primary stem width at the base, and silique length for five mature siliques from the primary stem. At harvest, stem height was measured manually on the stretched out stem to eliminate the effect of bends and twists.

### Laccase activity assays

Activity assays in sections were performed by vacuum infiltrating 50-µm transverse stem cross-sections (stored in 70% [v/v] ethanol at −20°C) with water for 2 h and placing them in 0.1 mM sodium acetate (pH 4, 5) or sodium phosphate (pH 6, 7) buffer in a MatTek no. 1.5 glass-bottom 96-well plate. Sections for negative controls were previously autoclaved at 121°C for 15 min in a Tuttnauer 3870EL autoclave. The final reaction volume was 200 µl and substrate concentrations were 0.07 mM DAF, 0.5 mM DAB, or 5 mM PYGL (Supplemental Table 4). Real-time images were taken every 10 min (DAF, PYGL) or 15 min (DAB) using a Zeiss Axiovert 200M equipped with a 25 × objective (NA 0.4), an Axiocam 506 color camera and an automated stage and shutters. Each section was imaged in three *Z*-positions (spaced approximately 10 µm apart) to account for any drift in the *Z*-axis. Images were aligned in Fiji (Schindelin et al., 2012) using the SIFT plugin, transformed into absorbance and measured in 20 circular areas (0.7 µm in diameter) per image and cell type. Measurements were background-corrected by subtracting the absorbance of unlignified phloem. To compare the activities across different pH, the non-enzymatic substrate oxidation rates in autoclaved sections were subtracted from the oxidation rates measured in the untreated sections in Figure 3A and Supplemental Figure 3, C–E. Comparison of activities at similar pH (Figure 3, B and C) did not require any subtraction. The corrected absorbance values were plotted against time to identify the linear phase of activity following the initial unspecific staining and before reaching a plateau of absorbance at the end of the time course. The oxidation rates of each substrate in each cell type and cell wall layer correspond to the slope of the linear changes in intensity of the oxidized product during time.

### Histochemical analysis: Wiesner stain

Cross-sections of stem bases were stained with the Wiesner test as described previously (Blaschek, Champagne, et al., 2020). Briefly, hydrated cross-sections were imaged in water using a Zeiss Axiovert 200M, equipped with a EC Plan-Neofluar 40 × objective (NA 0.75) and an automated stage, then stained by removing the cover slip and dropping 40 µl of 0.5% (w/v) phloroglucinol (P3502, Sigma-Aldrich) in a 1:1 mixture of 95% (v/v) ethanol and 37% (v/v) HCl onto the section, and imaged again after a 2-min incubation. Images were corrected for uneven illumination using a picture of an empty slide and stitched in Fiji (Preibisch et al., 2009). Absorbance values were obtained by aligning unstained and stained images, transforming the images into uncalibrated optical density and measuring circular areas (0.45 µm in diameter) of cell walls or specific layers for each cell type. The specific absorbance of the stain was obtained by subtracting the unstained absorbance from the stained absorbance for each measured point. Last, the absorbance measurements were corrected for unspecific tissue clearing by the ethanolic acid in the Wiesner reagent and/or changes in illumination by subtracting the absorbance difference between stained and unstained images of unlignified phloem from all measurements.

### Histochemical analysis: Mäule stain

Hydrated cross-sections of stem bases were incubated in 0.75% (w/v) KMnO_4_ (223468, Sigma-Aldrich) in water for 5 min, rinsed twice with water, incubated in 3 M HCl for 1 min, rinsed twice with water, and finally incubated in 10% (w/v) NaCO_3_ (71360, Sigma-Aldrich) for about 30 s. The sections were mounted in 10% (w/v) NaCO_3_ between glass slide and coverslip and imaged within 10 min after staining. Imaging and stitching were performed as described for the Wiesner stain. The absorbance of the stain was measured as described for the Wiesner test, but without correcting for unstained background. Stain hue was estimated by transforming the RGB image into the HSB colorspace and measuring circular areas (0.45 µm in diameter) of cell walls or specific layers in the hue channel. The resulting values were divided by 255 and multiplied by 360 to transform them from the 8-bit range to a 360° hue scale. Regression analyses of Mäule hue and absorbance with S/G and lignin/cellulose contents as determined by Raman microspectroscopy were done using the averages values for each cell type-genotype combination as data points.

### Raman microspectroscopy

Raman microspectroscopy was performed on hydrated cross-sections in ultrapure water between glass slide and cover slip using a LabRAM HR 800 (Horiba, France) equipped with a Nd:YAG 532 nm laser and a 50 × objective (NA 0.42). Spectra were acquired with a spectral resolution of 2 cm^−1^ from 100 to 1,800 cm^−1^. Acquisition parameters were kept constant, using a grating of 600 grooves mm^−1^, a laser power of 56 mW, and 100 accumulations of 3 s per measurement. Asymmetric least-squares baseline correction was performed using the R package “baseline” (v, 1.3-4) with parameters smoothness (λ) = 100,000 and asymmetry (*P*) = 0.01, as previously described by Blaschek, Nuoendagula, et al. (2020). To account for slight differences in instrument calibration between measuring instances, spectra were aligned to the cellulose band at 378 cm^−1^. For representation in Figure 5A, the spectra were scaled to the cellulose band at 378 cm^−1^, meaning that the band intensity in that panel is relative to total cellulose. All shown measurements of lignin composition are ratiometric, either relative to cellulose band intensity (for total lignin), relative to total lignin, or relative to other lignin constituents. For the wavenumbers of the used bands, please see Supplemental Table 5. Cellulose crystallinity was estimated according to Agarwal et al. (2010). To ease the interpretation of the Raman band ratios, measurements from well-described phenylpropanoid mutants were included: the essentially S-free *fah1* mutant (Meyer et al., 1998), the lignin deficient *ccr1-3* mutant (Mir Derikvand et al., 2008), and the G_CHO_ overaccumulating *cad4 cad5* double mutant (Blaschek, Champagne, et al., 2020).

### Lignin autofluorescence

Lignin autofluorescence in IFs was measured in hydrated cross-sections mounted between glass slide and coverslip in 50% (v/v) glycerol. Acquisition was performed using Zeiss LSM780/800 confocal laser scanning microscopes equipped with an EC Plan-Neofluar 10 × M27 (NA 0.3), a Plan-Apochromat 20 × M27 objective (NA 0.8), a Plan-Apochromat 40 × Korr M27 (NA 0.95), a 405-nm diode laser, and a long-pass emission detection filter >410 nm according to Décou et al. (2017). The assignment of the different cell wall layers was as previously described by Bond et al. (2008), Blaschek, Champagne, et al. (2020), Donaldson and Knox (2012), and Hiraide et al. (2021) for xylem cells such as IFs where each cell wall layers can be clearly distinguished by their morphology and distance from the fiber lumen. To define which magnification best enabled the precise spatial distinction of the different cell wall layers in IFs, the same cross-sections of WT and *Q* were imaged using different objectives (Supplemental Figure 9). The 20 × objective allowed significantly higher spatial resolution than the 10 × objective, while providing a better signal/noise ratio than the 40 × objective, making the 20 × objective the optimal choice for a finely resolved low-noise signal (Supplemental Figure 9). For direct comparison of fluorescence intensities (Supplemental Figure 10B), imaging parameters were kept constant between genotypes and point measurements were taken from the different cell wall layers. For the relative quantification of cell wall layer-specific autofluorescence (Figure 6, C and D and Supplemental Figure 10C) imaging parameters were adjusted between genotypes to optimize signal-to-noise ratio and line profiles were drawn through the cell wall, successively measuring adjacent CML, CC, and SCW. Only pixels from sections of the line profiles that could clearly be assigned to a specific cell wall layer were used for the violin plots comparing intensities between genotypes.

### TE collapse

The collapse of each TE morphotype was measured from the images of Wiesner stained sections as recently described by Ménard et al. (2022). TE perimeters were traced in ImageJ (Schindelin et al., 2012) using the “better wand tool” macro (https://gist.github.com/mutterer/4d3c831ca6a7e698f77eba0261b086c5) when the image contrast allowed, otherwise by freehand selection. Pixel coordinates of the perimeters were loaded into R (v. 4.10) and kernel-smoothed using the package “smoothr” (v. 0.2.2) to avoid shape artifacts where the perimeter followed the image pixelation. Area and convex hull of the smoothed perimeters were calculated using the package “sf” (v. 1.0-6), convexity representing the ratio of area to convex hull.

### Cell wall swelling

Hydrated cross-sections of stem bases were incubated in 0.01% (w/v) Astra blue (B0770, Sigma-Aldrich) in water to stain cellulose, rinsed in water, incubated in 0.01% (w/v) safranin-O (1.15948, Merck) in water to stain lignin, rinsed in water, and finally mounted between glass slide and coverslip in water to simultaneously stain all cell walls (Srebotnik and Messner, 1994). Imaging and stitching were performed as described for the Wiesner stain. After imaging, the stained sections were left to air dry at room temperature overnight and subsequently imaged again. Finally, the sections were rehydrated by incubation in water at 4°C for several days, mounted between glass slide and coverslip in water, and imaged again. Images of each section in the three stages were aligned in ImageJ. The thickness of 10 cell walls per section was measured in each state, at a point equidistant from the adjacent cell corners.

### Biochemical analysis of lignin using pyrolysis/GC–MS

Pyrolysis/GC–MS analyses were performed on 60 µg (± 10 µg) of freeze-dried ball-milled samples (pools of 8- to 9-week-old plant hypocotyls from 2 to 3 plants per replicate or vacuum-filtrated cells from 14-days old cell suspension cultures) using a PY-2020iD pyrolyzer equipped with an AS-1020E autosampler (Frontier Lab, Japan) connected to a 7890A/5975C GC/MS (Agilent, USA) as described by Gerber et al. (2012). Identification of pyrolysates was performed using combined libraries from previous works (Faix et al., 1987; Ralph and Hatfield, 1991; Gerber et al., 2012). Quantifications were made as described by Gerber et al. (2012) as summed area percentage represented in the pyrogram; note, however, that no corrections were made for differences in response factor.

### Data analysis, principal component analysis and visualization

Unless stated otherwise, all data analysis and visualization were done in R (v. 4.10), relying on the “tidyverse” (v. 1.3.1) collection of packages (Wickham et al., 2019). Statistical analyses between genotypes and cell types were performed on samples collected from different plants or cell samples grown on different rounds. ANOVA/Tukey-HSD and Kruskal–Wallis/Dunn tests were performed using the average values of each biological replicate (individual plants, specified in each Figure legend as “*n* = x”) using the “tukeygrps” package (https://github.com/leonardblaschek/tukeygrps), which uses functions from the “stats” and “dunn.test” packages. PCA was done using singular value decomposition as implemented in the “prcomp” function of the “stats” package, using data on lignin/cellulose, S/G, G_CHO_/G_CHOH_, proportion of benzaldehydes, and proportion of P residues. The R code to reproduce all analyses and Figures is available at https://github.com/leonardblaschek/Rscripts/blob/master/2021_lac_paper.rmd. Statistical data are provided in Supplemental Data Set 2.

### Accession numbers

Arabidopsis Genome Initiative numbers for the genes discussed in this article are as follows: *F5H1* (At4g36220), *CCR1* (At1g15950); *CAD4* (At4g37980); *CAD5* (At4g37990); *XCP1* (At4g35350); *XCP2* (At1g20850) *CesA7/IRX3* (At5g17420); *LAC1* (At1g18140); *LAC2* (At2g29130); *LAC3* (At2g30210); *LAC4/IRX12* (At2g38080); *LAC5* (At2g40370); *LAC6* (At2g46570); *LAC7* (At3g09220); *LAC8* (At5g01040); *LAC9* (At5g01050); *LAC10* (At5g01190); *LAC11* (At5g03260); *LAC12* (At5g05390); *LAC13* (At5g07130); *LAC14* (At5g09360); *LAC15/TT10* (At5g48100); *LAC16* (At5g58910); *LAC17* (At5g60020).

GEO accession of the microarray data used to monitor gene expression during TE differentiation in inducible pluripotent cell cultures: GSE73146.

## Supplemental Data

The following materials are available in the online version of this article.

**Supplemental Figure S1.** Effects of the used T-DNA insertions on *LAC* expression levels.

**Supplemental Figure S2.** Phenotypic characterization of higher-order *lac* mutants.

**Supplemental Figure S3.** Real-time imaging of in situ LAC activity in extractive-free cross-sections.

**Supplemental Figure S4.** Lignin chemistry measured by Raman microspectroscopy.

**Supplemental Figure S5.** Validation of the in situ quantitative chemical imaging capacity of Raman microspectroscopy compared to pyrolysis/GC–MS.

**Supplemental Figure S6.** Histochemistry of higher-order *lac* mutants.

**Supplemental Figure S7.** Mäule staining cell walls of higher-order *lac* mutants.

**Supplemental Figure S8.** Multivariate analysis of the cell type-dependent roles of different *LAC* paralogs.

**Supplemental Figure S9.** Imaging set-up necessary to measure differences in lignin autofluorescence between cell wall layers.

**Supplemental Figure S10.** Cell wall layer-specific changes in lignin autofluorescence.

**Supplemental Figure S11.** Perimeters and degree of inwards collapse of SX TEs in the different higher-order *lac* mutants.

**Supplemental Figure S12.** Cell wall properties of TEs and fibers.

**Supplemental Table S1.** Polymorphisms of the used *lac* mutant plants and the primers used to genotype them. Band sizes estimated from gel electrophoresis. KO, knockout; KD, knockdown.

**Supplemental Table S2.** Summary of the names and genotypes used to designate higher-order *lac* mutants.

**Supplemental Table S3.** Primers used for RT-qPCR analyses.

**Supplemental Table S4.** Synthetic substrates used for activity assays in sections.

**Supplemental Table S5.** Raman band intensities and intensity ratios used for cell wall characterization.

**Supplemental Data Set S1.** Summary of all data obtained in this study.

**Supplemental Data Set S2.** Summary of statistical analyses.

## Supplementary Material

koac344_Supplementary_Data

## References

[koac344-B1] Agarwal UP , Ralph SA, Padmakshan D, Liu S, Foster CE (2019) Estimation of syringyl units in wood lignins by FT-Raman spectroscopy. J Agric Food Chem 15(15): 4367–4374 10.1021/acs.jafc.8b06707 30916944

[koac344-B2] Agarwal UP , Reiner RS, Ralph SA (2010) Cellulose I crystallinity determination using FT–Raman spectroscopy: univariate and multivariate methods. Cellulose 17(4): 721–733

[koac344-B3] Aoki D , Okumura W, Akita T, Matsushita Y, Yoshida M, Sano Y, Fukushima K (2019) Microscopic distribution of syringin in freeze-fixed *Syringa vulgaris* stems. Plant Direct 3(8): e00155 31388649 10.1002/pld3.155 PMC6676449

[koac344-B4] Bao W , O’Malley DM, Whetten R, Sederoff RR (1993) A laccase associated with lignification in loblolly pine xylem. Science 260(5108): 672–674 17812228 10.1126/science.260.5108.672

[koac344-B5] Barros J , Serk H, Granlund I, Pesquet E (2015) The cell biology of lignification in higher plants. Ann Bot 115(7): 1053–1074 25878140 10.1093/aob/mcv046 PMC4648457

[koac344-B6] Berthet S , Demont-Caulet N, Pollet B, Bidzinski P, Cezard L, Le Bris P, Borrega N, Herve J, Blondet E, Balzergue S, et al (2011) Disruption of *LACCASE4* and *17* results in tissue-specific alterations to lignification of *Arabidopsis thaliana* stems. Plant Cell 23(3): 1124–1137 21447792 10.1105/tpc.110.082792 PMC3082258

[koac344-B7] Blaschek L , Champagne A, Dimotakis C, Nuoendagula Decou R, Hishiyama S, Kratzer S, Kajita S, Pesquet E (2020) Cellular and genetic regulation of coniferaldehyde incorporation in lignin of herbaceous and woody plants by quantitative Wiesner staining. Front Plant Sci 11: 109 32194582 10.3389/fpls.2020.00109 PMC7061857

[koac344-B8] Blaschek L , Nuoendagula Bacsik Z, Kajita S, Pesquet E (2020) Determining the genetic regulation and coordination of lignification in stem tissues of *Arabidopsis* using semiquantitative Raman microspectroscopy. ACS Sustain Chem Eng 8(12): 4900–4909

[koac344-B9] Blaschek L , Pesquet E (2021) Phenoloxidases in plants—how structural diversity enables functional specificity. Front Plant Sci 12: 2183 10.3389/fpls.2021.754601 PMC8517187 34659324

[koac344-B101] Blokhina OB , Chirkova TV, Fagerstedt KV (2001) Anoxic stress leads to hydrogen peroxide formation in plant cells. J Exp Bot 52(359):1179–1790 11432936

[koac344-B10] Bonawitz ND , Chapple C (2010) The genetics of lignin biosynthesis: connecting genotype to phenotype. Annu Rev Genet 44(1): 337–363 20809799 10.1146/annurev-genet-102209-163508

[koac344-B11] Bond J , Donaldson L, Hill S, Hitchcock K (2008) Safranine fluorescent staining of wood cell walls. Biotech Histochem 83(3-4): 161–171 18802812 10.1080/10520290802373354

[koac344-B12] Brown DM , Zeef LAH, Ellis J, Goodacre R, Turner SR (2005) Identification of novel genes in Arabidopsis involved in secondary cell wall formation using expression profiling and reverse genetics. Plant Cell 17(8): 2281–2295 15980264 10.1105/tpc.105.031542 PMC1182489

[koac344-B13] Cai X , Davis EJ, Ballif J, Liang M, Bushman E, Haroldsen V, Torabinejad J, Wu Y (2006) Mutant identification and characterization of the laccase gene family in *Arabidopsis*. J Exp Bot 57(11): 2563–2569 16804053 10.1093/jxb/erl022

[koac344-B14] Chen F , Tobimatsu Y, Havkin-Frenkel D, Dixon RA, Ralph J (2012) A polymer of caffeyl alcohol in plant seeds. Proc Natl Acad Sci U S A 109(5): 1772–1777 22307645 10.1073/pnas.1120992109 PMC3277123

[koac344-B15] Chou EY , Schuetz M, Hoffmann N, Watanabe Y, Sibout R, Samuels AL (2018) Distribution, mobility, and anchoring of lignin-related oxidative enzymes in Arabidopsis secondary cell walls. J Exp Bot 69(8): 1849–1859 29481639 10.1093/jxb/ery067 PMC6018803

[koac344-B16] Decou R , Serk H, Ménard D, Pesquet E. 2017. Analysis of lignin composition and distribution using fluorescence laser confocal microspectroscopy. *In*de Lucas M, Etchells JP, editors. Methods in Molecular Biology. Springer, New York, pp 233–247. 10.1007/978-1-4939-6722-3_17 28050840

[koac344-B17] del Río JC , Rencoret J, Gutiérrez A, Kim H, Ralph J (2017) Hydroxystilbenes are monomers in palm fruit endocarp lignins. Plant Physiol 174(4): 2072–2082 28588115 10.1104/pp.17.00362 PMC5543948

[koac344-B18] del Río JC , Rencoret J, Gutiérrez A, Kim H, Ralph J (2022) Unconventional lignin monomers—extension of the lignin paradigm. Adv Botanical Res 104: 1–39

[koac344-B19] Derbyshire P , Ménard D, Green P, Saalbach G, Buschmann H, Lloyd CW, Pesquet E (2015) Proteomic analysis of microtubule interacting proteins over the course of xylem tracheary element formation in Arabidopsis. Plant Cell 27: 2709–2726 26432860 10.1105/tpc.15.00314 PMC4682315

[koac344-B103] Dixon RA , Barros J (2019) Lignin biosynthesis: old roads revisited and new roads explored. Open Biol 9(12): 190215 31795915 10.1098/rsob.190215 PMC6936255

[koac344-B20] Donaldson LA , Knox JP (2012) Localization of cell wall polysaccharides in normal and compression wood of radiata pine: relationships with lignification and microfibril orientation. Plant Physiol 158(2): 642–653 22147521 10.1104/pp.111.184036 PMC3271756

[koac344-B21] Eriksson I , Haglind I, Lidbrandt O, Salmén LL (1991) Fiber swelling favoured by lignin softening. Wood Sci Technol 25(2): 135–144

[koac344-B22] Faix O , Meier D (1989) Pyrolytic and hydrogenolytic degradation studies on lignocellulosics, pulps and lignins. Holz als Roh-und Werkstoff 47(2): 67–72

[koac344-B104] Faix O , Meier D, Grobe I (1987) Studies on isolated lignins and lignins in woody materials by pyrolysis-gas chromatography-mass spectrometry and off-line pyrolysis-gas chromatography with flame ionization detection. J Anal Appl Pyrolysis 11: 403–416

[koac344-B23] Fukushima K , Terashima N (1990) Heterogeneity in formation of lignin – XIII: formation of p-hydroxyphenyl lignin in various hardwoods visualized by microautoradiography. J Wood Chem Technol 10(4): 413–433

[koac344-B24] Fukushima K , Terashima N (1991) Heterogeneity in formation of lignin – XV: formation and structure of lignin in compression wood of *Pinus thunbergii Studied by Microautoradiography*. Wood Sci Technol 25(5): 371–381

[koac344-B25] Gehan MA , Fahlgren N, Abbasi A, Berry JC, Callen ST, Chavez L, Doust AN, Feldman MJ, Gilbert KB, Hodge JG, et al (2017) PlantCV v2: image analysis software for high-throughput plant phenotyping. PeerJ 5: e4088 29209576 10.7717/peerj.4088 PMC5713628

[koac344-B105] Gerber L , Eliasson M, Trygg J, Moritz T, Sundberg B (2012) Multivariate curve resolution provides a high-throughput data processing pipeline for pyrolysis-gas chromatography/mass spectrometry. J Anal Appl Pyrolysis 95: 95–100

[koac344-B26] Hiraide H , Tobimatsu Y, Yoshinaga A, Lam PY, Kobayashi M, Matsushita Y, Fukushima K, Takabe K (2021) Localised laccase activity modulates distribution of lignin polymers in gymnosperm compression wood. New Phytol 230(6): 2186–2199 33570753 10.1111/nph.17264 PMC8252379

[koac344-B27] Hoffmann N , Benske A, Betz H, Schuetz M, Samuels AL (2020) Laccases and peroxidases co-localize in lignified secondary cell walls throughout stem development. Plant Physiol 184(2): 806–822 32699027 10.1104/pp.20.00473 PMC7536695

[koac344-B28] Joo Y , Kim H, Kang M, Lee G, Choung S, Kaur H, Oh S, Choi JW, Ralph J, Baldwin IT, et al (2021) Pith-specific lignification in *Nicotiana attenuata* as a defense against a stem-boring herbivore. New Phytol 232(1): 332–344 34171146 10.1111/nph.17583

[koac344-B29] Kawamoto H (2017) Lignin pyrolysis reactions. J Wood Sci 63(2): 117–132

[koac344-B30] Kutscha NP , Gray JR (1972) The suitability of certain stains for studying lignification in balsam fir, *Abies balsamea* (L.) Mill. Tech Bull Univ Maine 53: 1–51

[koac344-B31] Lan W , Lu F, Regner M, Zhu Y, Rencoret J, Ralph SA, Zakai UI, Morreel K, Boerjan W, Ralph J (2015) Tricin, a flavonoid monomer in monocot lignification. Plant Physiol 167(4): 1284–1295 25667313 10.1104/pp.114.253757 PMC4378158

[koac344-B32] Lima TRA , Carvalho ECD, Martins FR, Oliveira RS, Miranda RS, Müller CS, Pereira L, Bittencourt PRL, Sobczak JCMSM, Gomes-Filho E, et al (2018) Lignin composition is related to xylem embolism resistance and leaf life span in trees in a tropical semiarid climate. New Phytol 219(4): 1252–1262 29767841 10.1111/nph.15211

[koac344-B33] Meents MJ , Watanabe Y, Samuels AL (2018) The cell biology of secondary cell wall biosynthesis. Ann Bot 121(6): 1107–1125 29415210 10.1093/aob/mcy005 PMC5946954

[koac344-B107] Ménard D , Blaschek L, Kriechbaum K, Lee CC, Serk H, Zhu C, Lyubartsev A, Nuoendagula, Bacsik Z, Bergström L, Mathew A, Kajita S, Pesquet E. (2022) Plant biomechanics and resilience to environmental changes are controlled by specific lignin chemistries in each vascular cell type and morphotype. Plant Cell 34(12): 4877–4896 36215679 10.1093/plcell/koac284 PMC9709985

[koac344-B35] Ménard D , Escamez S, Tuominen H, Pesquet E. (2015). Life beyond death: the formation of Xylem sap conduits. *In* Plant Programmed Cell Death. Switzerland: Springer International Publishing, pp 55–75

[koac344-B36] Ménard D , Serk H, Decou R, Pesquet E (2017) Establishment and utilization of habituated cell suspension cultures for hormone inducible xylogenesis. Methods Mol Biol 1544: 37–57 28050827 10.1007/978-1-4939-6722-3_4

[koac344-B37] Meyer K , Shirley AM, Cusumano JC, Bell-Lelong DA, Chapple C (1998) Lignin monomer composition is determined by the expression of a cytochrome P450-dependent monooxygenase in *Arabidopsis*. Proc Natl Acad Sci U S A 95(12): 6619–6623 9618461 10.1073/pnas.95.12.6619 PMC22575

[koac344-B38] Mir Derikvand M , Sierra JB, Ruel K, Pollet B, Do C-T, Thévenin J, Buffard D, Jouanin L, Lapierre C (2008) Redirection of the phenylpropanoid pathway to feruloyl malate in *Arabidopsis* mutants deficient for cinnamoyl-CoA reductase 1. Planta 227(5): 943–956 18046574 10.1007/s00425-007-0669-x

[koac344-B102] Morel O , Lion C, Neutelings G, Stefanov J, Baldacci-Cresp F, Simon C, Biot C, Hawkins S, Spriet C (2022) REPRISAL: mapping lignification dynamics using chemistry, data segmentation, and ratiometric analysis. Plant Physiol 188(2): 816–830 34687294 10.1093/plphys/kiab490 PMC8825451

[koac344-B39] Muszynska A , Guendel A, Melzer M, Tandron Moya YA, Röder MS, Rolletschek H, Rutten T, Munz E, Melz G, Ortleb S, et al (2021) A mechanistic view on lodging resistance in rye and wheat: a multiscale comparative study. Plant Biotechnol J 19(12): 2646–2661 34449959 10.1111/pbi.13689 PMC8633492

[koac344-B40] Peng F , Westermark U (1997) Distribution of coniferyl alcohol and coniferaldehyde groups in the cell wall of Spruce fibers. Holzforschung 51(6): 531–536

[koac344-B41] Perkins ML , Schuetz M, Unda F, Chen KT, Bally MB, Kulkarni JA, Yan Y, Pico J, Castellarin SD, Mansfield SD, et al (2022) Monolignol export by diffusion down a polymerization-induced concentration gradient. Plant Cell 34(5): 2080–2095 35167693 10.1093/plcell/koac051 PMC9048961

[koac344-B42] Perkins ML , Smith RA, Samuels L (2019) The transport of monomers during lignification in plants: anything goes but how? Curr Opin Biotechnol 56: 69–74 30347315 10.1016/j.copbio.2018.09.011

[koac344-B43] Pesquet E , Korolev AV, Calder G, Lloyd CW (2010) The microtubule-associated protein AtMAP70-5 regulates secondary wall patterning in Arabidopsis wood cells. Curr Biol 20(8): 744–749 20399097 10.1016/j.cub.2010.02.057

[koac344-B44] Pesquet E , Ranocha P, Legay S, Digonnet C, Barbier O, Pichon M, Goffner D (2005) Novel markers of xylogenesis in Zinnia are differentially regulated by auxin and cytokinin. Plant Physiol 139(4): 1821–1839 16306148 10.1104/pp.105.064337 PMC1310562

[koac344-B45] Pesquet E , Wagner A, Grabber JH (2019) Cell culture systems: invaluable tools to investigate lignin formation and cell wall properties. Curr Opin Biotechnol 56: 215–222 30849592 10.1016/j.copbio.2019.02.001

[koac344-B46] Pesquet E , Zhang B, Gorzsás A, Puhakainen T, Serk H, Escamez S, Barbier O, Gerber L, Courtois-Moreau C, Alatalo E, et al (2013) Non-cell-autonomous postmortem lignification of tracheary elements in *Zinnia elegans*. Plant Cell 25(4): 1314–1328 23572543 10.1105/tpc.113.110593 PMC3663270

[koac344-B47] Preibisch S , Saalfeld S, Tomancak P (2009) Globally optimal stitching of tiled 3D microscopic image acquisitions. Bioinformatics 25(11): 1463–1465 19346324 10.1093/bioinformatics/btp184 PMC2682522

[koac344-B106] Ralph J , Hatfield RD (1991) Pyrolysis-GC-MS characterization of forage materials. J Agri Food Chem 39: 1426–1437

[koac344-B48] Ralph J , Lapierre C, Marita JM, Kim H, Lu F, Hatfield RD, Ralph S, Chapple C, Franke R, Hemm MR, et al (2001) Elucidation of new structures in lignins of CAD- and COMT-deficient plants by NMR. Phytochemistry 57(6): 993–1003 11423146 10.1016/s0031-9422(01)00109-1

[koac344-B49] Ranocha P , McDougall G, Hawkins S, Sterjiades R, Borderies G, Stewart D, Cabanes-Macheteau M, Boudet AM, Goffner D (1999) Biochemical characterization, molecular cloning and expression of laccases - a divergent gene family - in poplar. Eur J Biochem 259(1-2): 485–495 9914531 10.1046/j.1432-1327.1999.00061.x

[koac344-B50] Rencoret J , Neiva D, Marques G, Gutiérrez A, Kim H, Gominho J, Pereira H, Ralph J, del Río JC (2019) Hydroxystilbene glucosides are incorporated into Norway Spruce Bark lignin. Plant Physiol 180(3): 1310–1321 31023874 10.1104/pp.19.00344 PMC6752895

[koac344-B51] Rencoret J , Rosado MJ, Kim H, Timokhin VI, Gutiérrez A, Bausch F, Rosenau T, Potthast A, Ralph J, del Río JC (2022) Flavonoids naringenin chalcone, naringenin, dihydrotricin, and tricin are lignin monomers in papyrus. Plant Physiol 188(1): 208–219 34662399 10.1093/plphys/kiab469 PMC8774827

[koac344-B52] Richardson A , Duncan J, McDougall GJ (2000) Oxidase activity in lignifying xylem of a taxonomically diverse range of trees: identification of a conifer laccase. Tree Physiol 20(15): 1039–1047 11305458 10.1093/treephys/20.15.1039

[koac344-B53] Schindelin J , Arganda-Carreras I, Frise E, Kaynig V, Longair M, Pietzsch T, Preibisch S, Rueden C, Saalfeld S, Schmid B, et al (2012) Fiji: an open-source platform for biological-image analysis. Nat Methods 9(7): 676–682 22743772 10.1038/nmeth.2019 PMC3855844

[koac344-B54] Schuetz M , Benske A, Smith RA, Watanabe Y, Tobimatsu Y, Ralph J, Demura T, Ellis B, Samuels AL (2014) Laccases direct lignification in the discrete secondary cell wall domains of protoxylem. Plant Physiol 166(2): 798–807 25157028 10.1104/pp.114.245597 PMC4213109

[koac344-B55] Serk H , Gorzsás A, Tuominen H, Pesquet E (2015) Cooperative lignification of xylem tracheary elements. Plant Signal Behav 10(4): e1003753 10.1080/15592324.2014.1003753 PMC4622721 25761224

[koac344-B56] Sibout R , Eudes A, Mouille G, Pollet B, Lapierre C, Jouanin L, Séguin A (2005) CINNAMYL ALCOHOL DEHYDROGENASE-C and -D are the primary genes involved in lignin biosynthesis in the floral stem of Arabidopsis. Plant Cell 17(7): 2059–2076 15937231 10.1105/tpc.105.030767 PMC1167552

[koac344-B57] Smith RA , Cass CL, Mazaheri M, Sekhon RS, Heckwolf M, Kaeppler H, de Leon N, Mansfield SD, Kaeppler SM, Sedbrook JC, et al (2017) Suppression of CINNAMOYL-CoA REDUCTASE increases the level of monolignol ferulates incorporated into maize lignins. Biotechnol Biofuels 10(1): 109 28469705 10.1186/s13068-017-0793-1 PMC5414125

[koac344-B58] Smith RA , Schuetz M, Roach M, Mansfield SD, Ellis B, Samuels L (2013) Neighboring parenchyma cells contribute to *Arabidopsis* xylem lignification, while lignification of interfascicular fibers is cell autonomous. Plant Cell 25(10): 3988–3999 24096341 10.1105/tpc.113.117176 PMC3877792

[koac344-B59] Srebotnik E , Messner K (1994) A simple method that uses differential staining and light microscopy to assess the selectivity of wood delignification by white rot fungi. Appl Environ Microbiol 60(4): 1383–1386 16349245 10.1128/aem.60.4.1383-1386.1994 PMC201488

[koac344-B108] Sterjiades R , Dean JF, Eriksson KE (1992) Laccase from sycamore maple (Acer pseudoplatanus) polymerizes monolignols. Plant Physiol 99(3): 1162–1168 16668984 10.1104/pp.99.3.1162 PMC1080598

[koac344-B60] Terashima N , Atalla RH, Ralph SA, Landucci LL, Lapierre C, Monties B (1996) New preparations of lignin polymer models under conditions that approximate cell wall lignification. II. Structural characterization of the models by thioacidolysis. Holzforschung 50(1): 9–14

[koac344-B61] Terashima N , Fukushima K (1988) Heterogeneity in formation of lignin – XI: an autoradiographic study of the heterogeneous formation and structure of pine lignin. Wood Sci Technol 22(3): 259–270

[koac344-B62] Turlapati PV , Kim KW, Davin LB, Lewis NG (2011) The laccase multigene family in *Arabidopsis thaliana*: towards addressing the mystery of their gene function(s). Planta 233(3): 439–470 21063888 10.1007/s00425-010-1298-3

[koac344-B63] Väisänen E , Takahashi J, Obudulu O, Bygdell J, Karhunen P, Blokhina O, Laitinen T, Teeri TH, Wingsle G, Fagerstedt KV, et al (2020) Hunting monolignol transporters: membrane proteomics and biochemical transport assays with membrane vesicles of Norway spruce. J Exp Bot 71(20): 6379–6395 32777074 10.1093/jxb/eraa368 PMC7586744

[koac344-B64] Vandesompele J , De Preter K, Pattyn F, Poppe B, Van Roy N, De Paepe A, Speleman F (2002) Accurate normalization of real-time quantitative RT-PCR data by geometric averaging of multiple internal control genes. Genome Biol 3(7): RESEARCH0034 10.1186/gb-2002-3-7-research0034 PMC126239 12184808

[koac344-B109] Van Acker R , Vanholme R, Storme V, Mortimer JC, Dupree P, Boerjan W (2013) Lignin biosynthesis perturbations affect secondary cell wall composition and saccharification yield in Arabidopsis thaliana. Biotechnol Biofuels 6(1): 46 23622268 10.1186/1754-6834-6-46 PMC3661393

[koac344-B110] Van de Wouwer D , Vanholme R, Decou R, Goeminne G, Audenaert D, Nguyen L, Höfer R, Pesquet E, Vanholme B, Boerjan W (2016) Chemical genetics uncovers novel inhibitors of lignification, including p-iodobenzoic acid targeting CINNAMATE-4-HYDROXYLASE. Plant Physiol 172(1): 198–220 27485881 10.1104/pp.16.00430 PMC5074639

[koac344-B65] Van Erven G , de Visser R, Merkx DWH, Strolenberg W, de Gijsel P, Gruppen H, Kabel MA (2017) Quantification of lignin and its structural features in plant biomass using 13C lignin as internal standard for pyrolysis-GC-SIM-MS. Anal Chem 89(20): 10907–10916 28926698 10.1021/acs.analchem.7b02632 PMC5647568

[koac344-B66] Vanholme R , Storme V, Vanholme B, Sundin L, Christensen JH, Goeminne G, Halpin C, Rohde A, Morreel K, Boerjan W (2012) A systems biology view of responses to lignin biosynthesis perturbations in Arabidopsis. Plant Cell 24(9): 3506–3529 23012438 10.1105/tpc.112.102574 PMC3480285

[koac344-B67] Vermaas JV , Dixon RA, Chen F, Mansfield SD, Boerjan W, Ralph J, Crowley MF, Beckham GT (2019) Passive membrane transport of lignin-related compounds. Proc Natl Acad Sci U S A 116(46): 23117–23123 31659054 10.1073/pnas.1904643116 PMC6859372

[koac344-B68] Wang X , Zhuo C, Xiao X, Wang X, Docampo-Palacios M, Chen F, Dixon RA (2020) Substrate specificity of LACCASE8 facilitates polymerization of caffeyl alcohol for C-lignin biosynthesis in the seed coat of *Cleome hassleriana*. Plant Cell 32(12): 3825–3845 33037146 10.1105/tpc.20.00598 PMC7721330

[koac344-B69] Whitehill JGA , Henderson H, Schuetz M, Skyba O, Yuen MMS, King J, Samuels AL, Mansfield SD, Bohlmann J (2016) Histology and cell wall biochemistry of stone cells in the physical defence of conifers against insects. Plant Cell Environ 39(8): 1646–1661 26474726 10.1111/pce.12654

[koac344-B70] Wickham H , Averick M, Bryan J, Chang W, McGowan L, François R, Grolemund G, Hayes A, Henry L, Hester J, et al (2019) Welcome to the tidyverse. J Open Source Software 4(43): 1686

[koac344-B71] Yamamoto M , Blaschek L, Subbotina E, Kajita S, Pesquet E (2020) Importance of lignin coniferaldehyde residues for plant properties and sustainable uses. ChemSusChem 13(17): 4400–4408 32692480 10.1002/cssc.202001242 PMC7539997

[koac344-B72] Yamamura M , Wada S, Sakakibara N, Nakatsubo T, Suzuki S, Hattori T, Takeda M, Sakurai N, Suzuki H, Shibata D, et al (2011) Occurrence of guaiacyl/p-hydroxyphenyl lignin in *Arabidopsis thaliana* T87 cells. Plant Biotechnol 28(1): 1–8

[koac344-B73] Yamashita D , Kimura S, Wada M, Takabe K (2016) Improved mäule color reaction provides more detailed information on syringyl lignin distribution in hardwood. J Wood Sci 62(2): 131–137

[koac344-B74] Zhao Q , Nakashima J, Chen F, Yin Y, Fu C, Yun J, Shao H, Wang X, Wang Z-Y, Dixon RA (2013) *LACCASE* Is necessary and nonredundant with *PEROXIDASE* for lignin polymerization during vascular development in *Arabidopsis*. Plant Cell 25(10): 3976–3987 24143805 10.1105/tpc.113.117770 PMC3877815

[koac344-B75] Zhong R , Ye ZH (1999) IFL1, a gene regulating interfascicular fiber differentiation in Arabidopsis, encodes a homeodomain-leucine zipper protein. Plant Cell 11(11): 2139 10559440 10.1105/tpc.11.11.2139 PMC144121

[koac344-B111] Zhuo C , Wang X, Docampo-Palacios M, Sanders BC, Engle NL, Tschaplinski TJ, Hendry JI, Maranas CD, Chen F, Dixon RA (2022) Developmental changes in lignin composition are driven by both monolignol supply and laccase specificity. Sci Adv 8(10):eabm8145 10.1126/sciadv.abm8145 PMC8906750 35263134

