# Supplementary material for: Different combinations of laccase paralogs nonredundantly control the amount and composition of lignin in specific cell types and cell wall layers in Arabidopsis
Source: Plant Cell. 2022 Nov 30;35(2):889–909. doi: 10.1093/plcell/koac344 (PMC9940878; doi:10.1093/plcell/koac344)
Supplement: koac344_Supplementary_Data [file koac344_supplementary_data.zip › Blaschek2022_supplement_revised.pdf]

Different combinations of laccase paralogs non-redundantly control the lignin amount and composition of specific cell types and cell wall layers in *Arabidopsis*

Leonard Blaschek[1], Emiko Murozuka[1,2,3], Henrik Serk[2], Delphine Ménard[1,2], and Edouard Pesquet[1,2,4,*]

[1]Arrhenius laboratories, Department of Ecology, Environment and Plant Sciences (DEEP), Svante Arrhenius väg 20A, Stockholm University, 160 91 Stockholm, Sweden
[2]Umeå Plant Science Centre (UPSC), Department of Plant Physiology, Umeå University, 901 87 Umeå, Sweden
[3]Present address: Carlsberg Research Laboratory, J.C. Jacobsens Gade 4, DK-1799 Copenhagen V, Denmark
[4]Bolin Centre for Climate Research, Stockholm University, 106 91 Stockholm, Sweden

* Address correspondence to edouard.pesquet@su.se

0000-0003-3943-1476 (LB), 0000-0003-4803-3664 (HS), 0000-0002-7320-7921 (DM), 0000-0002-6959-3284 (EP)

Supplementary figures

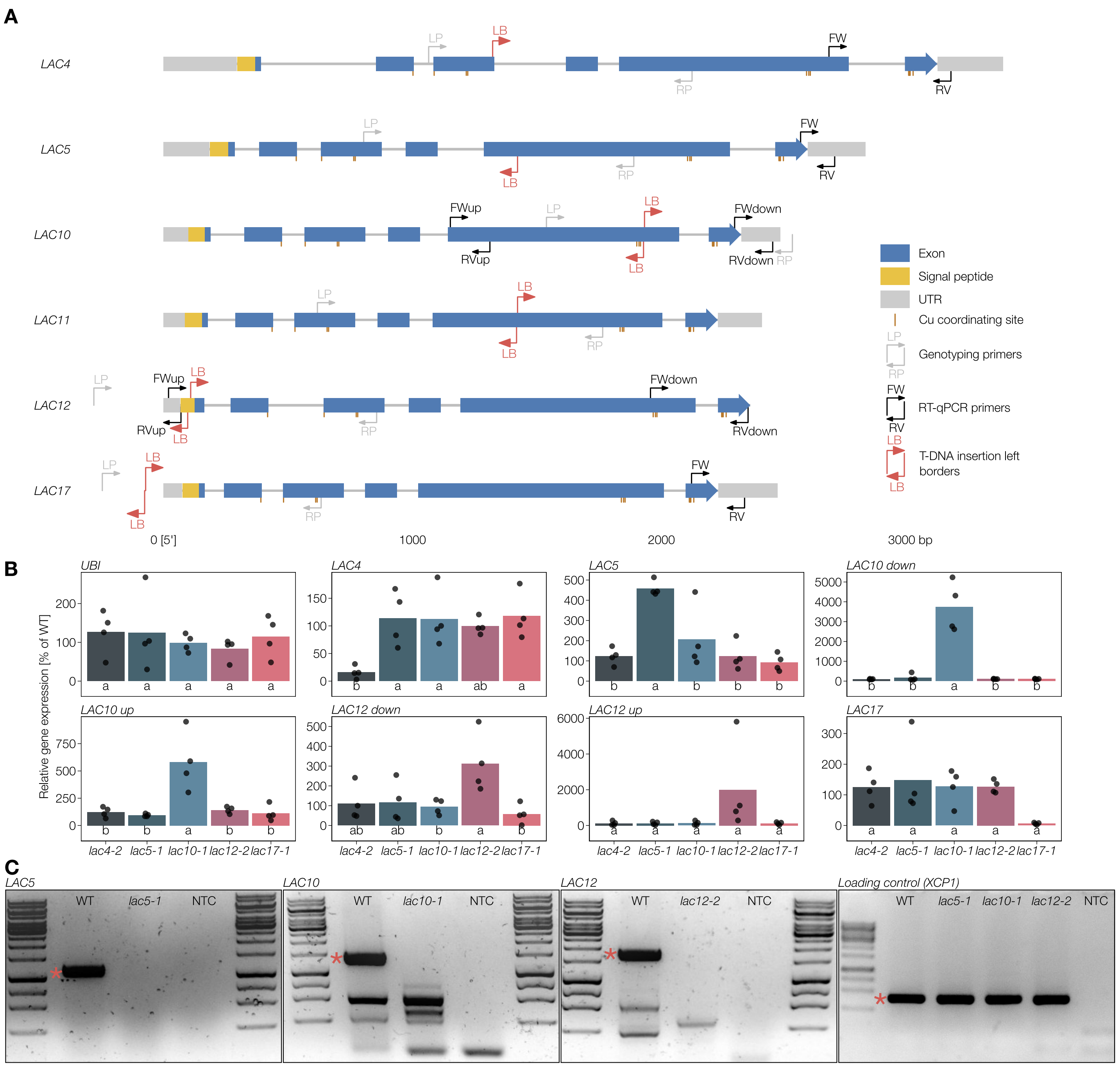

Supplemental Figure S1 | Effects of the used T-DNA insertions on LAC expression levels. (Supports Figure 2).
A, Schematic representation of LAC paralog gene structure showing intron/exon structure, 5' and 3' untranslated regions (UTRs), signal peptide, T-DNA insertion (as single or tandem insertion), as well as primer pairs for genotyping and gene expression analyses. **B**, Effect of T-DNA insertions LAC expression levels using RT-qPCR (n = 4 biological replicates from two growth instances). Note that the T-DNA insertions in lac5-1, lac10-1 and lac12-2 caused upregulation of the respective gene, which was inconsistent between amplicons upstream or downstream of the T-DNA insertion. **C**, Expression of LAC5, LAC10, LAC12 as well as the loading control gene XCP1 in wild type (WT) and the respective mutant plants determined using end-point RT-PCR with primers flanking the T-DNA insertion. The target amplicons (marked with a red asterisk) were absent in the mutant plants, confirming that the T-DNA insertion abolishes the production of the respective full-length LAC transcripts in the used lines.

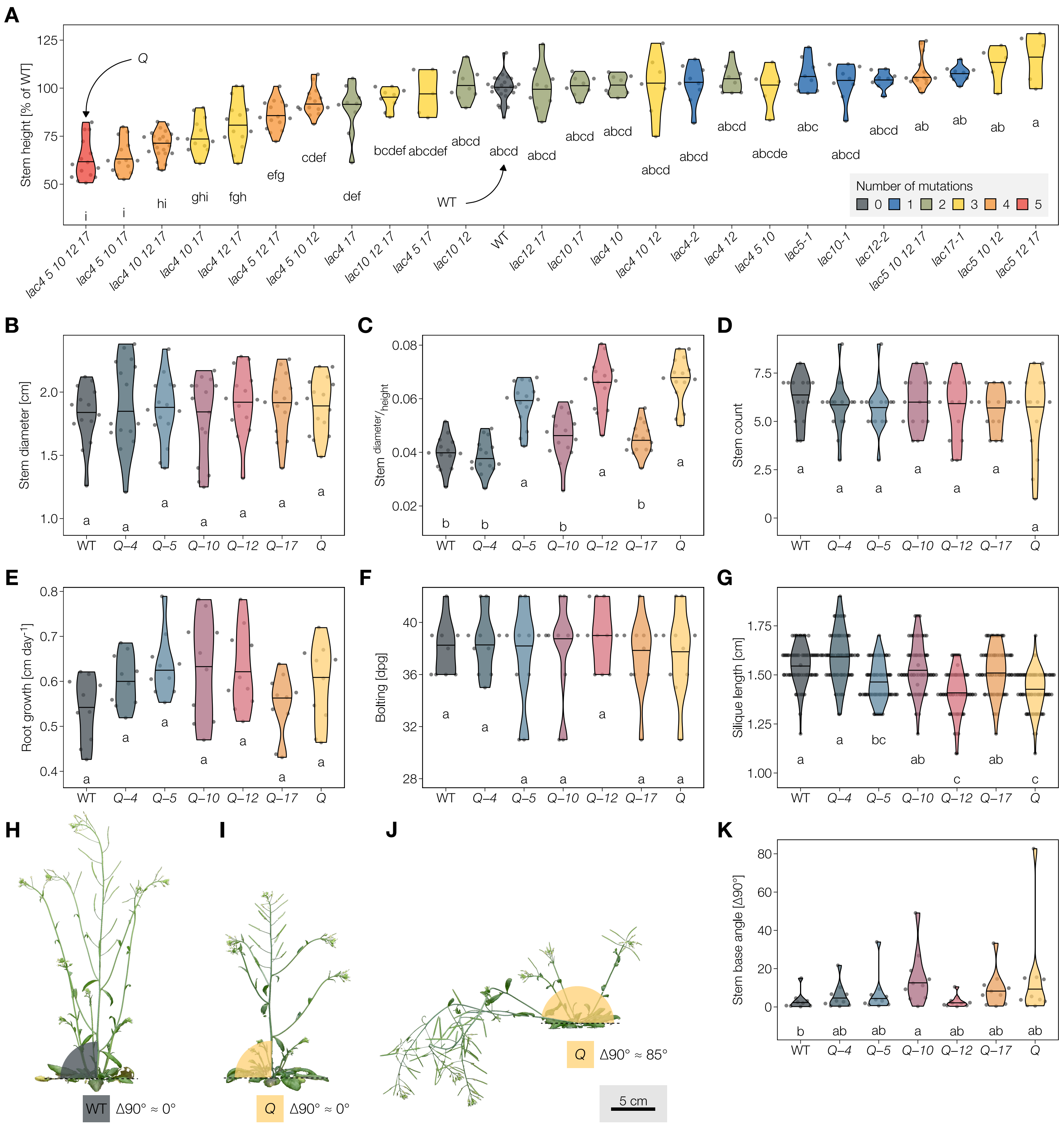

Supplemental Figure S2 | Phenotypic characterization of higher-order *lac* mutants. (Supports Figure 2).
A, Stem height at harvesting of the WT and *lac* single, double, triple, quadruple and quintuple mutants (color-coded), sorted by median plant height. n = 5–24 individual plants per genotype. Stem height was expressed as percentage of the WT to minimize noise from differences between growth instances. **B**, Basal stem diameter of higher-order *lac* mutants at harvesting; n = 10 individual plants per genotype from 2 independent growth instances. **C**, Stem width-to-height ratio of higher-order *lac* mutants; n = 10 individual plants per genotype from 2 independent growth instances. **D**, Number of stems in higher-order *lac* mutants at harvesting; n = 10 individual plants per genotype from 2 independent growth instances. **E**, Early root growth rate between 2 and 7 d past germination; n = 10 individual seedlings per genotype from 2 plates per genotype. **F**, Bolting time (defined as reaching a main stem height > 5 cm) of higher-order *lac* mutants; n = 10 individual plants per genotype from 2 independent growth instances. **G**, Length of mature siliques from the main stem at time of harvest; 5 mature siliques were measured for each of n = 15 individual plants per genotype from 3 independent growth instances. **H**, Typical upright stature of WT plants with Δ90° close to 0° at 45 days past germination. **I**, Typical upright stature of higher-order *lac* mutants with Δ90° close to 0° at 45 days past germination. **J**, Lodging of higher-order *lac* mutants with Δ90° close to 85° at 45 days past germination. **K**, Lodging of higher-order *lac* mutants at 45 days past germination using Δ90° measurements of at the stem base; n = 10 individual plants per genotype from 2 independent growth instances. Different letters indicate statistically significant differences between genotypes according to a Tukey-HSD test (per panel; α = 0.05).

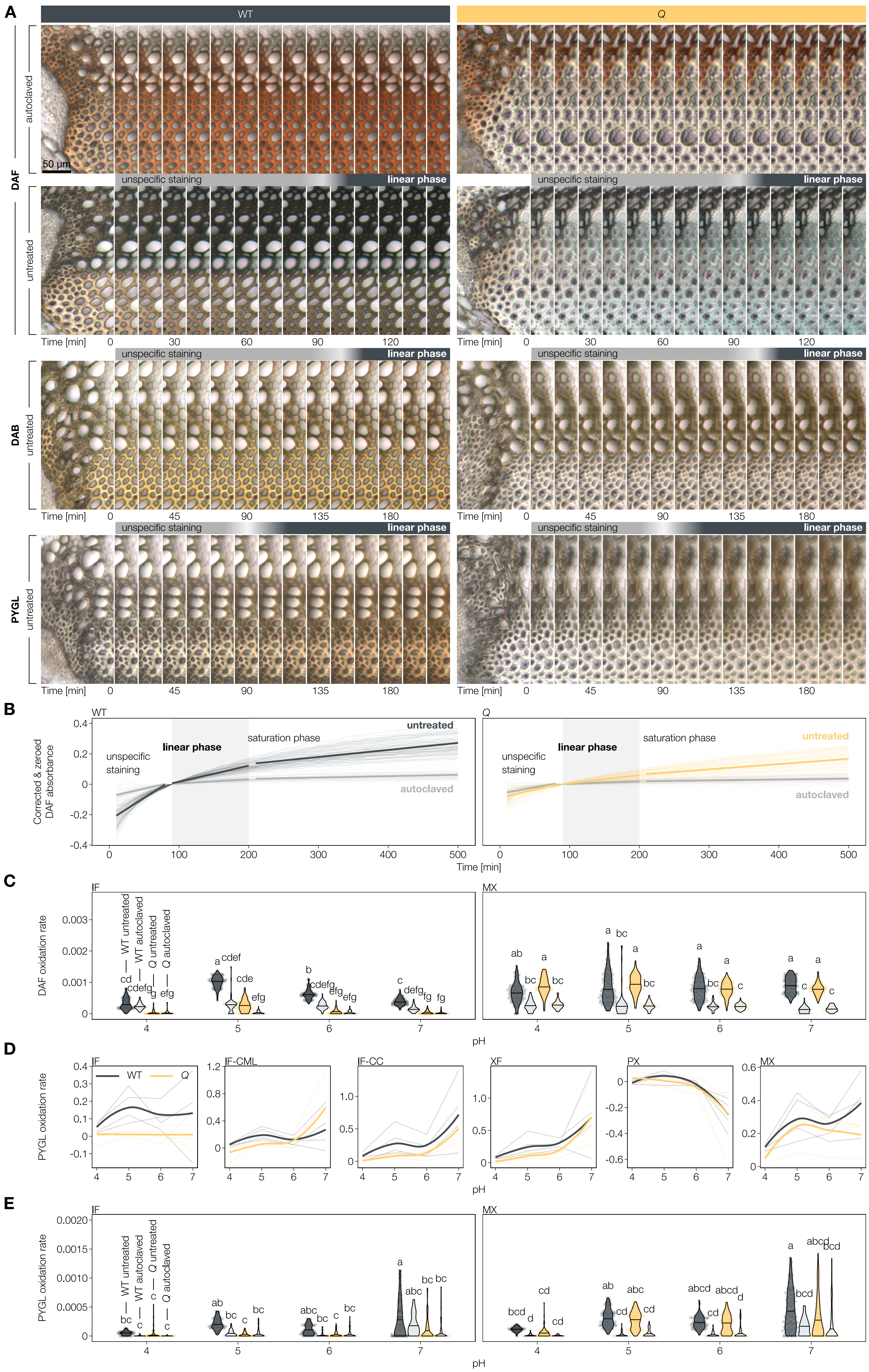
A
WT
Q
DAF
autoclaved
untreated
50 µm
unspecific staining
linear phase
Time [min] 0 30 60 90 120
DAB
untreated
Time [min] 0 45 90 135 180
PYGL
untreated
B
WT
Q
Corrected & zeroed DAF absorbance
unspecific staining
linear phase
saturation phase
untreated
autoclaved
Time [min]
C
IF
MX
DAF oxidation rate
WT untreated
WT autoclaved
Q untreated
Q autoclaved
pH
D
IF
IF-CML
IF-CC
XF
PX
MX
WT
Q
PYGL oxidation rate
pH
E
IF
MX
PYGL oxidation rate
pH

Supplemental Figure S3 | Real-time imaging of in situ LAC activity in extractive-free cross-sections. (Supports Figure 3).
A, Real-time sequential image montage of autoclaved and untreated WT and *Q* sections incubated with 2,7-diaminofluorene (DAF), as well as untreated sections incubated with DAB and pyrogallol (PYGL), all at pH 5. **B**, Relative substrate oxidation rates in cell walls of XFs measured during real-time imaging. Thin lines show 100 measured cell walls from 5 individual plants per genotype and condition. Thick lines show linear regressions of the initial phase of unspecific cell wall staining, the linear phase of active substrate oxidation, and the saturation phase when activity levels off. **C**, Relative DAF oxidation rates in the IF and MX of autoclaved and untreated WT and *Q* sections; *n* = 5 individual plants for untreated sections and *n* = 3 individual plants for autoclaved sections. **D**, Optimal pH for activity towards the phenolic LAC substrate pyrogallol, showing an additional activity peak at pH 7 for multiple cell types. Activity in autoclaved sections was subtracted to show only LAC-mediated oxidation. The activity rate drops into the negative when the apparent oxidation rate is lower than that of the unlignified phloem used for background correction. Thin lines represent four individual plants, thick lines are the overall average by local regression (LOESS). **E**, Relative PYGL oxidation rates in the IF and MX of autoclaved and untreated WT and *Q* sections; *n* = 4 individual plants for untreated sections and *n* = 3 individual plants for autoclaved sections. Different letters indicate statistically significant differences between genotypes according to a Tukey-HSD test (per panel; α = 0.05).

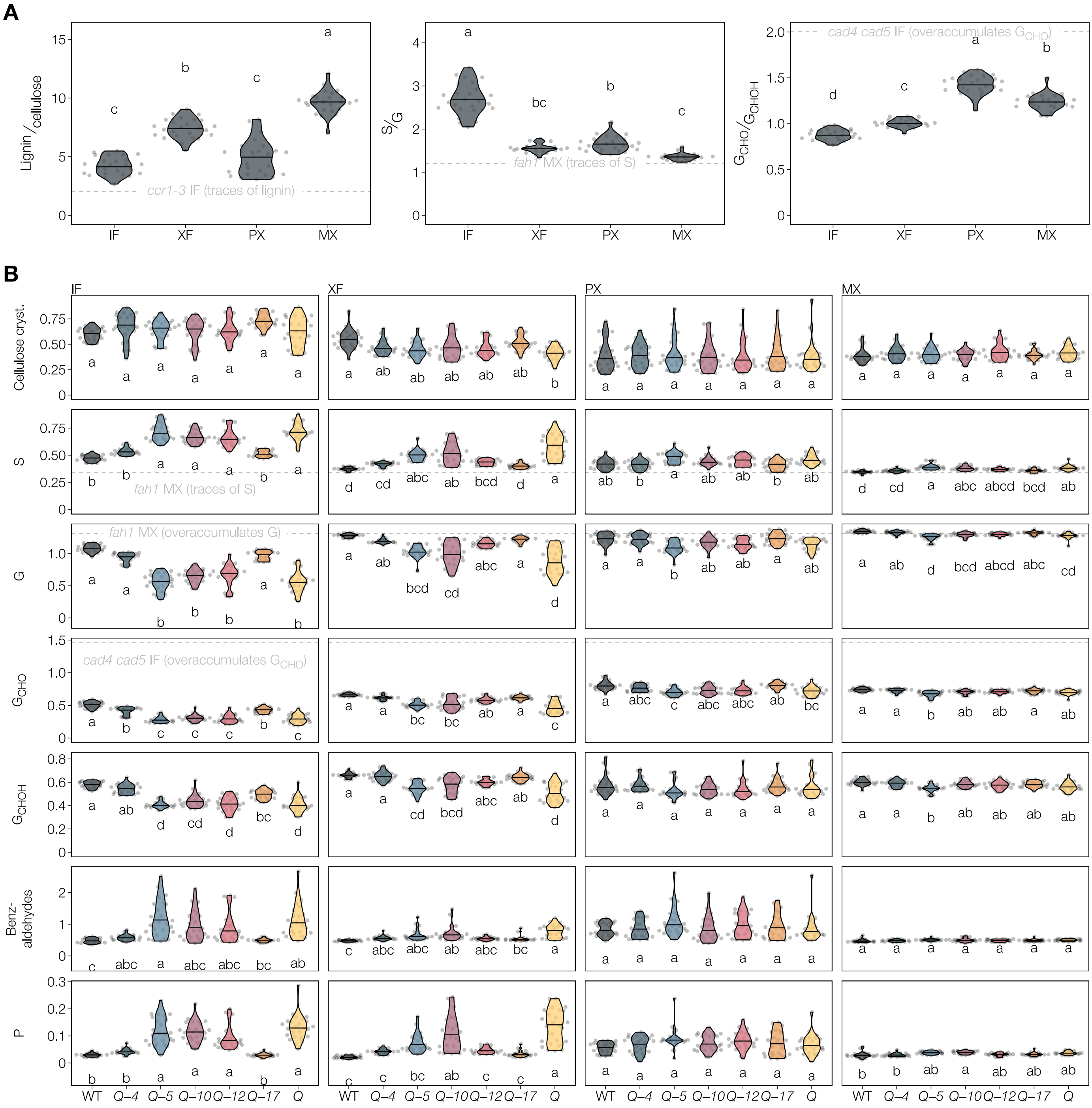

Supplemental Figure S4 | Lignin chemistry measured by Raman microspectroscopy. (Supports Figure 4).
A, Comparison of lignin levels and composition between different cell types and morphotypes in WT plants. **B**, Cell wall characteristics of WT and higher-order *lac* mutants. Different lignin constituents are expressed relative to total lignin amounts. References from well-characterized phenylpropanoid loss-of-function mutants are indicated by dashed gray lines to ease interpretation. Cellulose crystallinity was estimated as the ratio of the 378 and 1095 cm^{-1} bands. Different letters indicate statistically significant differences between genotypes according to a Tukey-HSD test (per panel; $\alpha = 0.05$); spectra of 5 individual cells were measured for each cell type in each of $n = 5$ individual plants per genotype from 2 independent growth instances.

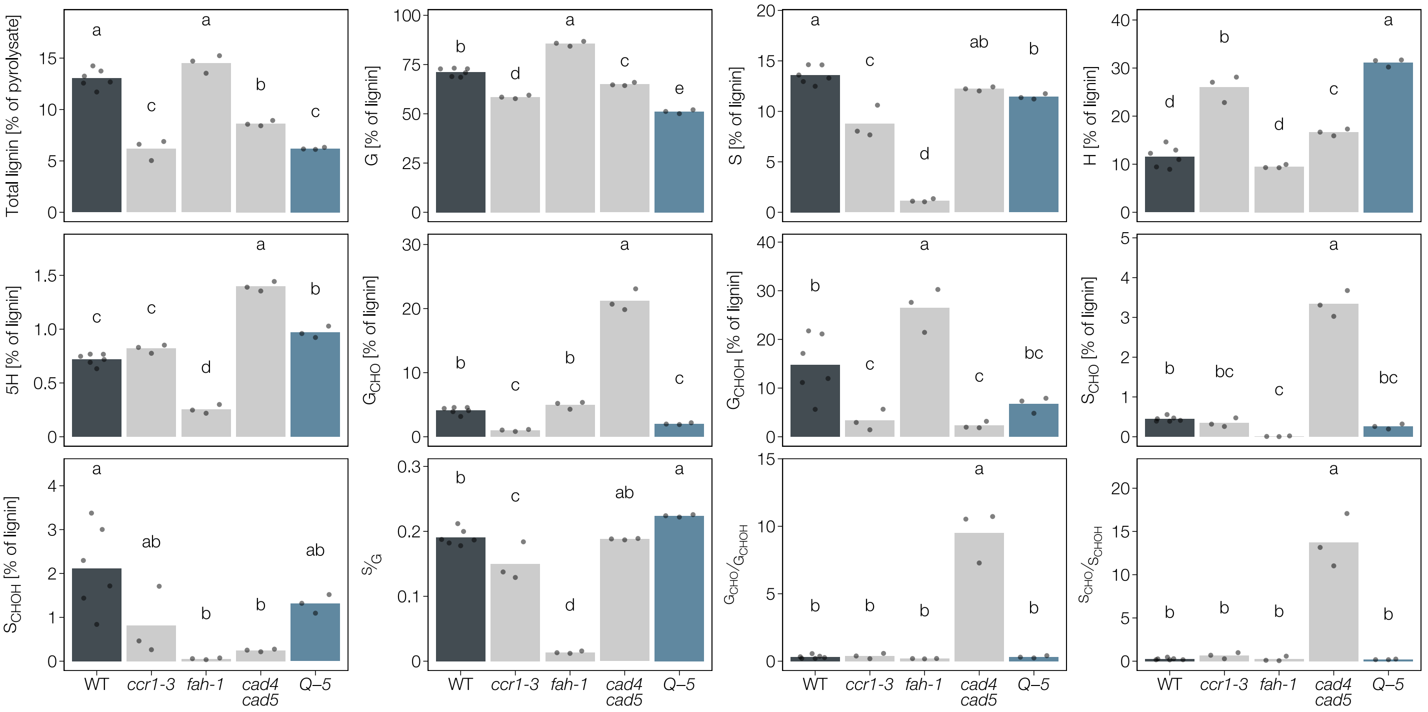

Supplemental Figure S5 | Validation of the in situ quantitative chemical imaging capacity of Raman microspectroscopy compared to pyrolysis/GC-MS. (Supports Figures 5 & 6).

Biochemical analyses of lignin levels and composition in whole ground stems using pyrolysis/GC-MS between WT and Q–5 (*Q* mutant with functional *LAC5*) as well as the well-characterized reference lignin mutants *ccr1-3* (Mir Derikvand et al. 2008) with reduced lignin levels, *fah1* (Meyer et al. 1998) with reduced S residues and *cad4 cad5* (Sibout et al. 2005) with increased G_{CHO}. Lignin chemistry and levels are presented as summed percentage of the total pyrogram area where each pyrolysate was identified by its m/z profiles; note however that no correction for differences in response factor were made (H residue pyrolysates have a ~five-fold higher relative response factor than G_{CHO} and G_{CHOH}, and a ~eight-fold higher relative response factor than S_{CHO} and S_{CHOH}; van Erven et al. 2017).

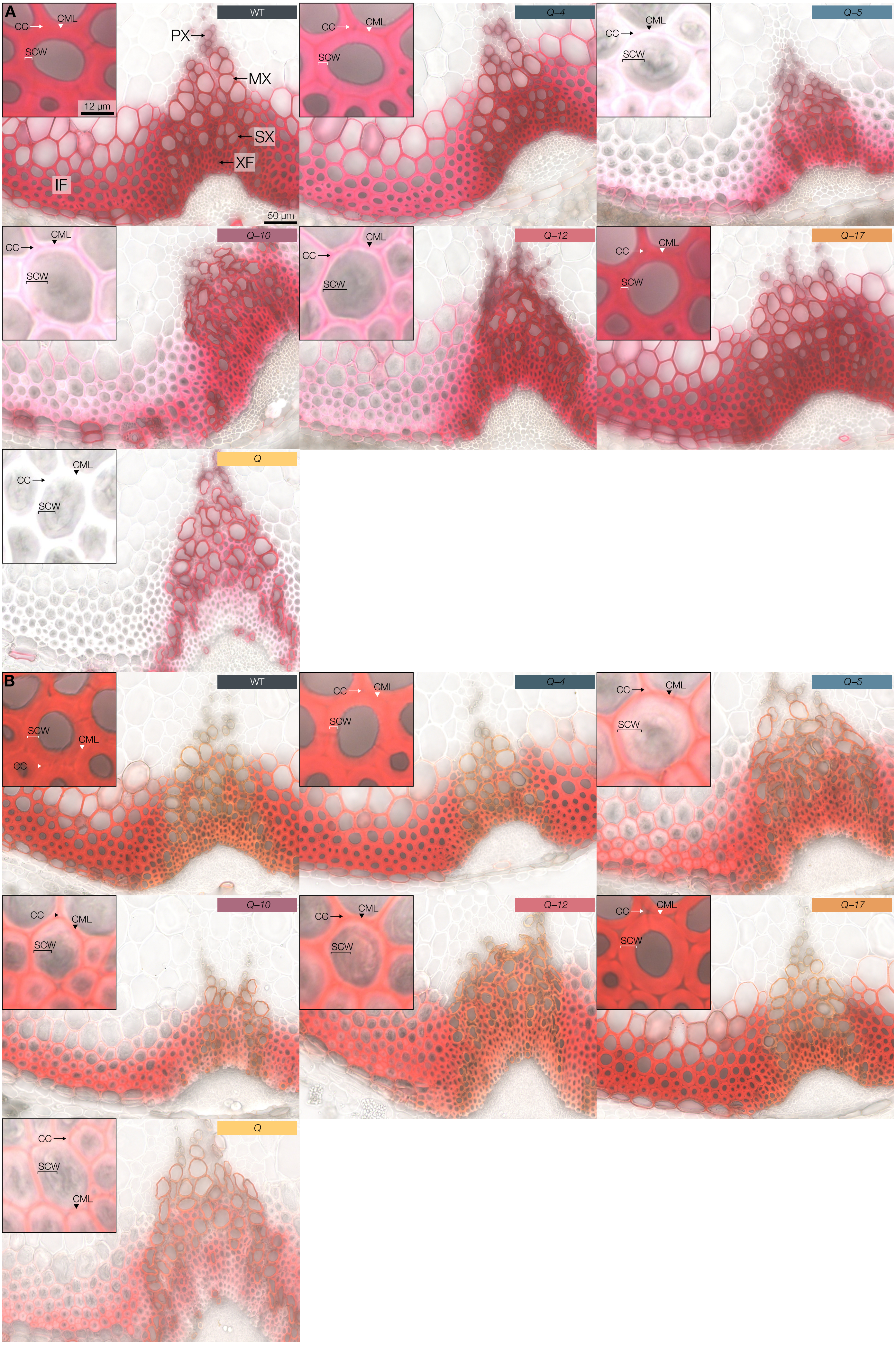
A
WT
CC
CML
SCW
12 µm
PX
MX
SX
XF
IF
50 µm
Q–4
Q–5
Q–10
Q–12
Q–17
Q
B
WT
Q–4
Q–5
Q–10
Q–12
Q–17
Q

Supplemental Figure S6 | Histochemistry of higher-order *lac* mutants. (Supports Figure 5).
A, Enlarged images of Wiesner-stained typical WT and higher-order *lac* mutants with indications of interfascicular fibers (IF), xylary fibers (XF), secondary xylem TEs (SX), primary metaxylem TEs (MX) and primary protoxylem TEs (PX) as well as the different cell wall layers of IFs with compound middle lamella (CML), cell corner (CC) and secondary cell wall (SCW). Note that unstained cross-sections show no staining. **B**, Enlarged images of Mäule-stained typical WT and higher-order *lac* mutants. Note that unstained cross-sections show no staining.

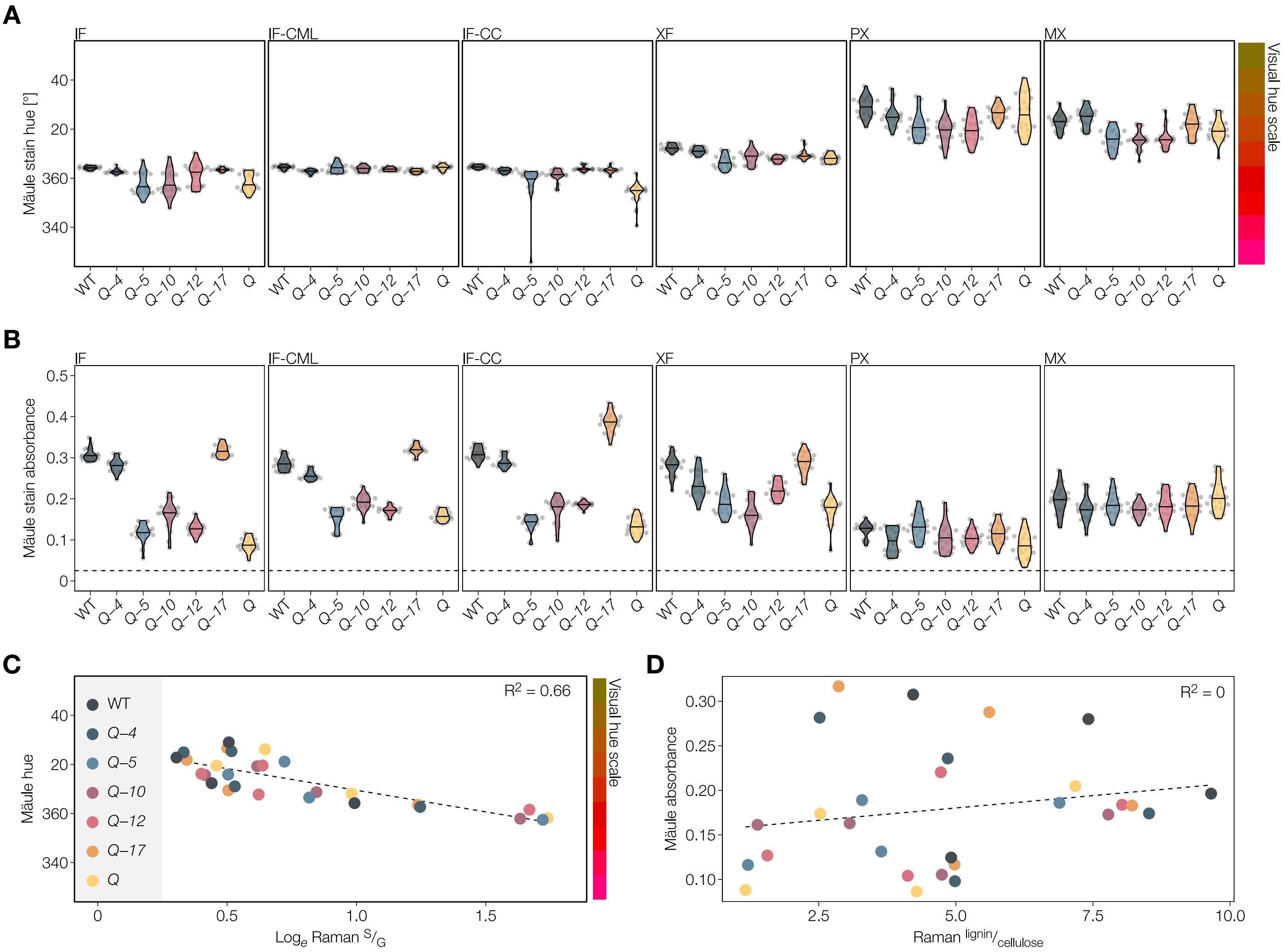

Supplemental Figure S7 | Mäule staining cell walls of higher-order *lac* mutants. (Supports Figure 5).
A, Hue change in higher-order *lac* mutants stained with Mäule test; 20 measurements per cell type and genotype from *n* = 1 stained section. **B**, Intensity change in higher-order *lac* mutants stained with Mäule test; 20 measurements per cell type and genotype from *n* = 1 stained section. **C**, Linear regression between Mäule-stained hue and measurements of lignin S/G using Raman (see Supplemental Table S5 for the used Raman bands). **D**, Linear regression between Mäule absorbance and measurements of lignin/cellulose content using Raman. Note that the quantitative analysis of the Mäule to determine lignin levels and S/G chemistry has not been validated and the present results are only an indication on possible semi-quantitative capacities of the Mäule test for determining S/G chemistry.

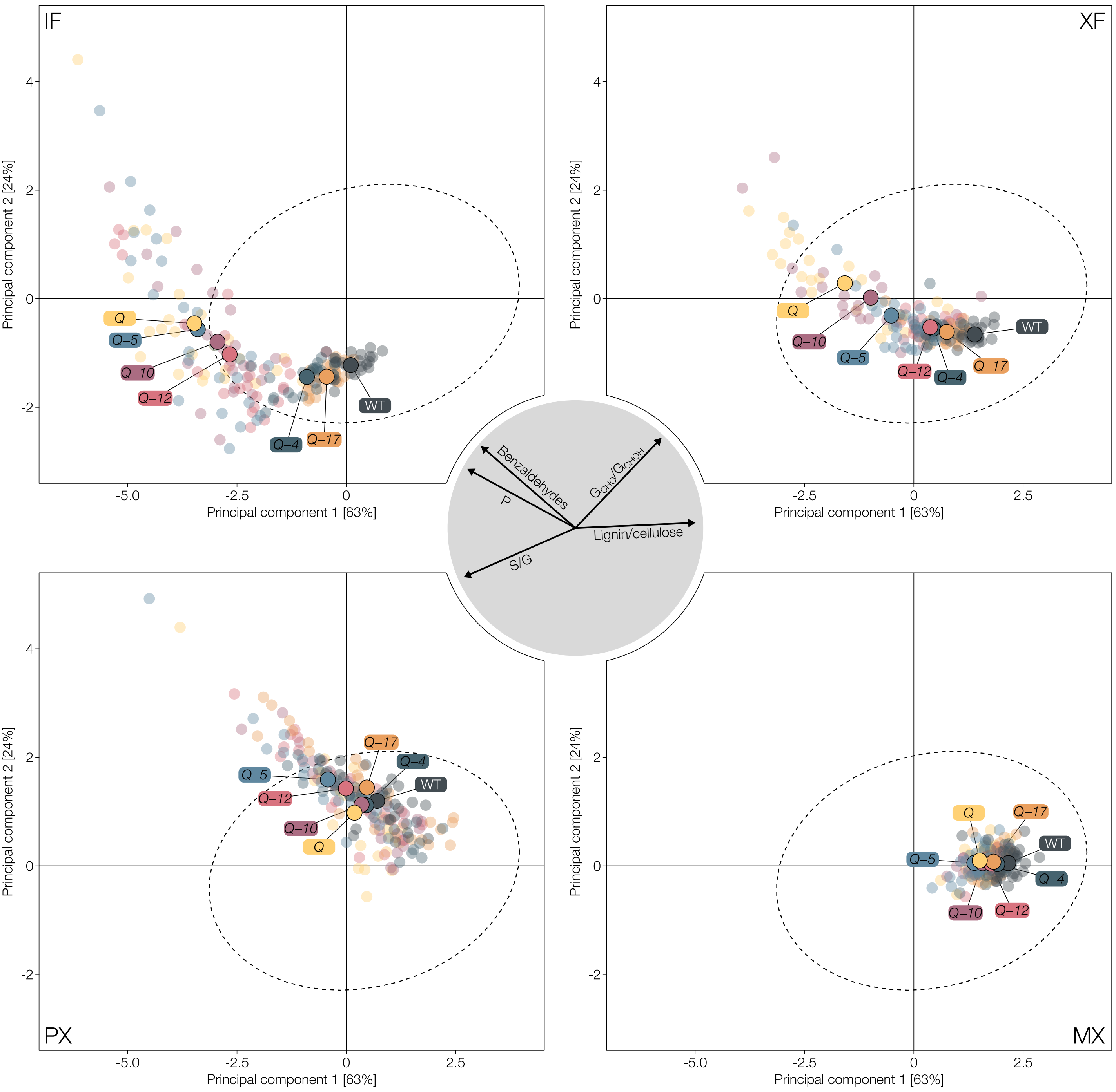

Supplemental Figure S8 | Multivariate analysis of the cell type-dependent roles of different LAC paralogs. (Supports Figure 4).
Principal component analysis (PCA) was performed to integrally compare the influence of each genotype on the cell wall lignin chemistry between cell types (fibers vs. TEs) and morphotypes (fibers: xylary vs. interfascicular; TEs: metaxylem vs. protoxylem). All four cell/morphotypes were included in the same PCA but are plotted separately to highlight the cell type-dependent effects in the higher-order *lac* mutants. To ease the reading of the Figure, relative vectorial loadings of the different lignin chemical parameters contributing to each principal component (PC) are indicated in the center with a gray background. Small transparent circles represent independent measurements (n = 50 per genotype and cell/morphotype) and larger non-transparent circles represent the mean value for each genotype. The 95% confidence ellipse is indicated.

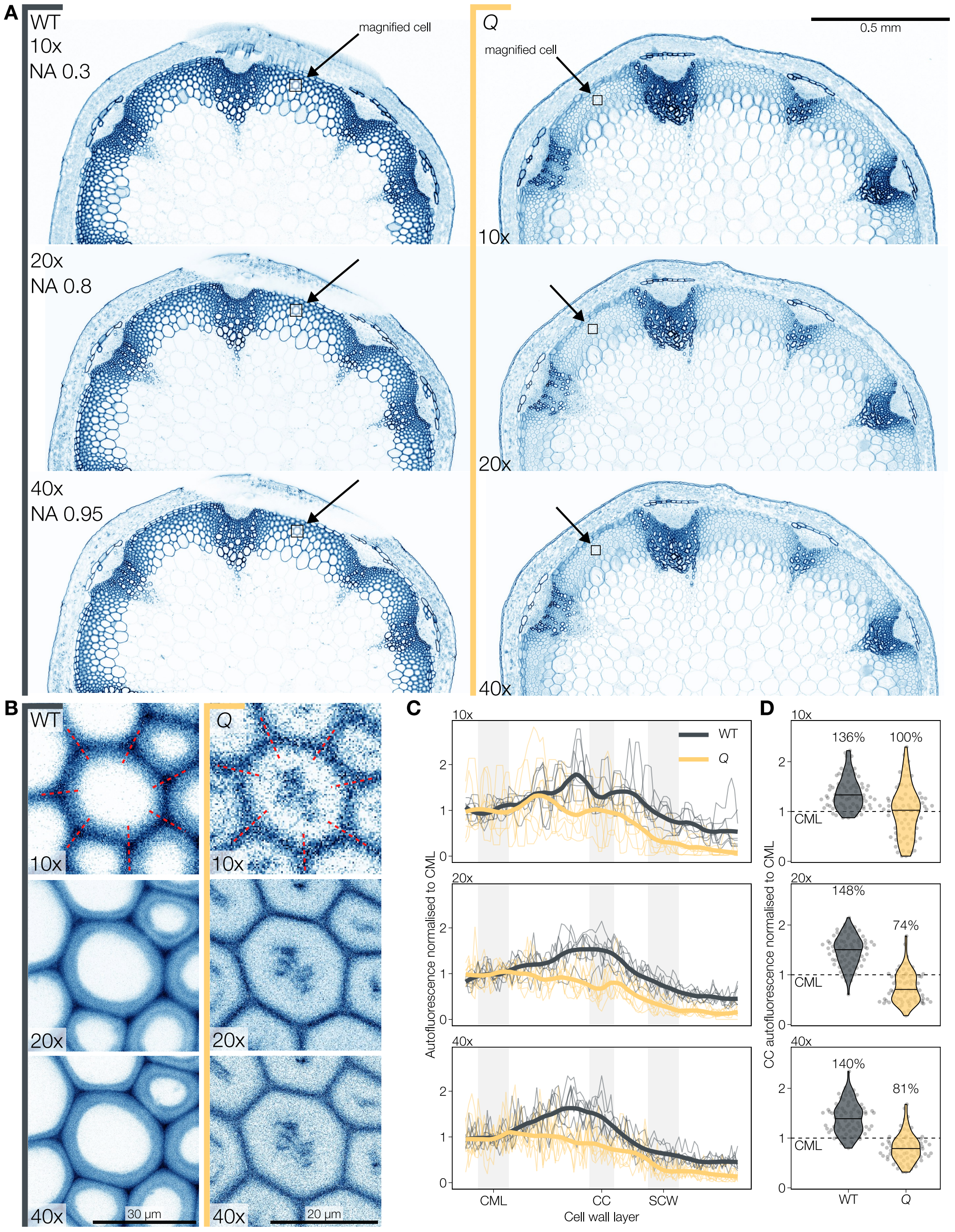

Supplemental Figure S9 | Imaging set-up necessary to measure differences in lignin autofluorescence between cell wall layers. (Supports Figure 6). **A**, Cross-sections of WT and *Q* plants acquired using different objectives differing in magnification and numerical aperture (NA). **B**, Close-up of boxes indicated by arrows in panel A of the tricellular junction of interfascicular fibers in WT and *Q* p lants. Red dotted lines indicate where lines profiles were measured. **C**, Relative autofluorescence profiles normalized to the compound middle lamella (CML) in WT and *Q* plants using different objectives. Thin lines correspond to individual measurements and thick lines are the averages for each genotype. Portions of the line profile easily assignable to distinct cell wall layers are shaded in gray and labeled. Note that the 10x profiles are the noisiest. **D**, Comparison of the relative autofluorescence of cell corners to CML in WT and *Q* plants with different objectives. Note that the 20x objective suffices to properly estimate difference in cell wall lignin autofluorescence across cell wall layers. Additionally, the higher signal/noise ratio of the 20x objective when compared to the 40x objective allows for a more reliable quantification between cell wall layers in the weakly fluorescent higher order *lac* mutants.

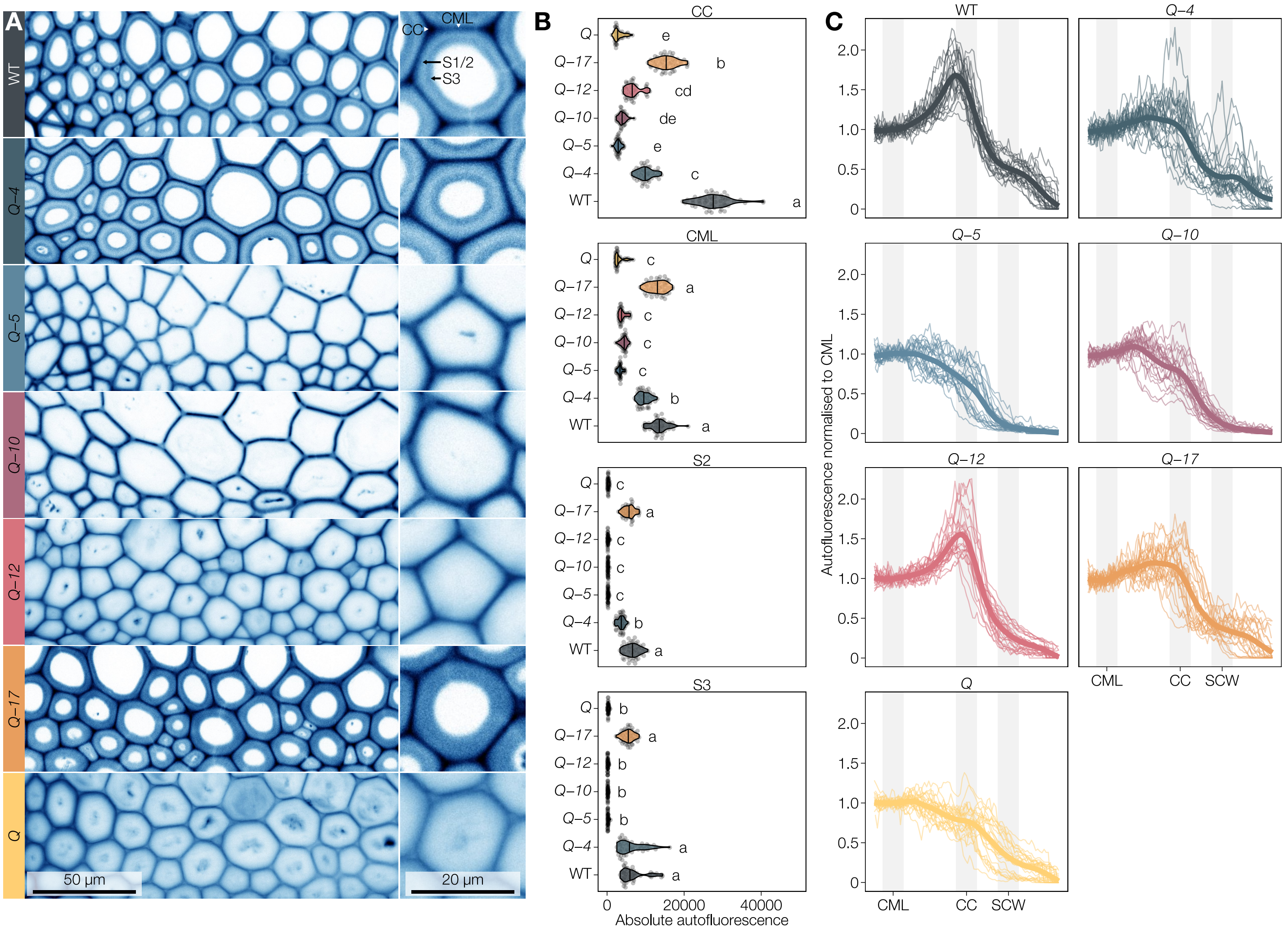

Supplemental Figure S10 | Cell wall layer-specific changes in lignin autofluorescence. (Supports Figure 6).
A, Lignin autofluorescence in IFs of WT and higher-order *lac* mutant plants. Image contrast has been adjusted for each genotype for visibility. **B**, Absolute lignin autofluorescence in CC, CML, S1/S2 and S3 layers of IFs in WT and higher-order *lac* mutants. The points represent 10 measurements per cell wall layer and genotype from each of n = 5 individual plants per genotype from 2 independent growth instances. Different letters indicate statistically significant differences between genotypes according to a Tukey-HSD test (per panel; $\alpha = 0.05$). **C**, Autofluorescence intensity line profiles through CML, CC and SCW. Each line is normalized to the average of its CML. Thin lines are individual profiles from n = 5 independent plants, the thick line is an average by local regression (LOESS).

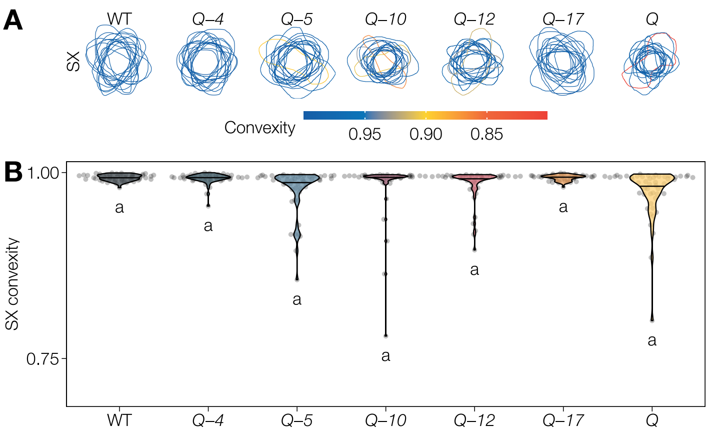

Supplemental Figure S11 | Perimeters and degree of inwards collapse of SX TEs in the different higher-order *lac* mutants. (Supports Figure 7). **A**, Drawn outlines of the cell walls of fifteen randomly sampled TEs per genotype. **B**, Degree of SX collapse in the different *lac* mutants and WT plants. Ten TEs in each of n = 5 individual plants per genotype were measured. Different letters indicate statistically significant differences between genotypes according to a Tukey-HSD test (per panel; α = 0.05).

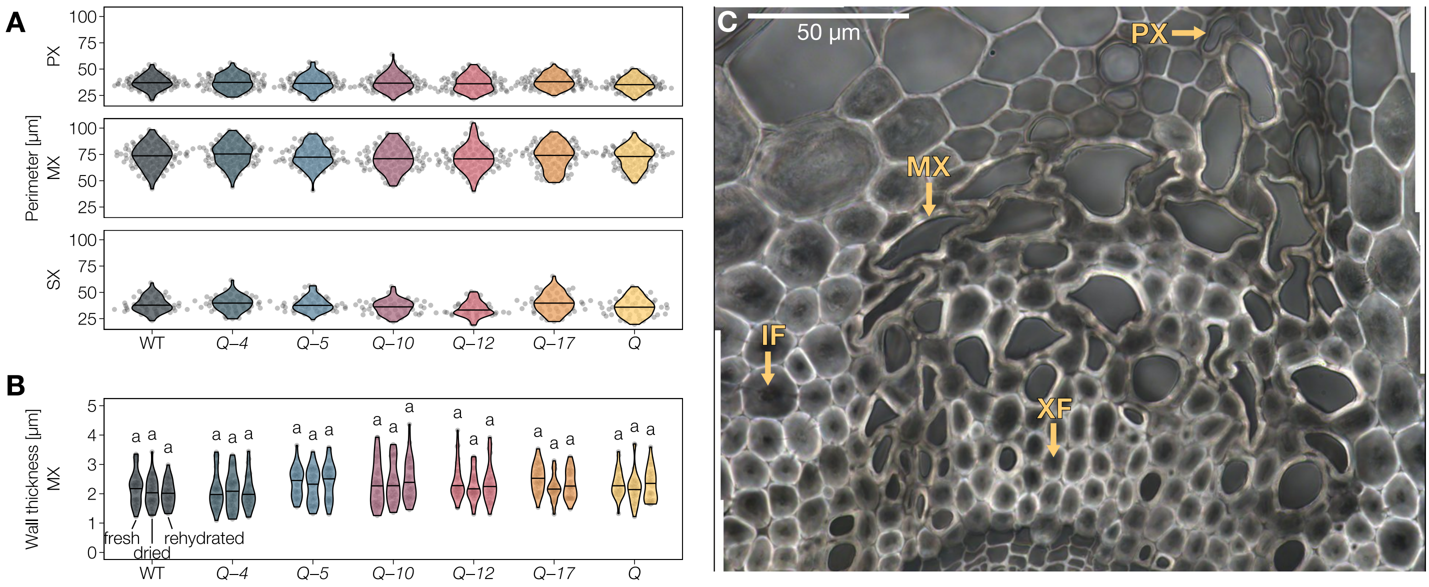

Supplemental Figure S12 | Cell wall properties of TEs and fibers. (Supports Figures 7 and 8). **A**, Perimeter lengths of MX and PX TEs are similar in WT and all tested mutants. Values are from the 20 TEs of each morphotype in each of n = 5 individual plants per genotype shown in Figure 7. **B**, TE cell wall thickness is unaffected by drying in higher-order *lac* mutants. Dots represent 10 TE cell walls from each of n = 3 individual plants per genotype in the three states. Cell walls were measured lumen to lumen in TE–TE and TE–parenchyma cell walls, explaining the overall variation in cell wall thickness. Different letters indicate statistically significant differences between genotypes and states according to a Tukey-HSD test (per panel; α = 0.05). **C**, Differential interference contrast (DIC) image of a *Q* vascular bundle, showing the swollen cell walls in both IFs and XFs as well as inwardly collapsed TEs.

References

Meyer, K., A. M. Shirley, J. C. Cusumano, D. A. Bell-Lelong and C. Chapple. 1998. Lignin monomer composition is determined by the expression of a cytochrome P450-dependent monooxygenase in *Arabidopsis*. *Proceedings of the National Academy of Sciences* 95 (12): 6619–6623. https://doi.org/10.1073/pnas.95.12.6619.

Mir Derikvand, M., J. B. Sierra, K. Ruel, B. Pollet, C.-T. Do, J. Thévenin, D. Buffard, L. Jouanin and C. Lapierre. 2008. Redirection of the phenylpropanoid pathway to feruloyl malate in *Arabidopsis* mutants deficient for cinnamoyl-CoA reductase 1. *Planta* 227, no. 5 (2008): 943–956. https://doi.org/10.1007/s00425-007-0669-x.

Sibout, R., A. Eudes, G. Mouille, B. Pollet, C. Lapierre, L. Jouanin and A. Séguin. 2005. *CINNAMYL ALCOHOL DEHYDROGENASE-C* and *-D* are the primary genes involved in lignin biosynthesis in the floral stem of Arabidopsis. *The Plant cell* 17, no. 7 (2005): 2059–76. https://doi.org/10.1105/tpc.105.030767.

Van Erven, G., R. de Visser, D. W. H. Merkx, W. Strolenberg, P. de Gijsel, H. Gruppen and M. A. Kabel. 2017. Quantification of Lignin and Its Structural Features in Plant Biomass Using 13C Lignin as Internal Standard for Pyrolysis-GC-SIM-MS. *Analytical Chemistry* 89, no. 20 (2017): 10907–10916. https://doi.org/10.1021/acs.analchem.7b02632.

Supplemental Table S1. Polymorphisms of the used *lac* mutant plants and the primers used to genotype them. Band sizes estimated from gel electrophoresis. KO, knockout; KD, knockdown.

Allele	Locus	Polymorphism	Insertion	Effect of insertion	WT allele primers (5'→3')	Mutant allele primers (5'→3')
lac4-2	AT2G38080	GK-720G02-025278	exon 3	KO (Berthet et al. 2011; this study)	FW: TGGTAACTTTGGACGATCAGG<br>RV: AGTAATGAACAGTTGCGGTGG<br>band size: ~1 kb	FW: ATATTGACCATCATACTCATTGC[a]<br>RV: AGTAATGAACAGTTGCGGTGG<br>band size: ~1.1 kb
lac5-1	AT2G40370	SALK_063466	exon 5	ΔCu (Cai et al. 2006; this study)	FW: ACTTCTCGTCTTTCCTCCTGC<br>RV: CTTGGAAGAGCAAATGAAACG<br>band size: ~1 kb	FW: ACTTCTCGTCTTTCCTCCTGC<br>RV: GCGTGGACCGCTTGCTGCAACT[b]<br>band size: ~800 bp
lac10-1	AT5G01190	SALK_017722	exon 5	ΔCu (Cai et al. 2006; this study)	FW: TCAATTCCAAGACATATCCGG<br>RV: CTATGGATCAATCAGAAGTCCG<br>band size: ~1 kb	FW: GCGTGGACCGCTTGCTGCAACT[b]<br>RV: CTATGGATCAATCAGAAGTCCG<br>band size: ~750 bp
lac11-1	AT5G03260	SALK_063746	exon 5	KO (Cai et al. 2006)	FW: ATTTCAATGTGACCGGACAACG<br>RV: TAAGTCTTGTCCCCGTTGATG<br>band size: ~1.2 kb	FW: GCGTGGACCGCTTGCTGCAACT[b]<br>RV: TAAGTCTTGTCCCCGTTGATG<br>band size: ~500 bp
lac12-2	AT5G05390	SALK_125379	exon 1	ΔSP (this study)	FW: TTTCTGCCAACATTTTTGAGG<br>RV: AGGGAAAGGAAAAGAGGAACC<br>band size: ~1.1 kb	FW: TTTCTGCCAACATTTTTGAGG<br>RV: GCGTGGACCGCTTGCTGCAACT[b]<br>band size: ~500 bp
lac17-1	AT5G60020	SALK_016748	promoter	KD (Cai et al. 2006; this study)	FW: TCGAAGAGGGTCAAAGAGTTT<br>RV: TCTTAGCCATGAAATGTGAGC<br>band size: ~900 bp	FW: GCGTGGACCGCTTGCTGCAACT[b]<br>RV: TCTTAGCCATGAAATGTGAGC<br>band size: ~900 bp

[KO] knockout
[KD] knockdown
[ΔCu] truncated transcript missing copper binding sites necessary for enzymatic activity
[ΔSP] truncated transcript missing the signal peptide necessary for cell wall localisation
[a] Gabi-Kat LB primer
[b] SALK LB primer

Supplemental Table S2. Summary of the names and genotypes used to designate higher-order *lac* mutants.

Mutant	*LAC4*	*LAC5*	*LAC10*	*LAC12*	*LAC17*
Q	*lac4-2*<br>*lac4-2*	*lac5-1*<br>*lac5-1*	*lac10-1*<br>*lac10-1*	*lac12-2*<br>*lac12-2*	*lac17-1*<br>*lac17-1*
Q–4	*LAC4*<br>*LAC4*	*lac5-1*<br>*lac5-1*	*lac10-1*<br>*lac10-1*	*lac12-2*<br>*lac12-2*	*lac17-1*<br>*lac17-1*
Q–5	*lac4-2*<br>*lac4-2*	*LAC5*<br>*LAC5*	*lac10-1*<br>*lac10-1*	*lac12-2*<br>*lac12-2*	*lac17-1*<br>*lac17-1*
Q–10	*lac4-2*<br>*lac4-2*	*lac5-1*<br>*lac5-1*	*LAC10*<br>*LAC10*	*lac12-2*<br>*lac12-2*	*lac17-1*<br>*lac17-1*
Q–12	*lac4-2*<br>*lac4-2*	*lac5-1*<br>*lac5-1*	*lac10-1*<br>*lac10-1*	*LAC12*<br>*LAC12*	*lac17-1*<br>*lac17-1*
Q–17	*lac4-2*<br>*lac4-2*	*lac5-1*<br>*lac5-1*	*lac10-1*<br>*lac10-1*	*lac12-2*<br>*lac12-2*	*LAC17*<br>*LAC17*

Supplemental Table S3. Primers used for RT-qPCR analyses.

Gene	Locus	Note	Primer	Sequence
EF1a	At5g60390		FW	TCCAGCTAAGGGTGCC
			RV	GGTGGGTACTCGGAGA
UBI	At4g36800		FW	CTGTTCACGGAACCCAATTC
			RV	GGAAAAAGGTCTGACCGACA
18S rRNA	At3g41768		FW	TGCTACTCGGATAACCGTAG
			RV	TGTCACTACCTCCCCGTGTC
CesA8	At4g18780		FW	CAAAACCGATACGACTTCCC
			RV	TAACTTGCGGAACCCGCCAA
CesA7	At5g17420		FW	CTCAAAGGTCTGATGGGTAG
			RV	TACTCGCTCACAATAAACCC
XCP1	At4g35350		FW	CTCTCCCCATCTTGGTCCCC
			RV	GTCAGTGTGGCTATTGAGGC
XCP1	At4g35350	for end-point RT-PCR	FW	TGGCTGCAATGGAGGTCTCA
		for end-point RT-PCR	RV	GAGTCCCTCTGGTTTACCAG
XCP2	At1g20850		FW	TTGACCACGGTGTGGCTGCG
			RV	GTTGTTACAAGACTTCCGTT
CAD4	At3g19450		FW	GGGCTTACGTCGACGATTGA
			RV	TCGTTGGACAAACAAGCCCG
CAD5	At4g34230		FW	GGGAGCTTCATAGGGAGCA
			RV	GTTTAAATCGAGTATTCGAG
CCR1	At1g15950		FW	AAAGCCTCTACGACACAGTC
			RV	GGCCAAAGAACTGAGTGTAC
F5H1	At4g36220		FW	CCTCGATGAGGGCAACTTCA
			RV	GCGCTTGACTTAGCCGTGGC
LAC4	At2g38080	downstream of T-DNA	FW	TGGTAGATCCGGTTGAGAGG
		downstream of T-DNA	RV	TAGTCTTCTTGGACGTGGCG
LAC5	At2g40370	downstream of T-DNA	FW	AGCCTCCTCATGATTTGCCC
		downstream of T-DNA	RV	GCCAACTCAGGAGTATGACCC
LAC10	At5g01190	downstream of T-DNA	FW	CCTCCAAGTGATCTTCCCAA
		downstream of T-DNA	RV	CAACCTTTCATTAAAGCGTAAC
		upstream of T-DNA	FW	ATTAGCGGTAGAGAGCGGGA
		upstream of T-DNA	RV	GGGGGCGATTAGGATTGTGT
LAC12	At5g05390	downstream of T-DNA	FW	ATCGGAGAATCACCCGATTC
		downstream of T-DNA	RV	AGGTAGATCGTGAGGAGGAG
		upstream of T-DNA	FW	AAACGTTAATCTCAAATTTTTCGTA
		upstream of T-DNA	RV	CATTAAGTGTGTTTAAGCTTTGAG
LAC17	At5g60020	downstream of T-DNA	FW	TGGAAGTGCATACCAGTTGG
		downstream of T-DNA	RV	AACCGATAAATGGCCGAGG

Supplemental Table S4. Synthetic substrates used for activity assays in sections.

Name	Abbreviation	Supplier	Identifier	Stock concentration	Solvent	Working conc.
2,7-Diaminofluorene	DAF	Sigma-Aldrich	D17106	7 mM	24 mM HCl in water	70 µM
3,3’-Diaminobenzidine	DAB	Sigma-Aldrich	D8001	5 mM	12 mM HCl in water	500 µM
Pyrogallol	PYGL	Sigma-Aldrich	P0381	50 mM	Water	5 mM

Supplemental Table S5. Raman band intensities and intensity ratios used for cell wall characterization.

Target	Band	Reference	Note
Total lignin	1334 cm^{-1} + (1600 cm^{-1} / 2)	Blaschek et al. 2020	Weighted average to account for the differences in scattering intensity
Cellulose	378 cm^{-1}	Gorzsás 2017	Orientation-insensitive
S	1334 cm^{-1}	Blaschek et al. 2020	Residues with aromatic syringyl rings
G	1600 cm^{-1} or 1273 cm^{-1}	Blaschek et al. 2020	1273 cm^{-1} is for the S/G ratio because S also slightly scatters at 1600 cm^{-1}
G_{CHO}	1624 cm^{-1}	Blaschek et al. 2020	Only reliably quantifies polymer end-units; shifted to 1634 cm^{-1} in the *cad4 cad5* mutant
G_{CHOH}	1658 cm^{-1}	Blaschek et al. 2020	Quantifies total (internal and end-units) G_{CHOH} content
Benzaldehydes	1303 cm^{-1} + 785 cm^{-1} + 1297 cm^{-1} + 450 cm^{-1}	Ménard et al. 2022	Bands reflect O–4-bound vanillin, other vanillin, O–4-bound syringaldehyde and other syringaldehyde respectively
P	993 cm^{-1}	Ménard et al. 2022	Residues with aromatic unsubstituted phenyl rings
Cellulose crystallinity	378 cm^{-1} / 1095 cm^{-1}	Agarwal et al. 2010	
