# Supplementary material for: Different combinations of laccase paralogs nonredundantly control the amount and composition of lignin in specific cell types and cell wall layers in Arabidopsis
Source: Plant Cell. 2022 Nov 30;35(2):889–909. doi: 10.1093/plcell/koac344 (PMC9940878; doi:10.1093/plcell/koac344)
Supplement: koac344_Supplementary_Data [file tpc22.00433.PeerReview.pdf › tpc22.00433.PeerReview.pdf]

Different combinations of laccase paralogs nonredundantly control the amount and composition of lignin in specific cell types and cell wall layers in Arabidopsis

Leonard Blaschek, Emiko Murozuka, Henrik Serk, Delphine Ménard, Edouard Pesquet

Corresponding author: Edouard Pesquet (edouard.pesquet@su.se)

Review timeline:

**TPC2022-RA-00433**	Submission received:	May 3, 2022
	1st Decision:	June 3, 2022 *revision requested*
**TPC2022-RA-00433R1**	1st Revision received:	August 26, 2022
	2nd Decision:	September 26, 2022 *accept with minor revision*
**TPC2022-RA-00433R2**	2nd Revision received:	October 14, 2022
	3rd Decision:	October 27, 2022 *acceptance pending, sent to science editor*
	Final acceptance:	November 23, 2022

REPORT: (The report shows the major requests for revision and author responses. Minor comments for revision and miscellaneous correspondence are not included. The original format may not be reflected in this compilation, but the reviewer comments and author responses are not edited, except to correct minor typographical or spelling errors that could be a source of ambiguity.)

TPC2022-RA-00433 1st Editorial decision – *revision requested* June 3, 2022

We have received reviews of your manuscript entitled "Different combinations of laccase paralogs non-redundantly control the lignin amount and composition of specific cell types and cell wall layers in Arabidopsis." Thank you for submitting your best work to The Plant Cell. The editorial board agrees that the work you describe is substantive, falls within the scope of the journal, and may become acceptable for publication, pending revision and potential re-review.

Having said this, it will be important to thoroughly address all of the reviewer comments, and we ask you to pay particular attention to the following points in preparing your revision:

As some of the reviewers point out, there are some concerns about the heavy reliance on microscopy data in the current version of the manuscript, and their use to draw conclusions about lignin content in specific cell wall layers and overall content in the plants. Further corroboration of the existing results, through higher-resolution imaging and/or a non-microscopy-based quantification of lignin and/or cellulose content, would improve confidence in the conclusions drawn from the current results.

In addition, it will be important to explain in detail how quantitative results were obtained from the imaging data, so that experiments and analyses could be replicated precisely.

Third, placing the results of the present work in context and explaining exactly how they advance knowledge of lignification, especially how the different laccases under study here influence lignin deposition in different wall layers, both in a graphical format as part of a conclusion figure and in the discussion text, would aid in clarifying the novelty and impact of this work.

Fourth, a more detailed introduction to laccase enzyme function and a thorough discussion of current knowledge regarding how laccase activity is linked to specific lignification processes would be helpful to readers who are not specialists in this field.

Finally, the reviews point out that many sentences in the manuscript contain numerous acronyms; these should be minimized whenever possible to improve the clarity and readability of the manuscript.

We should stress that we are reluctant to see manuscripts undergoing multiple rounds of revision and would be unlikely to offer you more than one chance to satisfy the reviewers.

-- Reviewer comments:

TPC2022-RA-00433R1 1st Revision received August 26, 2022

Reviewer comments and **author responses**:

Reviewer #1:

In this manuscript, the authors go to painstaking effort to delineate the role of laccases in lignification in Arabidopsis. I say painstaking because first, they generated six multiple LAC mutants, a quintuple, plus five additional lines lacking only four of the five mutated in the quintuple mutant. These valuable genetic resources were then analyzed in a host of different ways, including whole plant growth measurements, in situ laccase activity assays, and microscopy using histochemical, UV, and Raman visualization (although I have to wonder whether some images are ultimately too small to represent the authors' findings in proper detail in the standard journal format... I had to download the original image files and review them at much higher magnification). All microscopic analyses are accompanied by detailed quantification and statistical analysis. Overall, I have little to criticize in the manuscript but I do have some concerns.

Point 1. First, the authors on several occasions state things like "our results indicate that different LAC paralog combinations control the ring structure proportions in these two different categories of cell types." (line 292) and "Here, we expand on the importance of distinct lignin chemistries by showing that specific LACs control lignin composition to ensure lignin function in structurally reinforcing TEs and regulating cell wall swelling in fibres (figures 6, 7)." I can't agree with this statement because it is another example of the overuse of the idea of "regulation" when what is really proved or meant is "is required for." When and where laccases are absent, lignification does not occur. That is clear. But if a laccase is present, or possibly even a laccase with a specific substrate affinity, that doesn't mean that it can dictate the availability of the cognate substrate. The laccase can only act upon the substrates that are available to it and where the laccase is localized. The laccases enable the deposition of specific lignin types, but they do not control it.

RESPONSE: We thank the reviewer for this comment on the terminology used to define the role of LACCASEs in lignin formation. As LACCASEs have been recently suggested to drive the metabolic gradient required for the transport and supply of lignin precursors to cell walls (Perkins et al., 2022), we added novel expression data to assess how strongly LACCASEs were associated to lignin forming conditions compared to phenylpropanoid biosynthesis genes (we used CCR1 and CAD4;5). To do so, we added expression profiles (determined using microarray as well as RT-qPCR) of LACCASE paralogs and phenylpropanoid biosynthesis genes during lignifying and non-lignifying condition using inducible cell cultures as well as performed co-regulation analyses with SCW biosynthesis genes and PCD autolysis genes (new figure 1). This new expression data showed that our six selected LACCASE paralogs, in contrast to phenylpropanoid genes, are exclusively expressed in lignifying conditions and tightly co-regulated with SCW and PCD. This result shows that LACCASEs are thus more associated with lignin formation than phenylpropanoid biosynthesis genes, which relate more to phenolic formation. This uncoupling between phenolic precursor and lignin is however not surprising as post-mortem lignification of TEs has been shown for several plant species using paracrine and even endocrine supply of precursors (Pesquet et al., 2010; Pesquet et al., 2013; Derbyshire et al., 2015; Blaschek et al., 2020). This new data was included in the paragraph entitled "LAC paralogs non-redundantly and synergistically affect stem growth" in our new Figure 1 as well as in the discussion. Albeit these new data reinforces the central role for LACCASEs in lignification, we nevertheless revised our wording from "control" to "are required" throughout the text.

Point 2. The text on the top of page 9 concerning Figure 2 is somewhat challenging because the authors do not make it clear what their basis of comparison is. In most experiments, one would make comparisons to wild type, but here they are making their comparison to Q, for example where they state "Q-12 showed increased levels of LAC activity specifically in CCs and to a lesser extent in the CML (figure 2B)." The basis of comparison needs to be explicitly and deliberately stated. I would also take some issue with the authors' use of the term "restored" here and elsewhere in the paper. I don't think that you can talk about activity being restored in Q-17 in comparison to Q because Q-17 did not originate from Q. If you had put a transgene into Q, yes, but these are independent mutants and Q-17 simply has not had all of its activity depleted by removal of the final LAC17. Perhaps phrasing such as "Q-17 continues to exhibit residual activity compared to Q" would be more appropriate.

RESPONSE: We thank the reviewer for this comment and have accordingly rephrased our text. We have now explained with which genotype the comparison were made as well as changed our uses of "restored" that was an overstatement to use instead "reached/attained/rose to WT levels".

Point 3. The authors are quite clear about their genetic materials when they state "To define the specific functions of each individual LAC, we analysed the quadruple homozygous loss-of-function mutants lac5;10;12;17 (Q-4), lac4;10;12;17 (Q-5), lac4;5;12;17 (Q-10), lac4;5;10;17 (Q-12) and lac4;5;10;12 (Q-17) in comparison to the Q mutant and wild-type (WT) plants." Nevertheless, their nomenclature system is a bit counterintuitive to me because at first, I confused the hyphen in, for example, Q-4, with something that was meant to represent Q minus 4. Instead, it could be argued that Q-4 means just the opposite. It is the Q mutant to which LAC4 has been added. Thus, I wonder whether it might make more sense to label name it Q+4. I don't want to annoy the authors with this (possibly silly) suggestion, I am just thinking about what might be the clearest way to represent the work to a novice reader, possibly a new graduate student entering the field. They need all the help and clarity they can get.

RESPONSE: We thank the reviewer for this comment on the naming system used for our different multiple stacked mutant but we respectfully do not agree - we used Q-4 to define the quintuple mutant without the mutated lac4 allele stacked, while we would have used the Q+4 to define the quintuple mutant complemented by LAC4 WT allele. Such naming is thus made to clearly distinguish between the level of stacked mutations using "-" (for minus) in reference to the quintuple rather the complementation with WT alleles using "+" (for plus) in reference to the quintuple. We have thus explained our naming system in the corresponding materials and methods.

Point 4. A bigger issue is that to some extent, the manuscript is written in code. Sentences like this are simply difficult to get through: "The magnitude of activity at the different pH was reduced in the Q mutant for SCW, CML and CC of IFs, slightly for XFs but unaltered for MX TEs (figure 2A)." Is it really necessary to use so many abbreviations throughout the manuscript? I think their use makes it much more difficult for the average reader.

RESPONSE: We thank the reviewer for this comment on the over-uses of abbreviations and have thus limited their uses throughout the text – our changes mostly affected reduced usage of CML and CC.

Point 5. Finally, my greatest concern about this manuscript is its impact. I want to be clear that I think that the experiments have been elegantly conducted, but for the most part, it turns out that the authors have elegantly refined, for most cell types, previous knowledge. The authors acknowledge previous work (Berthet et al., 2011; Zho et al., 2013) on LACs 4, 11, and 17 and extend it in greater detail (see Table 1), but one could argue that this level of detail would primarily be of interest only to specialists in the field.

RESPONSE: We thank the reviewer for this comment on the importance and novelty of our study relatively to previous published work on LACCASE and plant biomass. Lignin has mostly been considered as a bulk, and a recent study (Ménard et al., 2022, accepted last week in The Plant Cell) just revealed the importance of lignin chemistry for TE morphotype biomechanics and sap-conducting activity where each TE required a specific composition and temporal sequence of lignin residue to accumulate to fully resist extreme conditions. The novelty of the present study is to provide the mechanisms at the cell wall and cell type levels that enable this precise control of lignin chemistry. Our study with its cell wall and cell type/morphotype resolution demonstrated that specific LACCASE paralogs were responsible of the spatially restricted and chemically specific lignification not only affecting the aromatic substitution levels but also the terminal aliphatic function. This breaks away from the idea that LACCASEs are redundant, unspecific and randomly oxidize any phenolic compound that are supplied to them to make lignin. Our novel finding therefore revises previous assumptions on the assembly of lignin formation and provide an explanation of the cell wall layers and cell types/morphotype specific lignin chemistry that was convergently acquired several times during plant evolution. Moreover, our study reveals that lignification provides different functions to distinct cell types, such as increasing resistance to inward collapse for TEs and waterproofing cell walls of fibers, extending the role of lignin chemical diversity between cell types and its important evolutionary complexification in vascular plants. We thus accordingly clarified the novelty and significance of our study in the discussion (using track-change).

Reviewer #2:

A major outstanding question in the cell wall field is how plants regulate when, where, and what type of lignin is deposited in the cell wall, and the functional implications of this for plant growth and development. Blaschek et al. have performed extensive and rigorous microscopy analyses which provide insight into how lignin composition and amount in different tissues and cell wall layers could be regulated by particular LACCASE isoforms in the cell wall. Using a series of quadruple mutants and a quintuple lac mutant, they show that different isoforms play specific roles in determining lignin deposition. The manuscript is well written and contributes evidence to a growing number of papers that demonstrate the importance of specific oxidative enzymes in controlling lignin deposition in a spatio-temporally regulated manner. However, there are several technical aspects of the paper that I think should be addressed, as outlined below.

Point 1. The manuscript is focused on determining lignin composition in certain regions; however, the data would benefit by adding in a non-microscopy method of quantifying total lignin content in the stems of the different mutants (e.g. Klason lignin) and changes to lignin composition (e.g. thioacidolysis for S:G ratio). This could provide additional evidence for changes in lignin content observed by autofluorescence as well as lignin composition observed by the Mäule staining. Although not necessary, it also would be interesting to look at total cellulose content. This data would give a more global context for the reader on the impact of the mutations, compared with just lignin analysis at the base of the inflorescence stem.

RESPONSE: We thank the reviewer for this comment on the reliability of the *in situ* quantitative imaging methods used in our study for determining changes in lignin level and chemistry. The quantitative imaging methods used in our study (Wiesner and Raman) have already been previously validated on set of mutants and natural variation by comparing them with "traditional" lignin biochemical methods such as thioacidolysis and pyrolysis (Agarwal et al., 2019; Blaschek et al., 2020a, 2020b and Yamamoto et al., 2020). As these methods are novel, we nevertheless validated

further our quantitative imaging methods by comparing them with standard biochemical analyses for lignin made on ground stem samples using pyrolysis/GC-MS. We thus analysed one of our quadruple mutant (*Q-5*) and compared it to WT as well as *ccr1-3*, *fah1* and *cad4;5* already used as lignin references in our Raman analyses. As our study focuses on differences between cell types, we also used inducible pluripotent cell cultures to produce high amount of isolated lignified TEs (50/50 mixture of proto and metaxylem). Our biochemical lignin analyses completely confirmed our quantitative imaging by showing (i) a reduction of lignin level in *Q-5*, similar to *ccr1-3*, compared to *fah1* and WT plants, (ii) the increase in S/G ratio in *Q-5* due to a higher reduction of G than S residues, as well as (iii) no changes in the GCHO/GCHOH ratio. Moreover, analyses of isolated TEs confirmed their high lignin content and low S/G ratio, thus confirming our *in situ* chemical quantitative imaging resolution at the cell level. Additionally, these biochemical analyses showed changes in lignin chemistry that could not be detected by our *in situ* methods as previously discussed in the discussion such as H and 5H residue content as well SCHO and SCHOH content. These novel biochemical analyses were added in the paragraphs entitled "LAC paralogs non-redundantly and synergistically affect stem growth" and "Each LAC paralog non-redundantly determines lignin composition in the different cell types" and new Figure 1 and S7 as well as in the discussion.

Point 2. One of the main strengths of this paper is that it focuses on LAC activity and lignin deposition to different cell types and regions of the cell wall, which has had limited attention in the literature thus far. However, based on the methods, most of the microscopy analyses have been performed using 20x-40x objectives. I question whether this is sufficient to get the resolution necessary to identify specific regions of the cell wall. For example, the thickness of the CML and cell wall layers of Arabidopsis are on the nanometer scale, and often the only way to definitively identify secondary cell wall layers (S1/S2/S3) is through TEM techniques. In addition, the stem sections were dehydrated in ethanol, frozen, and then rehydrated prior to analysis. Especially in the case of the hypolignified Q mutant, the prolonged dehydration and then rehydration could change cellular morphology, leading to incorrect estimates of regions of the cell wall. It would be useful to have some higher magnification images showing cell wall thickness and lignin deposition on both dehydrated and fresh samples, as well as more clearly-defined and reproducible methods for how different regions and cell wall layers were identified.

RESPONSE: We thank the review for this comment on the capacity of light microscopy to distinguish between cell wall layers - such as between the compound middle lamella, cell corners and the different S layers of the secondary cell walls. We did not develop these methods but used standard light microscopy methods previously described to distinguish between cell wall layer for lignin accumulation (Donaldson and Knox, 2012 - 10.1104/pp.111.184036), lignin coniferaldehyde content (Blaschek et al., 2020), LACCASE translational fusions (Hoffman et al., 2020), laccase activity (Hiraide et al., 2020) and lignin aromatic types (Morel et al., 2022). We thus used the same criteria as these articles to define the relative position of the different cell wall layers. We however agree that the distinction of the different cell wall layers cannot be unequivocally made using light microscopy for all cell types, but it is very clear for interfascicular fibers on which we (as well as other authors) have focused. We thus added the above references in the corresponding materials and method as well as in the paragraph entitled "LAC activity is cell-type- and substrate-specific". Please also note that in contrast to transmission electron microscopy, the use of light microscopy to analyse cell wall layers in interfascicular fibers enables to acquire larger sample size thus averaging out errors of section orientation and developmental state. Following the reviewer's comment and not to exclusively rely on previous publications, we nevertheless estimated the resolution/magnification necessary to clearly distinguish these cell wall layers using light microscopy in- between Q mutants and WT plants using different objective magnification with different numerical aperture. The comparison of autofluorescence line profiles across tri-cellular junctions in WT and Q mutant cross-sections using 10x, 20x and 40x magnifications showed that good signal/noise and illumination were obtained using 20x to acquire full cross-sections. We thus added this new data as Figure S9 and described briefly these results in the paragraph entitled "Distinct LAC paralogs are responsible for cell wall layer-specific lignification".

We also thank the reviewer for the comment on sample preparation for histological analysis. We agree with the reviewer that sample preparation affects organ dimensions and cellular structure, and there are no ideal method to preserve large amount of samples for exhaustive *in situ* analysis. In fact, fixation with paraformaldehyde disturbs protein localization (Li et al., 2015 - 10.1371/journal.pone.0135033), glutaraldehyde increases autofluorescence (Lee et al., 2013 - 10.1039/C3CC40799C) and organic solvents shrink cell with primary cell walls (Talbot and White, 2013 10.1186/1746-4811-9-36). As our aim was to extract all interfering compounds on LAC activity and lignin spectral properties, we opted for 70% ethanol that enabled protoplast and metabolites extraction as well as fixation to perform and relate all of our different analyses for each genotype in multiple independent replicates. The use of different fixation that would not have removed metabolite or protoplast extraction would have not enabled us to relate our measurements nor be conclusive on cell wall localized LACCASEs. We accordingly added this aspect in the materials and methods as well as in the paragraph entitled "Distinct LAC paralogs are responsible for cell wall layer-specific lignification" and "Each LAC paralog non-redundantly determines lignin composition in the different cell types".

Point 3. Previous studies have demonstrated that the localization of fluorescently-tagged LACs differ between cell types and regions of the cell wall, with LAC4 and LAC17 localizing to the secondary cell wall of xylem vessels and

interfascicular fibers (Hoffmann et al. 2020; https://academic.oup.com/plphys/article/184/2/806/6117902). Interestingly, some results of the lignin analyses/enzyme activities conflict with the known localization of theses enzymes. For example, despite the secondary cell wall localization, Figure 3 shows that LAC17 plays a role in lignification of the cell corners and middle lamella as well as the secondary cell walls in IF. It is unclear how LAC17 could be acting in lignin deposition in a place where the enzyme is not localizing. The authors should comment on possible mechanisms for this in the Discussion. Additionally, the localization of LAC5 and LAC12 has not been elucidated. Localization data of these enzymes (e.g. LAC12 to the CC of IFs) would strengthen the conclusion that they are acting in the particular cell wall domains hypothesized by the data.

RESPONSE: We thank the reviewer for this comment on the localization of the different LACCASE paralogs and overlap of our results with previous published studies. This is an interesting issue as most of the previously published articles only partly overlap in their localization results. This includes differences in transcriptional reporter expression of *LAC17* shown to express in vascular bundles and interfascicular regions by Turlapati et al., (2011 - 10.1007/s00425-010-1298-3) but only in interfascicular fibers by Berthet et al., (2013 - 10.1105/tpc.110.082792). This also affects the localization of LAC17 detected only in the cambium of vascular bundles and in the inner most layer of secondary cell walls of interfascicular fibers using immunolocalisation by Berthet et al., (2013) but exclusively in secondary cell walls of TEs and IFs by Schuetz et al., (2014 - 10.1104/pp.114.245597) and Hoffmann et al. (2020 - 10.1104/pp.20.00473). On a similar note, LAC10 was shown to be localized using RFP translational fusion in the epidermis and trichome by Hoffmann et al. (2020) but exclusive to vascular bundles and interfascicular regions by Turlapati et al., (2011) using transcriptional reporter. Lastly, the localization of specific LAC paralogs, such as LAC4, have been shown to exhibit different cell wall layer localization along developing stems (Hoffmann et al. 2020). In our study, we preferred not to perform gain-of-function studies but defined the localization of the different LACCASE paralog using the spatial changes of phenoloxidase activity between our partly overlapping set of higher order mutants and their changes in lignin chemistry. As mentioned in the discussion, previous studies on LACCASE paralog localization (using proteomics, transcriptional fusions, immunolocalisation or translational fusions) mostly overlap between each other and our study. As for LAC17 role in the middle lamella, we accordingly added in the discussion a mention to the model of the sequential intervention of different phenoloxidases to make specific lignin as proposed by Barros et al., (2015 - 10.1093/aob/mcv046), thus explaining how an enzyme can remotely affect the chemistry of lignin assembly in a layer where it is not present (but monomers need to travel across cell wall layers where LAC17 is present to reach its final accumulation site in the middle lamella of IFs). We nevertheless understand the need to provide additional data supporting the localization in lignifying cells and possibly in multiple cell wall layer, especially for LAC12. To do so, we thus provided new data using gene expression analysis along the synchronous differentiation of TEs using inducible pluripotent cell cultures of *Arabidopsis* (microarray and RT-qPCR). This new data enabled us to show that (1) all LAC candidates selected are highly co-regulation with TE cell death and secondary cell wall biosynthesis marker genes – indicating that LAC5, LAC10 and LAC12 are similarly expressed than LAC4, LAC11 and LAC17, (2) these LAC candidates are restrictively expressed in lignifying conditions in contrast to phenylpropanoid genes, and (3) LAC12 presents an extended expression beyond TE cell death suggesting an expression in both TEs and xylem parenchyma cells that have no secondary cell walls (and thus only a compound middle lamella). This new important data further supports the relevance of LAC5, LAC10 and LAC12 during lignification as well as their slight difference in expression suggesting specific cell wall layer and cell type localization. We thus accordingly revise the text in the paragraph entitled "LAC paralogs non-redundantly and synergistically affect stem growth" and in the discussion as well as provided new figure 1.

Point 4. In Figure 1 it shows the dwarf stem phenotype of the Q mutant. Given the IF were hypolignified, did you observe that the stems would fall over (pendant phenotype), similar to that observed by Smith et al. 2013 (https://academic.oup.com/plcell/article/25/10/3988/6099520) for loss of mechanical strength in the stem tissue?

RESPONSE: We thank the reviewer for this comment on the lodging of LACCASE higher order mutants. We have accordingly used imaged-assisted phenotyping to precisely measure the difference to 90 degree of the angle formed by the base of the longest stem to the soil surface. Note that we did not find a precise method to measure lodging in *Arabidopsis* due to lignin-defects in previously published article (so we only referenced a study made in wheat), and thus hope that our analysis will help setting appropriate standards for this type of measurements. We have thus measured the lodging angle as well as the stem width to height ratio and found that only *Q-10* significantly showed lodging while the other hypolignified mutant promoted their upright stature by increasing their stem width to height. We thus added these new results in figure S2 and presented them in the paragraph entitled "LAC paralogs non-redundantly and synergistically affect stem growth".

Point 5. In Figure 2, the authors show that different LAC isoforms have different abilities to oxidize artificial substrates in a given cell type or region of the cell wall. Thus far in the literature, these enzyme assays

have mostly been performed in-vitro. While the method in the manuscript is more representative of the native cell wall environment of the enzymes, the stem sections used for analyses were dehydrated and stored at -20C for extended

periods of time. Are these stem sections truly representative of "native conditions" (as stated in Lines 509-511)? The text should be adjusted to include for the possibility that ethanol washing removed soluble components of the cell wall that may be important for proper lignification dynamics. Would the oxidative activity data change if performed on fresh stem sections?

RESPONSE: We thank the reviewer for this comment on the enzymatic essay used to monitor LACCASE activity. Histological analyses of phenoloxidases activity have already been made on plant cross-sections of multiple plant species using artificial substrate (*Arabidopsis* for PRXs by Hoffman et al., 2020 - 10.1104/pp.20.00473; in *Zinnia* for LACs and PRXs by Liu et al., 1994 - 10.1046/j.1365- 313X.1994.6020213.x; in poplar for LACs by Ranocha et al., 1999 - 10.1046/j.1432-1327.1999.00061.x; and for LACs and PRX in *Chamaecyparis* by Hiraide et al., 2021 - 10.1111/nph.17264) but without a real- time monitoring necessary for the user to distinguish between enzymatic oxidation, saturated activity and unspecific binding. We wanted to measure only cell wall localised LAC activities, to do so we ethanol- cleared and rehydrated our sections to remove interfering metabolites (acting as oxidation mediators causing artefacts; Blaschek and Pesquet, 2021), contaminating intracellular phenoloxidases (such as PRXs causing artefacts; Blaschek and Pesquet, 2021) and co-substrates for cell wall PRXs (H2O2 produced by wounding or hypoxia; Liu et al., 1999) as well as ensured that synthetic substrates were similarly accessible to all cell types and cell wall layers. In addition, the use of fresh sections treated with catalase to remove H2O2 would have caused local increase of O2 concentration also affecting LAC activity. To clearly assign changes to LACs, we extracted all metabolites as we could not distinguish between possible physiological extracellular redox mediators and contaminants released by sectioning. We thus accordingly revised that "native conditions" is an overstatement and changed it to “embedded in cell walls”. We thus accordingly revised the discussion.

Point 6. While there may be more favoured substrates for LACs in different cell wall regions, there is still substantial flexibility in this process, observed especially in monolignol biosynthetic mutants (such as fah1-2 in this study) that have predominant deposition of a certain type of monolignol. It would be useful to incorporate into the Discussion how LACs with certain substrate specificities could be flexible enough to facilitate different lignin compositions based on monolignol biosynthesis.

RESPONSE: We thank the reviewer for this comment and have accordingly revised the discussion to explain LACCASE could oxidise other compounds than their preferred substrates and referenced our recent review (Blaschek and Pesquet, 2021 - 10.3389/fpls.2021.754601) that cataloged such substrate range. Moreover we explained that the resulting lignin in higher order mutants would results from the combined effect of reduced/abolished substrate specific oxidation together with modifications due to unspecific oxidation by the remaining LACs and PRXs (Blaschek and Pesquet, 2021), reduction in diffusion of some lignin precursors to cell walls due to altered metabolic gradients created by oxidation (Perkins et al., 2022) and feedback modifications of intracellular phenolic metabolism (Vanholme et al., 2022).

<u>Reviewer #3:</u>

In this paper, the authors described 5 laccase (LAC4, LAC5, LAC10, LAC12 and LAC17) and their functions in lignin composition in different cell types and wall layers in Arabidopsis. The authors obtained very important research materials with different gene mutations and combinations of the five laccase genes. Meanwhile, the research methods mentioned in this paper like image-based genotyping and different quantification analysis of laccase activity and lignin composition are inspiring and vital. Based on the histochemical and quantitative analysis, the authors explained the spatial control of lignin composition by different combinations of laccases in different cell wall layers. But I have some major issues with the experiment designs, results display and conclusions they made. The major issues will be listed below.

Point 1. Can the authors provide gene expression patterns which indicated gene mutation in different laccase-mutants in line 156-line 157?

RESPONSE: We thank the reviewer for this comment on the gene expression levels in each mutant. We have accordingly added expression analyses using RT-PCR and RT-qPCR on the single mutants as well as extended Table S1 with the previously performed analysis on LACCASE gene expression. We thus observed (and confirmed) that each single mutant effectively reduced the expression of their corresponding LACCASE paralog without altering the expression of other LAC paralog. This suggested no compensatory regulation between LACCASE paralog. We added this data as figure S1, table S1 as well as added reference to it in the paragraph entitled “LAC

paralogs non-redundantly and synergistically affect stem growth".

Point 2. In line 163, the authors described the growth phenotypes of WT, Q mutant and lac mutations. Can the author provide more detailed statistic results of time of bolting, stem growth rate, final height and siliques, with specific analyzed numbers instead of the percentage? In line 164, "the reduced growth rate and final height were restored to WT levels. ", please provide the growth rate and final height number in wild type background. The same with the silique length in line 166. Also in line 164, there should be "figure S2D" instead of "figure S1D".

RESPONSE: We thank the reviewer for this comment on the phenotypic analysis of our higher mutant series. We accordingly provided stem and leaf growth rate in our new figure 2 as well as their statistical analysis using one-way ANOVA and accordingly added the statistical analysis (in the figure as well as in the corresponding supplementary data) on the bolting time. We nevertheless kept the percentage of growth for stem length to the WT between single and multiple mutants to normalize our measurements as they were collected and grown in different conditions during the past 8 years as we were stacking our higher-order mutants. We moreover added other parameters including basal stem width, number of stems as well as angle of the basal stem to the soil. All measured parameters are added to supplementary data and final stem height of the WT was added in the paragraph entitled "LAC paralogs non-redundantly and synergistically affect stem growth".

Point 3. In line 177, can the author provide the reference and definition of "oxidation rates"? In line 178, can the author explain why used "ethanol-cleared and rehydrated stem cross-sections"? From Hiraide et al., 2021, I think they used freshly cutted stem cross-sections. Is there any laccase activity difference between the freshly cutted cross-sections and ethanol-treated samples? What is the pH for DAF and DAB oxidation rate in figure 2B and 2C? It was not mentioned in manuscript and method.

RESPONSE: We thank the reviewer for this comment and have accordingly provided a definition of oxidation rate in the materials and methods as "The oxidation rates of each substrate in each cell type and cell wall layer corresponded to the slope of the linear changes in intensity of the oxidized product during time". We thus adapted the LAC oxidation rate as described by Sterjiades et al., (1991 - 10.1104/pp.99.3.1162) as change in absorbance during time to histological sections. We used a different sample preparation than Hiraide et al. (2021) as we wanted to have real-time measurements (so we made the section first and then provided the substrates, instead of providing the substrate and then performing the sections) to clearly distinguish between enzymatic oxidation, saturated activity and unspecific binding. We also wanted to only measure cell wall localised LAC activities, to do so we ethanol-cleared and rehydrated our sections to remove interfering metabolites (acting as oxidation mediators causing artefacts; Blaschek and Pesquet, 2021), contaminating intracellular phenoloxidases (such as PRXs causing artefacts; Blaschek and Pesquet, 2021) and co-substrates for cell wall PRXs (H_2O_2 produced by wounding or hypoxia; Liu et al., 1999) as well as ensured that synthetic substrates were similarly accessible to all cell types and cell wall layers. In addition, the use of fresh sections treated with catalase to remove H_2O_2 would have caused local increase of O_2 concentration also affecting LAC activity. We thus accordingly revised the paragraph entitled "LAC activity is cell-type- and substrate-specific" and added the pH used in new Figure 3B and 3C.

Point 4. In figure 2B and 2C, what are the oxidation rate for autoclaved Q4, Q5, Q10, Q10 and Q12? The images in Figure S3 in Q mutant, the resolution was low. Can the authors provide images included vascular bundles and better resolution for quantification? Can the author provide phloroglucinol staining to better indicate SCW composition?

RESPONSE: We thank the reviewer for this comment on the phenoloxidase activity. We have accordingly added the oxidation rate of the autoclaved sections (combining WT and mutants, which did not exhibit significant difference as shown in figure S3) on new Figure 3B and 3C. We also included additional images with better resolution and using different substrates in Figure S3 as well as large Wiesner and Mäule stained images of the different genotypes used in this study in Figure S4.

Point 5. In figure 3, can the authors provide better resolution images for Maule and Wiesner staining images in stem cross-sections? In line 225, the authors described "lac mutants showed weaker red staining in IFs of different Q mutants. Please be more specific and quantitative. Please indicate the IF regions in Maule staining. In line 240, please provide quantitative results to indicate" largely reduced". Is "higher-order mutants" an term used in plant researches?

RESPONSE: We thank the reviewer for this comment and have accordingly provided in Figure S4 bigger images for readers to examine. As the Mäule test has not been validated as a quantitative in situ method for lignin, we initially

avoided to quantify such changes. However based on the reviewer's comments, we accordingly quantified changes in grey-scale intensity although we cannot really define what this means (these changes are now added in Figure S5 and numerical fold changes are provided in the text). We additionally also performed regression analyses between the hue of the Maüle test (color shift from red to brown) and Raman S/G content, and were able to suggest that the Mäule test hue is capable of linearly semi-quantifying S/G content with subcellular resolutions. We added this in Figure S5 and in the paragraph entitled "LAC activity is cell-type- and substrate-specific" although we did not over-interpret this observation that will benefit other researchers using this method.

We have not coined the term "higher order mutants" which corresponds to mutants with two or more insertional modifications as previously used in Plant Biology by van Kleef et al. (2014 - 10.1093/jxb/eru338), Barbato et al., (2020 - 10.1038/s41598-020-62717-1) and Rojas-Murcia et al., (2020- 10.1073/pnas.2012728117). We thus provided these references and a short definition in the paragraph entitled "LAC paralogs non-redundantly and synergistically affect stem growth".

Point 6. In figure 4A, the authors used Raman microspectroscopy to quantify lignin composition. Starting from Line 260, "the lignin S/G ration was highest in IF" and "lignin GCHO/GCHOH was highest in PXs". Can the authors provide quantitative results instead of "highest"? In line 263, "lignin to cellulose amount was reduced. ", please provide statistic number and analysis here. Also the same issues for " lignin to cellulose ratios lower than ccr1-3" in line 271, "lignin accumulation less than Q-12" in line 273 and "Q-10 and Q-12 showed a slight increase" in line 277. Please provide statistic results since the authors already did quantitative analysis. The lignin composition results were important parts in the manuscript. Can the author provide other methods to solidify the results by Raman micrsospectroscopy, for example by fluoresecen-probes in histochemical experiments?

RESPONSE: We thank the reviewer for this comment on our quantitative imaging methods. We had already validated the reliability and semi-quantitative linearity of these diagnostic Raman bands in Blaschek et al. (2020 - 10.1021/acssuschemeng.0c00194) and Yamamoto et al., (2020 - 10.1002/cssc.202001242) with standard bulk lignin biochemical analyses such as pyrolysis and thioacidolysis/GC-MS. Other research groups, also showed the semi-quantitative linearity of Raman to measure S/G in lignin of different plant species (Agarwal et al., 2019). In addition to Raman, we validated our Raman analysis using complementary histochemical methods to show changes in GCHO (using the Wiesner test – method validated by Blaschek et al., 2020 - 10.3389/fpls.2020.00109). Additionally in the revised version, we also added new regression analyses between the hue of the Mäule test and Raman determined S/G that showed a strong linear correlation and confirmed again our changes in S/G observed by Raman – we added this new results in the paragraph entitled "Each LAC paralog has cell type-specific effects on lignification" as well as in Figure S5. We decided to use only direct methods of lignin in situ analyses rather than indirect labelling methods, such as fluorescent probe feeding, to directly analyse lignin composition in situ using multiple methods (Raman, UV-autofluorescence and histochemistry). Nevertheless as these in situ quantitative methods are relatively new, we understand the need for further validation and have thus analysed lignin amount and chemistry using pyrolysis/GC-MS on ground stems of Q-5 compared to WT and our reference phenylpropanoid mutants. These new results validate our measurements using Raman in higher-order lac mutants such as (i) reduction in lignin amount, (ii) increase in S/G due to a greater reduction in G than S residues, (iii) reduction in total GCHO, and (iv) no change in GCHO/GCHOH. These biochemical analyses moreover showed that aspects of lignin chemistry that cannot currently be quantitatively measured using Raman such as H and 5H residues (increasing in Q-5), and SCHO and SCHOH (unchanged in Q-5 in contrast to our reference mutants). We thus accordingly added these new results in the paragraph entitled "Each LAC paralog non-redundantly determines lignin composition in the different cell types" as well as in Figure S7.

Point 7. In line 391, please quantify the IF SCW thickness result and difference in WT and all the mutants, not using "significantly thicker". Can the author provide reference about the SCW thickness measurement? In figure 7A, why the dash-line indication in Q5, Q10, Q12 and Q was different from the rest?

RESPONSE: We thank the reviewer and have now provided fold changes instead of using "significantly thicker". We have used light transmission microscopy to estimate cell wall thickness similarly to previously published by Falcioni et al., (2021 - 10.3390/plants11070995) and Schumann et al., (2020 - 10.1007/s00425-020-03494-z). We added a dash-line to delineate the distance between the lumens of two neighboring fibers, which is accordingly thicker for Q-5, Q-10, Q-12 and Q as we describe in the result paragraph entitled "Specific LAC paralogs control lignification to control fibre swelling capacity". We nevertheless revised the legend of Figure 8 to explain the difference in dashed line size.

Point 8. For the introduction part, the authors mentioned a lot about lignin chemistry and composition. The introduction of laccase proteins only started in line 114. The introduction of laccases can be added with more previous studies in lignin deposition.

RESPONSE: We thank the reviewer and have accordingly revised the introduction to present previous breakthroughs on the role of LACCASES and lignin formation. Previous publications focused on the role of certain LACCASE paralogs on lignin amount in whole stems rather than between the different cell types of stems. The aim and novelty of our research is to understand the mechanisms enabling spatial and cell specific control of lignin chemistry, so we keep a strong background on cell types, cell wall layers and their difference in lignin chemistry.

Point 9. I would recommend the authors to provide a demonstration of the specific functions or contributions in different cell layers of five laccases to summarize and better understanding.

RESPONSE: We thank the reviewer for this comment and have accordingly provided a demonstration of the non-redundant functions of the different LACCASE paralogs. We thus performed a principal component analysis (PCA) to demonstrate that specific combination of LACCASE paralogs alters lignin chemistry differently in a cell types/morphotype. We accordingly added this new figure in Figure S8, defined the analytical method as well as described these results in the paragraph entitled "Each LAC paralog non-redundantly determines lignin composition in the different cell types" as well as in the discussion. In addition to our summarizing table, we also included a graphical abstract to visually summarise our findings.

TPC2022-RA-00433R1 2nd Editorial decision – *accept with minor revision* September 26, 2022

We have received reviews of your manuscript entitled "Different combinations of laccase paralogs non-redundantly control the lignin amount and composition of specific cell types and cell wall layers in Arabidopsis." On the basis of the advice received, the board of reviewing editors would like to accept your manuscript for publication in The Plant Cell. This acceptance is contingent on revision based on the comments of our reviewers. Please revise the manuscript to the best of your ability according to all of the reviewer comments; in particular, reducing the length of the Discussion could help make the manuscript more readable, concise, and citable.

Please also note that this revision allows you to add a citation of the published Menard article and to correct the typo regarding the Vanholme 2012 citation.

Please highlight all changes and include a detailed annotation of changes of the text, with line numbers, and noting your responses to the comments.

TPC2022-RA-00433R2 2nd Revision received October 14, 2022

Reviewer comments and **author responses**:

Reviewer #1:

I am very satisfied with the authors' responses to my comments (and to those of the other reviewers). In some cases, they have provided additional data to further substantiate their arguments and in others they have convinced me of their differing views concerning my original critiques. The authors should be commended for an extraordinarily ambitious piece of work that is backed up with such thorough quantitative analyses.

Point 1. If anything remains to be commented upon, it might be that the manuscript is very long. A reduction in the length of at least the Discussion could be made I think without loss of impact.

RESPONSE: We thank the reviewer for this comment, and we have accordingly shorten by more than half our discussion by removing comments on *in situ* quantitative method and their capacity to remove averaging errors compared to bulk analyses. We also shorten the overlap in the localization between studies, removed the spatial localization between primary and secondary cell walls of lignin during evolution and specie adaptation as well as the role of pH. We fell that the shorten discussion still captures the key discussion and impact of our article as well as the previous comments from the reviewers.

Reviewer #2:

In the re-revision, Blaschek et al. have included several edits that add greatly to the manuscript. In particular, their addition of the LAC expression data in Figure 1 adds context to why they chose particular LAC isoforms, and the pyrolysis/GC-MS data of Q-5 strengthens confidence in their microscopy quantification of lignin. I also think that their revision of the language improves comprehension of the overall conclusions of the manuscript.

Reviewer #3:

The authors indeed spent lots of efforts and deep thoughts in this manuscript. The authors described the biochemical functions of LAC4, LAC5, LAC10, LAC12 and LAC17 in lignin composition and cell wall structure in Arabidopsis. It is quite difficult and beneficial to obtain the combined mutations of these genes. Another significance of this paper is that the authors utilized delicate and inspiring quantification methods in laccase activity, plant growth and cell-wall compositions. This paper comprehensively described the fundamental biochemical functions of 5 secondary cell wall-related laccase in Arabidopsis. The experiments are well-designed and the results are strongly reliable, which support the conclusion that different combinations of laccase showed specific functions in different cell types and cell wall layers. But I had some majors concerns with the manuscript organization and experiments as listed below.

Point 1. I think this manuscript can be written in a more precise and concise way. The manuscript mainly talked about three parts: introduction of laccase mutation combination and quantification of laccase activities, lignin composition in laccase mutants and impacts on cell wall structure in laccase mutants. I had issues with second part and the authors described more than sufficient information starting from line 496 to line 692 in 7 pages. I appreciated that the authors did histochemical staining, Raman microspectroscopy and pyrolysis/GC-MS to check lignin monomer compositions and residues in different lignified cell types in the mutants. But I recommend summarizing the information from these 7 pages and be more concise and accurate. Also the last two sections from line 694 to line 745 mainly discussed laccase mutations affects secondary cell wall structure and reduced mechanical supports for plants. I think these two sections can be combined into one part to strongly support the conclusion.

RESPONSE: We thank the reviewer for this comment and have accordingly reorganized and reduced the length of our article by (i) segmenting more the results into individual paragraphs, (ii) fusing overlapping results using different method (i.e. histochemistry and Raman) into the same paragraph, and (iii) shortening the discussion by more than half by removing comments on in situ quantitative method and their capacity to remove averaging errors compared to bulk analyses.

Point 2. The authors described the different combinations of laccase genes in specific cell types during secondary cell wall deposition timepoints. But all the mutants were written in code through the whole paper. The interpretation of mutant combination in line 424 is needed all the time when I went through the paper. I read the explanation in "Material& methods" in line 168. But the complexity of mutant name made it difficult and confusing to remember all the "Q-4, Q-5, Q-10, Q-12, Q-17" and future reference of the results in this paper.

RESPONSE: We thank the reviewer for this comment and we agree that it is not trivial to keep track of the mutated or wild-type alleles of the different paralogs in five different quadruple mutants. Unfortunately there are only two ways of naming the various genotypes in the text such as "Q-12" or "lac4 lac5 lac10 lac17". These two naming systems both have issues as "Q-12" doesn't immediately remind the reader of the mutated loci that are shared with the quintuple mutant, while "lac4 lac5 lac10 lac17" obscures which locus differs from the quintuple mutant. The purpose of the quadruple mutants in this work is to highlight the functions of the paralog that is not mutated — i.e. the one that differs from the quintuple mutant. For this reason we maintain that the "Q-12" annotation is ¬– although not perfect – more focused and easier to read than the "lac4 lac5 lac10 lac17" annotation. We nevertheless revised the text to make the reading easier by defining phenotypic difference between quadruple using "in all quadruple lac mutants except …" and we also added Table S2 summarizing the correspondence between mutated alleles and our Q-X names.

Point 3. In figure 1C, what are meaning of "a" "b" and "c"? I did not find annotations for them in the figure context and "Material & methods". The same issue with rest of the figures in the manuscript, like figure 2B, figure 3B and C......

RESPONSE: We thank the reviewer for this comment and have accordingly explained that the letter coding corresponded to our statistical test - and thus added it in the legend of Figure 1 as previously done in the legends of

other figures.

Point 4. The supplement figure S1B and S1C, it is easier to change the real time PCR primers for LAC5, LAC10 and LAC12 rather than using RT-PCR to check the full transcripts. Another question is that there is not endogenous control for RT-PCR for cDNA quality in figure S1C.

RESPONSE: We thank the reviewer for this comment and have accordingly added semi-quantitative RT-PCR for the XCP1 transcripts to show an endogenous control of cDNA amplification in figure S1C. We have used different primer pairs for LAC10 and LAC12 that were either upstream or downstream of the T-DNA insertion, and both showed that truncated transcripts were produced unlike other mutants that annulled or knock-down transcript abundance – nevertheless our semi-quantitative RT-PCR to amplify fragments spanning across the T-DNA confirmed the absence of fully functional transcripts.

Point 5. In figure 2D, the plant height was reduced in Q compared to WT. I am curious about the plant height was measured by real height (because the mutant Q might be pendent as shown in figure S2H) or was measured by physically linearize the whole plants. This information is not mentioned in the manuscript.

RESPONSE: We thank the reviewer for this comment and have accordingly revised the figure legend and the materials and methods to clarify that the kinetic analysis using image analysis enabled to measure the "projected stem height" (from the straight line from rosette to highest point of the plant from the picture, which could be affected by plant straightness) in contrast to "stem height" measured at harvest (on the stretched out stem manually measured to be unaffected by plant straightness).

TPC2022-RA-00433R2 3rd Editorial decision – *acceptance pending* October 27, 2022

We are pleased to inform you that your paper entitled "Different combinations of laccase paralogs non-redundantly control the lignin amount and composition of specific cell types and cell wall layers in Arabidopsis" has been accepted for publication in The Plant Cell, pending a final editorial review by a science editor. At this stage, your manuscript will be evaluated by a science editor with respect to its presentation of scientific content, compliance with journal policies, and presentation for a broad readership.

Final acceptance from Science Editor November 23, 2022
